# An atlas of RNA base pairs involving modified nucleobases with optimal geometries and accurate energies

**DOI:** 10.1093/nar/gkv606

**Published:** 2015-06-27

**Authors:** Mohit Chawla, Romina Oliva, Janusz M. Bujnicki, Luigi Cavallo

**Affiliations:** 1 King Abdullah University of Science and Technology (KAUST), Physical Sciences and Engineering Division, Kaust Catalysis Center, Thuwal 23955-6900, Saudi Arabia; 2 Department of Sciences and Technologies, University Parthenope of Naples, Centro Direzionale Isola C4, I-80143, Naples, Italy; 3 Laboratory of Bioinformatics and Protein Engineering, International Institute of Molecular and Cell Biology in Warsaw, ul. Ks. Trojdena 4, 02-109 Warsaw, Poland; 4 Laboratory of Bioinformatics, Institute of Molecular Biology and Biotechnology, Faculty of Biology, Adam Mickiewicz University, Umultowska 89, 61-614 Poznan, Poland

## Abstract

Posttranscriptional modifications greatly enhance the chemical information of RNA molecules, contributing to explain the diversity of their structures and functions. A significant fraction of RNA experimental structures available to date present modified nucleobases, with half of them being involved in H-bonding interactions with other bases, i.e. ‘modified base pairs’. Herein we present a systematic investigation of modified base pairs, in the context of experimental RNA structures. To this end, we first compiled an atlas of experimentally observed modified base pairs, for which we recorded occurrences and structural context. Then, for each base pair, we selected a representative for subsequent quantum mechanics calculations, to find out its optimal geometry and interaction energy. Our structural analyses show that most of the modified base pairs are non Watson–Crick like and are involved in RNA tertiary structure motifs. In addition, quantum mechanics calculations quantify and provide a rationale for the impact of the different modifications on the geometry and stability of the base pairs they participate in.

## INTRODUCTION

Discovery of various forms of noncoding RNAs in the past two decades, besides the well-known coding messenger RNA (mRNA), ribosomal RNA (rRNA) and transfer RNA (tRNA), has dramatically changed our view of the RNA function. In addition to the transmission of genetic information, it is indeed now clear that RNA molecules can fulfill a variety of other functions, including catalysis and translational regulation, up to the tuning of cellular differentiation and development. It is particularly interesting that the fraction of human genome that is cell-specifically transcribed to generate these regulatory noncoding RNAs is larger that the fraction of it devoted to encode proteins (1).

RNA fulfills this striking variety of functions apparently based on a limited chemical diversity, established by only four nucleobases: adenine (A), guanine (G), cytosine (C), uracil (U). This apparent contradiction is solved when thinking that RNA can take advantage of a large number of posttranscriptional modifications, greatly enhancing its chemical information. To date, more than 100 different modifications have been reported in RNA molecules, ranging from simple additions or substitutions of chemical groups as e.g. in methylations or deaminations, to complex alterations, often comprising a series of reactions, some of which even resulting in a different heterocyclic structure. A complete catalogue of such modifications can be found in dedicated databases, such as the RNAmods database (2) and MODOMICS (3), with the latter database containing also information about RNA modification pathways and sites of modification in selected RNAs.

While the highest concentration and diversity of posttranscriptional modifications has been till now reported in tRNA molecules, they are also widespread in rRNA and mRNA, and more than a dozen of modifications have already been reported in small, noncoding RNAs (2,4–8). As a matter of fact, nowadays most if not all the major classes of RNA molecules in the cell are thought to possibly present modified nucleotides.

Specific modifications contribute to tRNA stability, favor its recognition by the cognate aminoacyl synthetase and by mRNA, influence nuclear export of mRNA, protect it from degradation and regulate splicing, or can establish resistance to antibiotics in bacterial rRNA (9–14). Many more examples of the impact of modifications on the RNA function and structure are reviewed in (8,13–17). Importantly, modifications also play a role in human diseases, particularly tumors, myopathy, type-2 diabetes and obesity [reviewed in (18)].

**Figure 1.**
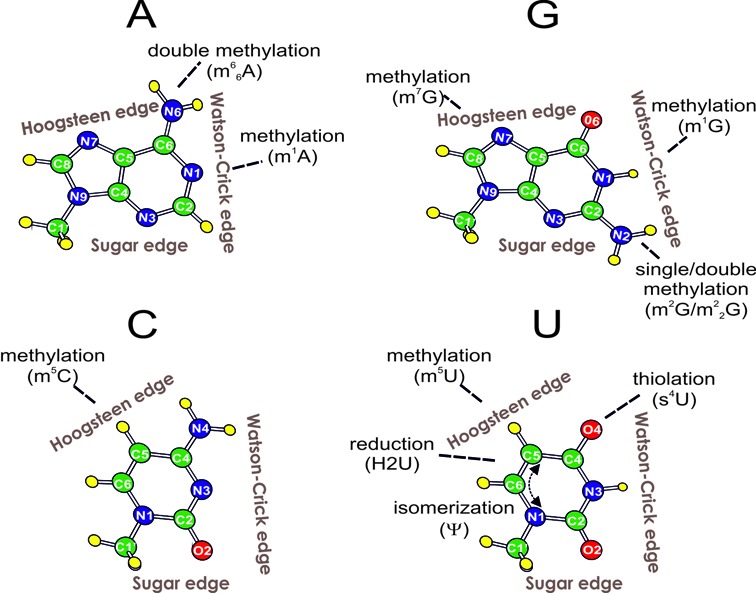
Schematic representation of the nucleobase modifications involved in H-bonded base pairs in experimentally determined RNA structures. Nucleobases are oriented with their Watson–Crick edge facing right. The two non-canonical edges for H-bonding, Hoogsteen and sugar, are also indicated.

Chemical modifications that control the stability and proper folding of the RNA molecule are generally classified as ‘structural’. The most efficient ways by which they can affect the RNA structure are hydrogen bonding, π-stacking and the coordination of metal ions, with the first one playing a major role. Chemical modifications may actually occur at all the three edges used by nucleobases for H-bonding to other bases, i.e. the Watson–Crick, the Hoogsteen and the sugar edge (see Figure 1). A modified nucleobase can thus exhibit significantly changed pairing properties, as compared to the corresponding canonical one. If the Watson–Crick edge is affected, for instance, the canonical Watson–Crick G-C/A-U pairing will be impaired, while non canonical base pairs, involving either of the other two edges, may be favored.

To date, more than 3000 macromolecular structures have been deposited in the wwPDB (19), which contain different types of RNA molecules including not only tRNAs, mRNAs, rRNAs, but also viral RNAs, riboswitches, ribozymes and more recently discovered small non coding nuclear and nucleolar RNAs. Remarkably, a significant fraction of such structures present modified residues. Therefore, it is time to systematically investigate the structural effect of chemical modifications in the context of experimental RNA structures. Herein we will focus on the modifications effect on H-bonded base pairs. To this end, we performed a comprehensive search in the Protein Data Bank (19) to compile an atlas of experimentally observed ‘modified base pairs’, i.e. H-bonded base pairs, with a given geometry, involving at least one noncanonical nucleobase. For each specific combination of nucleobases and base pair geometry, we recorded the occurrences and selected a representative from the highest resolution crystal structure presenting it, for subsequent energetic calculations. We came up with an atlas of 27 unique modified base pairs containing naturally occurring modifications, differing by the nucleobase combination and/or base pairing geometry. Nine additional pairs involving non-natural modified bases, specifically halogenated pyrimidines used to solve the crystallographic phase problem, were also retained in our analysis, as the question has been raised whether this kind of modification can interfere with the functional RNA folding (20).

Optimal geometries and accurate interaction energies have been evaluated for all the above H-bonded base pairs, including ribose C1′ atoms. Advanced quantum mechanics methods are indeed especially suitable for the evaluation of the strength of H-bonded bases interaction (21–36). In all cases, we also considered the corresponding pairs involving unmodified bases, in order to allow a comparison of their geometry and energetics. As aforementioned, we are aware that modifications can also impact other properties of the bases, for example their stacking capability (37–41). However, this is out of the scope of the present work.

This study thus provides both an atlas of the modified base pairs experimentally observed to date, with relative occurrences, and an accurate estimate of the effect of each chemical modification on the structure and stability of the corresponding H-bonded base pair. Notably, we found that the modified base pairs typically exhibit non canonical geometries (i.e. different from the classical Watson–Crick pairing) and are located in a variety of different RNA molecules and structural motifs. This extends our understanding of how posttranscriptional modifications act on the structure of RNA molecules to influence their function.

## MATERIALS AND METHODS

### Nomenclature

The adopted nomenclature for the geometry of the analysed H-bonded base pairs (Table 1) is based on that proposed by Leontis and Westhof (42,43) and extended by Lemieux and Major (44). In it, the interacting edges involved in the H-bonding, i.e. Watson–Crick, Hoogsteen or sugar, and the two mutual orientations of the glycosidic bonds, i.e *cis* or *trans* are specified (42,43). A symbol ‘W’, ‘H’ or ‘S’, is given to indicate that the ‘Watson–Crick’, ‘Hoogsteen’ or ‘sugar’ edge is involved in the base-base H-bonding interaction; ‘Bs’ is used for bifurcated base pairs involving the sugar side amino/keto group (44). This is preceded by ‘c’ or ‘t’, indicating that the orientation of the glycosidic bonds is *cis* or *trans*, respectively. We added an ‘r’ in brackets after the edge symbol when the corresponding ribose was also involved in H-bonding. The symbol for the edge H-bonding with the ribose of the paired nucleoside was also reported in brackets, if different from that involved in base-base pairing. Traditional abbreviations were adopted for the modified nucleobases. For the non-natural modifications, after the number of the modified atom we reported the chemical symbol of the halogen element substituting a hydrogen atom and the one-letter-code of the corresponding nucleobase. When a base pair is characterized by only one H-bond, this is indicated by a ‘1’ after the edge symbols.

**Table 1.** Modified base pairs with relative base pairing geometry (Geom.), occurrences (Occ.), position in the selected X-ray structure and chain (Pos./chain), RNA molecule and source

	Nt symbol; name	Mod-bp	Geom.	Occ.	Pos./chain	PDB-ID; res (Å)	RNA	Source
	Adenine							
1	m^1^A; 1methyl A	m^1^A:A	tHW	1	58: 54 (A)	1YFG; 3.00	tRNA(iMet)	*S. cerevisiae*
2	m^1^A;1methyl A	m^1^A:U	tHW(w)	44	628:624;w3446 (0)	1VQ5; 2.60	23S rRNA	*H. marismortui*
3	m^1^A;1methyl A	m^1^A:U	tHW	1	58:54 (B)	1OB2; 3.35	tRNA(Phe)	*E. coli*
4	m^1^A;1methyl A	m^1^A: m^5^U	tHW	19	58:54 (A)	1EHZ; 1.93	tRNA(Phe)	*S. cerevisiae*
5	m^6^_6_A;N6,N6-Dimethyl-A	m^6^_6_A:G	tS(w)S(r)	1	76: 2618 (4:0)	1VQ6; 2.70	23S rRNA	*H. marismortui*
	Guanine							
6	m^1^G; 1-methyl G	m^1^G:C	tHH1	1	9:23 (A)	1YFG; 3.00	tRNA(iMet)	*S. cerevisiae*
7	m^2^G; N2-methyl G	m^2^G:U	cWW	1	6:67 (A)	1FIR; 3.30	tRNA(Lys,3)	*B. taurus*
8	m^2^G; N2-methyl G	m^2^G:C	cWW	23	10:25 (A)	1EHZ; 1.93	tRNA(Phe)	*S. cerevisiae*
9	m^2^G; N2-methyl G	m^2^G:C	cWW1	3	10:25 (B)	1OB5; 3.10	tRNA(Phe)	*E. Coli*
10	m^2^_2_G;N2,N2dimethyl G	m^2^_2_G:A	cWW	20	26:44 (A)	1EHZ; 1.93	tRNA(Phe)	*S. cerevisiae*
11	m^7^G; 7-Methyl G	m^7^G:C	cWW	7	527:522 (A)	4DR2; 3.25	16s-rRNA	*T. thermophilus*
12	m^7^G; 7-methyl G	m^7^G:G	tWH	27	46:22 (A)	1EHZ; 1.93	tRNA(Phe)	*S. cerevisiae*
	Cytosine							
13	m^5^C; 5-methyl C	m^5^C:G	cWW	57	40:30 (A)	1EHZ; 1.93	tRNA(Phe)	*S. cerevisiae*
14	m^5^C; 5-methyl C	m^5^C:G	tWW	3	548:515 (Y)	2DLC; 2.40	tRNA (Tyr)	*S. cerevisiae*
	Uracil							
4	m^5^U; 5-Methyl U	m^5^U: m^1^A	tWH	19	54:58 (A)	1EHZ; 1.93	tRNA(Phe)	*S. cerevisiae*
15	m^5^U; 5-Methyl U	m^5^U:A	tWH	38	654:658 (B)	1C0A; 2.40	tRNA(Asp)	*E. Coli*
16	m^5^U; 5-Methyl U	m^5^U:G	tWH1	2	54:58 (T)	1H4S; 2.85	tRNA(Pro)	*T. thermophilus*
17	m^5^U; 5-Methyl U	m^5^U:G	cWW	2	1:10 (D:B)	1U6B; 3.10	Ribozyme^a^	*Azoarcus* sp.*BH72*
18	s^4^U; 4-Thio U	s^4^U:A	tWH	21	608:614 (B)	1C0A; 2.40	tRNA(Asp)	*E. coli*
19	H2U; 5,6 di-hydro U	H2U:U	tWW	12	916:959 (C)	1IL2; 2.60	tRNA(Asp)	*S. cerevisiae*
20	H2U; 5,6 di-hydro U	H2U:G	cHS1	1	620:619 (B)	1C0A; 2.40	tRNA(Asp)	*E. coli*
21	H2U; 5,6 di-hydro U	H2U:G	tWS	1	20A:15 (T)	1SER; 2.90	tRNA(Ser)	*T. thermophilus*
22	Ψ; Pseudouracil	Ψ:A	cWW	24	6:21 (A:B)	3CGP; 1.57	U2 snRNA	Mammalian^1^
23	Ψ; Pseudouracil	Ψ:A	cHW	1	39:31 (D)	1TTT; 2.70	tRNA(Phe)	*S. cerevisiae*
24	Ψ; Pseudouracil	Ψ:U	tWW	45	2621:1838 (0)	4HUB; 2.40	23S rRNA	*H. marismortui*
25	Ψ; Pseudouracil	Ψ:G	tBsW	48	955:917 (C)	1IL2; 2.60	tRNA(Asp)	*S. cerevisiae*
26	Ψ; Pseudouracil	Ψ:G	cWW	14	6: 20 (A:B)	3CGS; 1.65	U2 snRNA	Mammalian^b^
27	Ψ; Pseudouracil	Ψ:C	cS(r)W	7	516:519 (A)	4DR2; 3.25	16S rRNA	*T. thermophilus*
	Non-natural							
1’	5BrC; 5-Bromo C	5BrC:G	cWW	16	3:29 (A:B)	1QBP; 2.10	synthetic	N/A
2’	5BrU; 5-Bromo U	5BrU:A	cWW	95	2:22 (A)	1ZCI; 1.65	viral genome	*HIV-1*
3’	5BrU; 5-Bromo U	5BrU:G	cWW	16	142:155 (B)	1JID; 1.80	SRP RNA	*H. sapiens*
4’	5BrU; 5-Bromo U	5BrU:5BrU	cWW	2	5:21 (C)	1RLG; 2.70	snoRNA (box C/D)	*A. fulgidus*
5’	5BrU; 5-Bromo U	5BrU:A	cWH	1	38:7 (A)	1KH6; 2.90	viral genome	*HCV*
6’	5BrU; 5-Bromo U	5BrU:A	cS(r)H	1	37:6 (A)	1KH6; 2.90	viral genome	*HCV*
7’	5BrU; 5-Bromo U	5BrU:A	cW(r)S	1	11:26 (A)	1F1T; 2.80	RNA aptamer^c^	N/A
8’	5IU; 5-Iodo U	5IU:A	cWW	13	13:2 (D:C)	464D; 1.23	synthetic	N/A
9’	5IU; 5-Iodo U	5IU:G	cWW	4	6:4 (C:D)	1YVP; 2.20	synthetic	N/A

^a^Group 1 intron.

^b^A mammalian consensus sequence has been obtained.

^c^Synthetic.

### Structural analysis of modified base pairs in experimentally determined RNA structures

The set of PDB structures used in this work, updated to October 2013, contains 600 macromolecular structures solved by X-ray crystallography at a resolution of 3.5 Å or better that contain RNA molecules with posttranscriptional modifications [filtered by the ‘Has modified residues’ option in the wwPDB database (19)]. The modified RNA structure set was kept redundant with respect to RNA sequences, because even structures of the same RNA molecule can present different geometries for corresponding base pairs (see below). The 600 structures of modified RNAs were analyzed using the BPView tool (45), in order to identify the modified base pairs and classify their geometry. As a result of this analysis, we obtained 573 base pairs containing at least one modified base (Supplementary Table S1). Modifications of ribose or phosphate moieties were not considered here.

### Model building and QM calculations

The initial models for the QM calculations were built starting from the highest resolution crystal structures available. The PDB IDs and corresponding residue numbers used are listed in Table 1. For the unmodified counterparts, the modified residue was replaced by its corresponding unmodified one, by preserving the H-bonding pattern of the modified base pair. In the present calculations, ribose is not included, unless it participates in H-bonding interactions with the modified bases. Models of the bases are thus normally truncated at the C1′ atom of the ribose. When the ribose is included in the model, nucleosides are terminated by replacing the –CH_2–_5′OH and the -3′OH groups by a methyl group. This is a standard approach used previously (23,26,30,46,47). When a water molecule in the X-ray structure was observed to be mediating the H-bonds between the bases (see m^1^A:U tHW(w) below), it was also explicitly included in the model. A density functional theory approach, based on the hybrid B3LYP functional as implemented in the Gaussian 09 package (48,49), and the cc-pVTZ basis set (50), was used for all the geometry optimizations. Interaction energies were calculated on the B3LYP/cc-pVTZ optimized geometries at the second order Møller-Plesset level of theory, MP2 (51) using the more extended aug-cc-pVTZ basis set, in the framework of the Resolution of Identity approximation RIMP2 (52) method as implemented in Turbomole 6.1 package. The RMSD of the optimized geometry on the corresponding X-ray one were calculated on the corresponding heavy atoms after best superimposition. For unmodified versus modified geometries comparison, only atoms present in both the bases were used in the calculation. In case of pseudouracil, structurally (not chemically) correspondent atoms were superimposed. In this work, we calculated the interaction energy of the modified base pairs and of the corresponding unmodified pair, E_int_, as in Equation (1):

(1) \begin{equation*} E_{{\mathop{\rm int}} } = E_{{\rm BP}} - (E_{{\rm B}1} + E_{{\rm B}2} ) + {\rm BSSE}; \end{equation*}

where, *E*_BP_ is the electronic energy of the optimized base pair, and *E*_B1_ and *E*_B2_ are the electronic energy of the isolated and optimized geometry of the B1 and B2 bases forming the H-bonded base pair BP. All the interaction energies were corrected for basis set superposition error (BSSE) (53), using the counterpoise procedure.

To have an immediate and intuitive understanding of the impact of a specific modification, we introduce the modification energy, *E*_Mod_, defined as the energy difference between the interaction energy of the modified and of the corresponding natural base pairs, as shown in Equation (2).

(2) \begin{equation*} E_{{\rm Mod}} = E_{{\rm Int}} (\rm modified\;{\rm base}\;{\rm pair}) - E_{{\rm Int}} ({\rm natural}\;{\rm base}\;{\rm pair}). \end{equation*}

Within this definition, positive and negative *E*_Mod_ values indicate modifications that decrease or increase the stability of a specific base pair, respectively.

It should be noted that quantum mechanics calculations, such as those discussed in this work, localize minima on the potential energy surface of isolated systems at formally zero Kelvin (54). This implies that the calculated interaction energies cannot directly be compared to the experimental free energies of RNA folding or stem formation (50–53). In fact, in addition to the intrinsic stability of the base pair, as calculated in this work, the experimental values depend also on the specific environment, which means stacking interactions, interaction with the RNA ribose and phosphate, cations surrounding the RNA and solvent molecules.

## RESULTS

We collected all experimental RNA structures presenting modifications, to characterize the frequency, geometrical features and structural context of base pairs presenting at least one modified nucleobase. Our goal was: i) compiling a complete atlas of till now experimentally observed modified base pairs, and ii) characterizing them by advanced quantum mechanics calculations, especially focusing on the effect of the modification on the geometry and energetics of each base pair.

**Figure 2.**
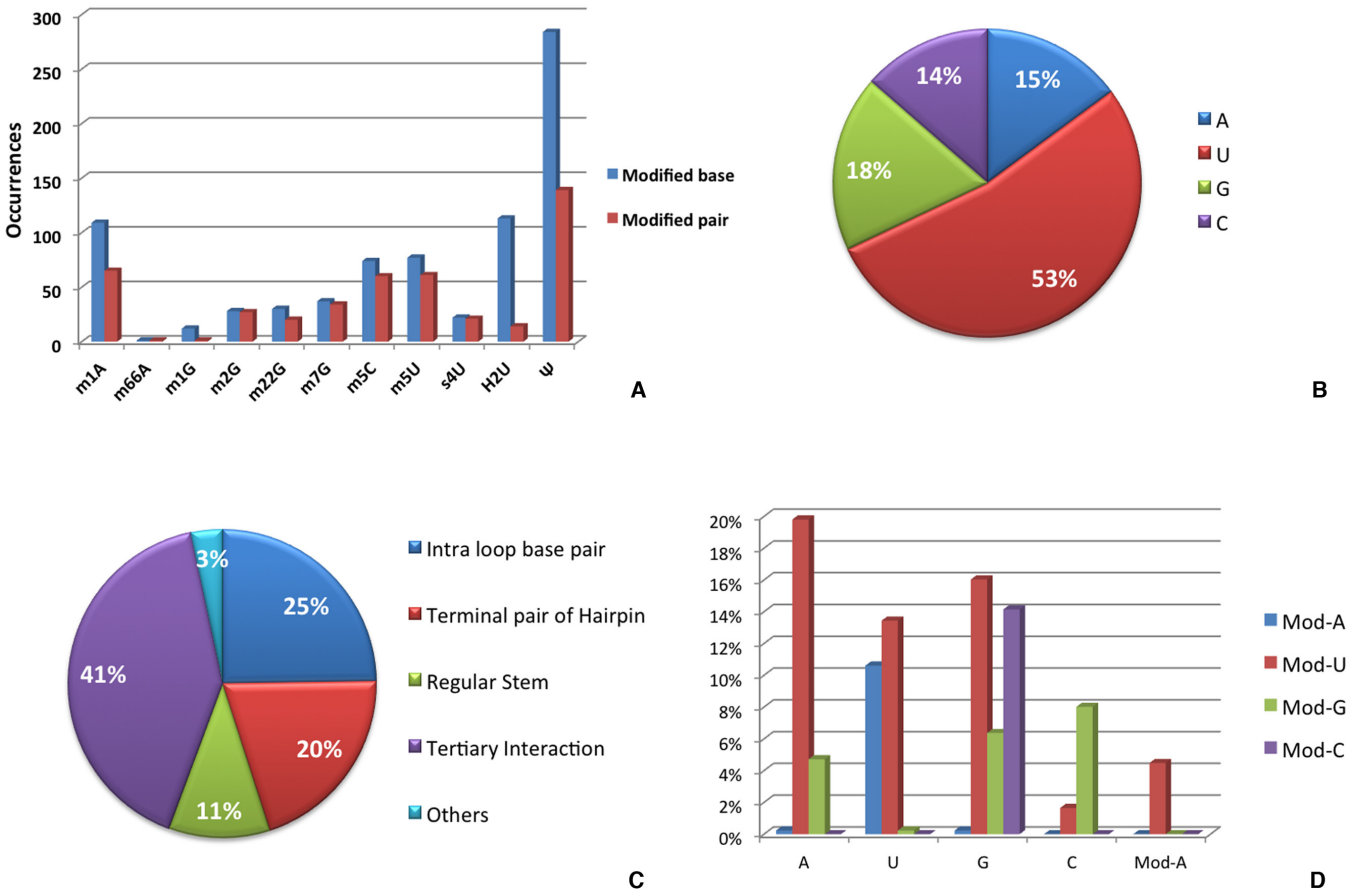
Statistical analysis of modified base pairs including natural nucleobase modifications. (**A**) For each modification, the total number of occurrences and the number of base pairs involving it are reported; (**B**) fraction of nucleobases that are modified and involved in base pairs, by parent nucleobase identity; (**C**) fraction of modified base pairs in different RNA structural motifs; (**D**) fraction of unmodified nucleobases H-bonding to modified nucleobases.

Remarkably, 49% of the total modified nucleobases (443 out of 906) in our structures collection were found to be involved in base pairing interactions. However, a great variability is observed when the propensity of each modification to be part of a base pair is recorded (Figure 2A). Indeed, while some modifications, such as for instance N2-methylguanine (m^2^G) or 4-thiouracil (s^4^U), are mostly involved in modified base pairs, others, such as dihydrouracil, only rarely participate in them. Remarkably, nine modifications covering roughly 10% of the total occurrences (119 over 906) were never found involved in base pairing interactions (see Supplementary Table S2). Two of these modifications (N7–4,5-cis-dihydroxy-1-cyclopentenyl-3-aminomethylguanine and 5-methoxycarbonylmethyl-2-thiouracil) are always located at the wobble position of the anticodon on tRNA molecules, while other five (2-methyladenine, 2-methylthio-N6-isopentenyladenine, N6-threonylcarbamoyladenine, 2-methylthio-N6-threonylcarbamoyladenine and wybutosine) occupy the position immediately 3′ to the anticodon. Hypermodified purines at this position are known to stabilize the tRNA-mRNA pairing on the ribosome, through stacking and additional interactions with ribosomal elements (41,55,56). As a result of our extensive search, a total of 573 H-bonded RNA base pairs involving at least one modified base were identified (see Supplementary Table S1 for a complete list). 424 of them include natural modifications of the four canonical bases. The most frequent naturally modified base moiety is pseudouracil found in about one third of the cases, 149. This is not surprising, as pseudouridine is well known to be an ubiquitous and abundant residue in RNA, counted as the fifth nucleotide (38). Hundreds of pseudouridylated sites were recently also found in mRNAs from yeast and human (57). Other 96 H-bonded bases involving a noncanonical nucleobase deriving from modification of uracil were recorded, making uracil the overall most frequently modified nucleobase in RNA, see Figure 2B. The remaining three nucleobases adenine, guanine and cytosine were found to be modified in 66, 82 and 60 pairs, respectively.

Upon classification of their base pairing geometry, an atlas of 36 unique ‘modified base pairs’, differing by the identity of H-bonded bases and/or geometry classification, has been compiled, 27 of them containing natural posttranscriptional modifications (with one base pair, m^1^A:m^5^U tHW simultaneously presenting two modified nucleobases) and 9 containing non-natural modifications (Table 1). The 27 ‘natural’ modified base pair types we classified exhibit a variety of different geometries, involving all the possible combinations of Watson–Crick (W), Hoogsteen (H) and sugar (S) edges and both the *cis* (c) and *trans* (t) glycosidic bond orientations, with the latter one being predominant, and are involved in a variety of structural motifs. Over 40% of them indeed participate in long-range tertiary interactions, while only 11% are located in regular stems (see Figure 2C). Furthermore, they are located in a variety of RNA molecules (see Table 1), including recently identified small non coding RNAs, although tRNA is not surprisingly the most represented molecule. The analysis of the identity of nucleobases involved in modified base pairs shows a distinct preference of each nucleobase for pairing with specific modified nucleobases (Figure 2D). For instance, guanine is found to give a significant number of H-bonded pairs with a modified U, G or C but is never found paired to a modified A. Adenine instead shows a clear preference for pairing to a modified U.

In the following, we will review the geometry of each modified pair and we will report their occurrences together with the structural contexts they have been found in. Then, we will report results of quantum mechanics calculations on representatives for each distinct base pair type to investigate their optimal geometry and energy.

### Occurrence and structural context

#### Base pairs involving modified adenine

**Figure 3.**
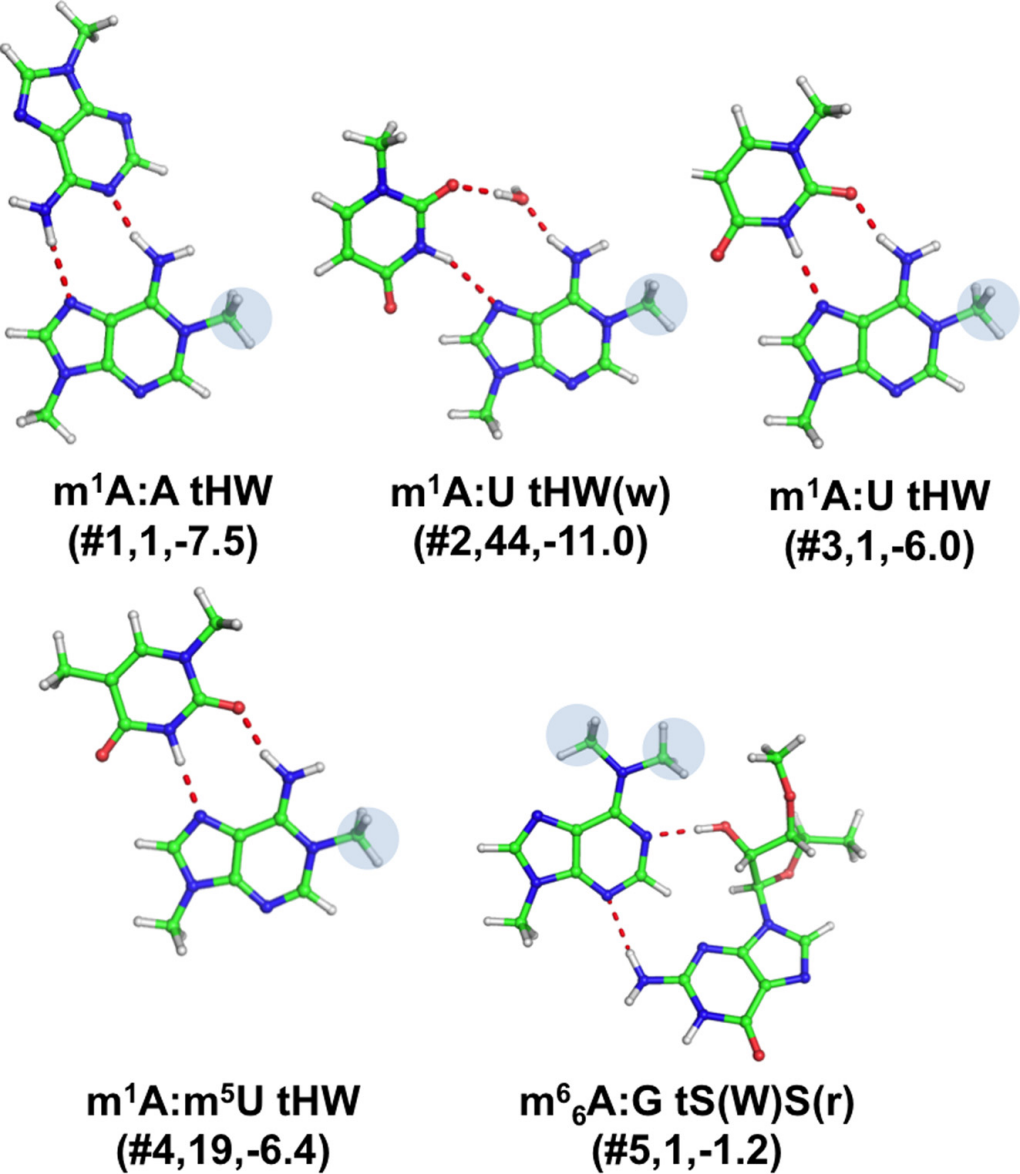
Stick representation of base pairs including a modified adenine. Under the base pair classification, the identifier of the modified base pair (see Table 1), preceded by a #, its number of occurrences and the *E*_mod_ values, in kcal/mol, are reported.

Two modifications were found for adenine, resulting in five distinct base pairing patterns (See Table 1, Figures 1 and 3).

##### 1-methyladenine (m^1^A)

Modification in the positively charged 1-methyladenine (m^1^A) only affects the Watson–Crick edge of the nucleobase, thus leaving both the Hoogsteen and sugar edges available for H-bonding interactions. The positively charged m^1^A is observed to participate in four distinct base pairing interactions (Figure 3). The first one is m^1^A:A tHW (#**1**, Table 1), where the Hoogsteen edge of m^1^A is involved in two H-bonds with the Watson–Crick edge of an adenine. Only one instance of this specific base pair was observed, in the T-loop of yeast tRNA(iMet), at positions 54–58. Importantly, the lack of the m^1^A modification has been shown to lead to an accelerated degradation of the tRNA molecule (58,59). Two different geometries were then observed for the m^1^A:U base pair (#**2,3**). Both of them are of the tHW type, i.e. involve the Hoogsteen edge of m^1^A and the Watson–Crick edge of U in a *trans* conformation, and constitute the ‘lone’ pair in a lonepair tri-loop motif (LPTL), a structural motif characterized by a single base-pair capped by a hairpin loop made of three nucleotides and usually involved in tertiary interactions with another section of the RNA. However, the first geometry presents a bridging water molecule between the two bases that is absent in the second one. 44 instances of the m^1^A:U tHW(w)(#**2**) geometry have been observed, where a structural water molecule is involved in H-bonding with N6(m^1^A) and O2(U), located in a LPTL motif of 23S rRNA from *H. marismortui*. A single instance of the simple m^1^A:U tHW(#**3**) pair has been instead observed at positions 54–58 of a tRNA, specifically of *E. coli* tRNA(Phe). The 54–58 pair is one of the nine tertiary interactions maintaining the fold of canonical tRNAs. It is known that modification of the T-loop region can influence the processing of the 3′ and 5′ termini, as well as the CCA-addition (60). Geometry and stability of the above pair in the gas phase were investigated in our previous study, within a comprehensive analysis of energetics of tRNA tertiary interactions (28). Similarly to other base pairs corresponding to tRNA tertiary interactions, the 54–58 pair has however been included in this study, for the sake of completeness. The fourth base pair involving m^1^A is m^1^A: m^5^U tHW(#**4**), which actually involves two modified nucleobases, i.e. m^1^A and 5-methyl uracil (m^5^U i.e. thymine). It presents the same geometry already discussed for m^1^A:U tHW, the only difference being in the modification of the uracil at the C5 position. 19 instances of the m^1^A: m^5^U tHW(#**4**) motif were detected at positions 54–58 in different tRNA molecules, including yeast tRNA(Phe).

##### N6-dimethyladenine (m^6^_6_A)

Dimethylation of A to m^6^_6_A affects both the Watson–Crick and Hoogsteen edges, thus leaving only the sugar edge with the same H-bonding potential as in unmodified A. A single instance has been observed for this base pair, corresponding to the m^6^_6_A:G tS(W)S(r) (#**5**) geometry (Figure 3), stabilized by N2-H(rG)-N3(m^6,6^A) and O2′-H(rG)-N1(m^6^_6_A) H-bonds, in the large ribosomal subunit from *H. marismortui*, and in particular between 23S rRNA and an aminoacyl-tRNA analogue bound to the A site (61). The ribose of the guanine has been included in the analysis, as it is involved in H-bonding interaction with N1(m^6^_6_A) in this base pair.

#### Base pairs involving modified guanine

**Figure 4.**
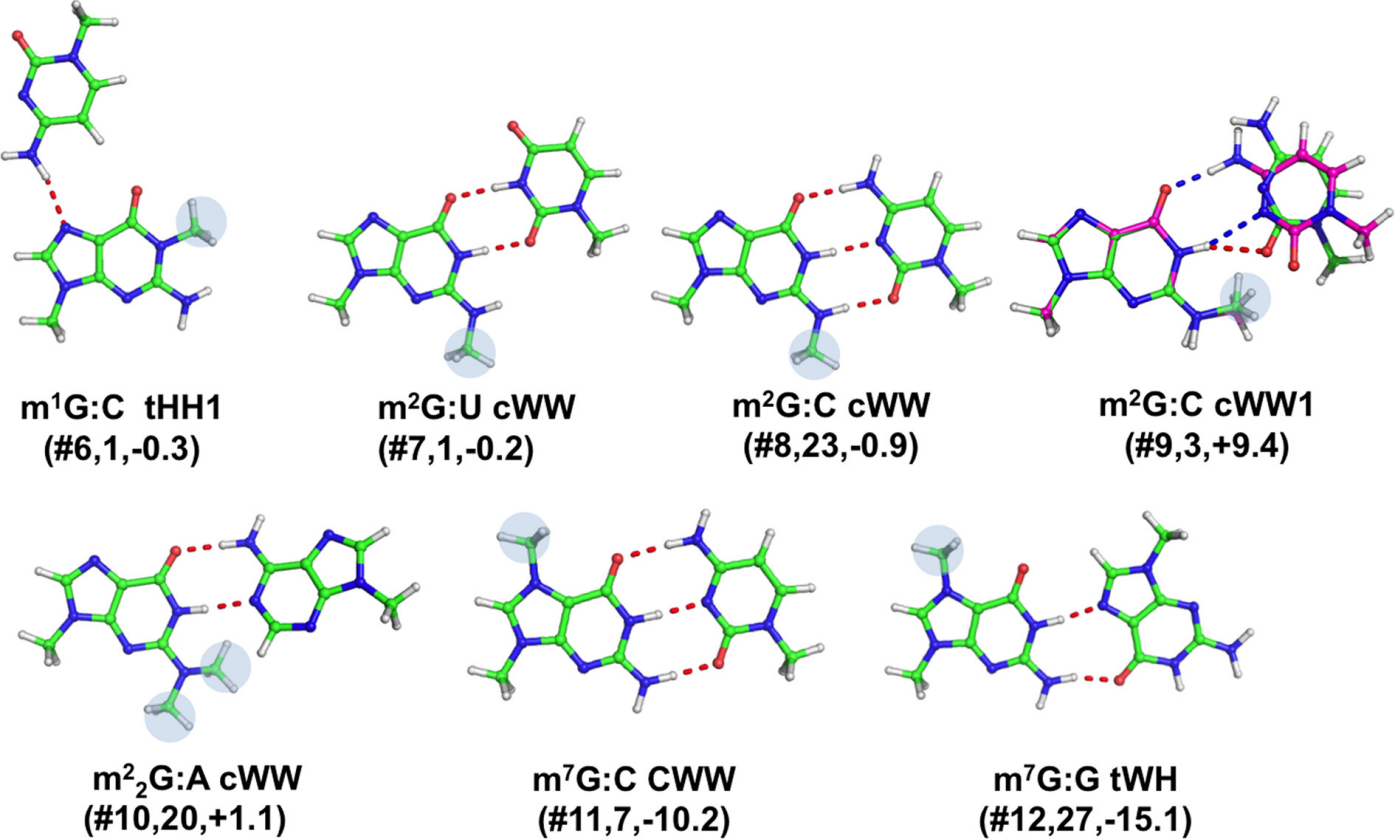
Stick representation of base pairs including a modified guanine. Under the base pair classification, the identifier of the modified pair (Table 1), preceded by a #, its number of occurrences and the *E*_Mod_ values, in kcal/mol, are reported. When the hydrogen bonds pattern is not conserved in the optimized geometry, a superimposition of the X-ray (in green, with H-bonds in red) and optimized geometry (in magenta, with H-bonds in blue) is shown.

Four modifications were found for guanine, resulting in seven distinct base pairing patterns (See Table 1, Figures 1 and 4).

##### 1-methylguanine (m^1^G)

In 1-methylguanine (m^1^G), the H-bonding potential at the Watson–Crick edge is affected by the modification. m^1^G is involved in one modified base pair, m^1^G:C tHH1(#**6**) pair, stabilized by a single strong H-bond between N4(C) and N7(m^1^G), for which only one instance was observed at positions 9–23 of yeast tRNA(iMet), as a part of a tertiary interaction in the tRNA D arm.

##### N2-methylguanine (m^2^G)

Single methylation at N2 may affect either the Watson–Crick or the sugar edge, depending on the orientation of the methyl group at the N2 position. m^2^G is involved in three different base pairs. The first one is m^2^G:U cWW(#**7**), for which a single instance was observed, as part of the acceptor stem of HIV-1 reverse-transcription primer tRNA(Lys,3). It is stabilized by the same H-bonds pattern of a ‘classical’ G:U cWW wobble base pair geometry. The second and third base pairs involving m^2^G can both be classified as m^2^G:C cWW(#**8,9**). However, whereas the former pair corresponds to a ‘regular’ G:C Watson–Crick *cis* geometry, stabilized by three H-bonds, in the latter pair the additional methyl group at N2 position of m^2^G is pointed toward the Watson–Crick edge, making the ‘classical’ three H-bonds Watson–Crick pairing sterically unfeasible. Instead, in the X-ray structure, only one H-bond, N1(m^2^G)-O2(C), is present. For the ‘regular’ m^2^G:C cWW base pair, a total of 23 instances were recorded, 16 of them in tRNA molecules, at the 10–25 positions, i.e. the terminal pair of the D-stem, and the remaining 7 instances in the regular helix-34 of 16S rRNA from *T. thermophilus*. 3 instances were instead observed for the m^2^G:C cWW1 base pair, again at positions 10–25 of tRNA molecules. It is worth reminding here that the 10–25 pair in tRNAs is usually part of a triplet, involving G45, which, from the variable loop, H-bonds to O6 of (m^2^)G10.

##### N2, N2-dimethylguanine (m^2^_2_G)

Modification of guanine to N2,N2-dimethylguanine (m^2^_2_G) partially affects both the Watson–Crick and sugar edges. m^2^_2_G is involved in one base pair, classified as m^2^_2_G:A cWW(#**10**), and characterized by two H-bonds. For this pair, 20 instances were recorded at positions 26–44 of tRNA molecules, where typically a purine-purine base pair causes a kink between the anticodon and D stems.

##### 7-methylguanine (m^7^G)

Modification of guanine to 7-methylguanine (m^7^G) introduces a positive charge on the nucleobase and affects the H-bonding potential only of its Hoogsteen edge. In the data set analyzed herein, m^7^G is involved in two base pairs. The first one is in fact a canonical m^7^G:C cWW(#**11**), for which 7 instances were recorded in helix 18 of 16S rRNA from *T. thermophilus*. The second one is a m^7^G:G tWH(#1**2**), for which 27 instances were recorded, at positions 22–46 of tRNAs, where it is part of the tertiary 13–22–46 triplet, joining the D-arm and the variable loop.

#### Base pairs involving modified cytosine

**Figure 5.**
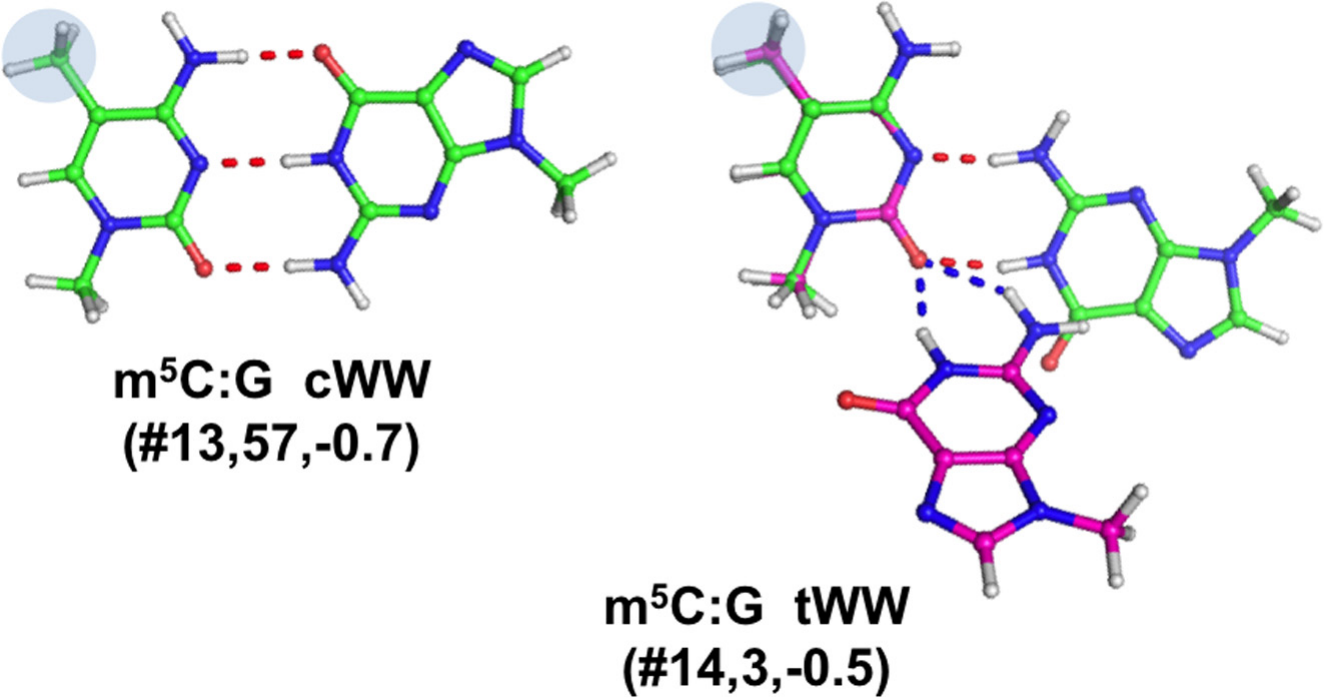
Stick representation of base pairs including a modified cytosine, the identifier of the modified pair (Table 1), preceded by a #, its number of occurrences and the *E*_Mod_ values, in kcal/mol, are reported. When the hydrogen bonds pattern is not conserved in the optimized geometry, a superimposition of the X-ray (in green, with H-bonds in red) and optimized geometry (in magenta, with H-bonds in blue) is shown.

We detected only one natural modification of the cytosine nucleobase, involved in two distinct base pairing patterns (see Table 1, Figures 1 and 5).

##### 5-methylcytosine (m^5^C)

Modification of cytosine to m^5^C leaves the Watson–Crick and sugar edges unaffected, while it alters the H-bonding potential of the Hoogsteen edge. In the data set analyzed herein, this modification is involved in two distinct types of base pairs. The first base pair is a canonical m^5^C:G cWW(#**13**), for which a total of 57 instances were found. Out of these 57 instances, 14 were observed in helix 44 of 16S rRNA from *T. thermophilus*, 15 instances at positions 40–30 of tRNA molecules, in the anticodon stem, 28 instances at position 49–65 of tRNA molecules, which is a terminal pair of the T-stem. The second base pair is a reversed (*trans*) Watson–Crick pair m^5^C:G tWW(#**14**). For this pair we detected 3 instances, at positions 48–15 of tRNAs, in which it is involved in a tertiary interaction that joins V-loop and D-stem.

#### Base pairs involving modified uracil

**Figure 6.**
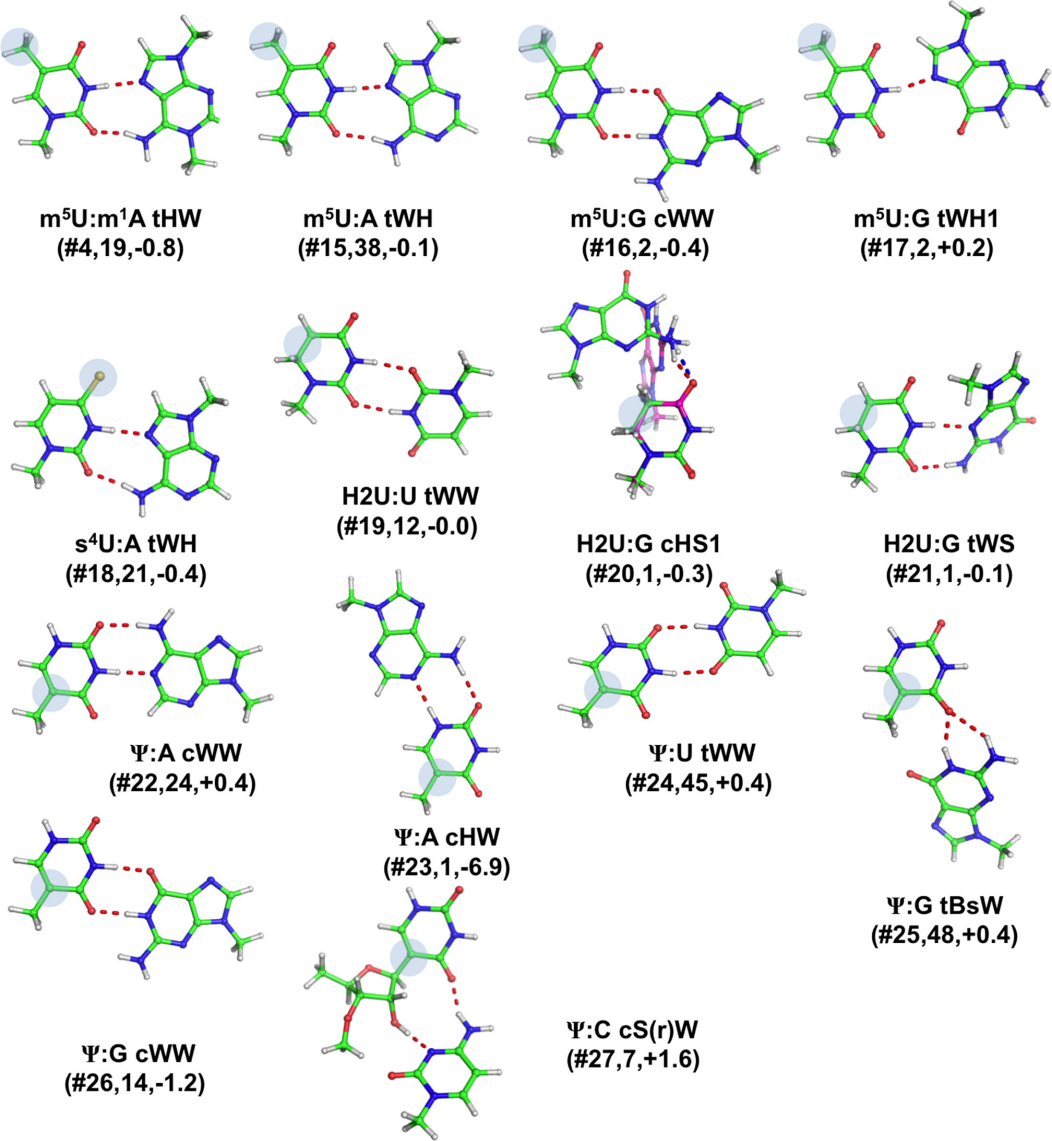
Stick representation of base pairs including a modified uracil. Under the base pair classification, the identifier of the modified pair (Table 1), preceded by a #, its number of occurrences and the E_Mod_ values, in kcal/mol, are reported. When the geometry significantly changes upon optimization, a superimposition of the X-ray (in green, with H-bonds in red) and optimized geometry (in magenta, with H-bonds in blue) is shown.

As anticipated above, most of the modified base pairs we found involve a modified uracil. Four distinct natural modifications were observed for uracil, forming fourteen distinct pairs (Table 1, Figures 1 and 6).

##### 5-methyluracil (m^5^U)

Methylation of uracil at position C5 impairs possible H-bonding interactions from the Hoogsteen edge, while leaving the Watson–Crick and sugar edges unaltered. We could detect four distinct modified base pairs involving m^5^U. The first one is m^5^U: m^1^A tWH(#**4**), and was discussed before, when presenting modified base pairs involving m^1^A. The second one corresponds to m^5^U:A tWH(#**15**), for which we recorded 38 instances at positions 54–58, i.e. a tertiary interaction within the T loop of tRNA molecules. The third one is a m^5^U:G tWH1(#**16**), characterized by a single H-bond, for which we found 2 instances, at the same location (positions 54–58) of tRNA molecules. Finally, the forth base pair is m^5^U:G cWW(#**17**) and also for it we could detect two instances, in a ribozyme (group I intron from *Azoarcus* sp.BH72) structure (62).

##### 4-thiouracil (s^4^U)

The second observed modification of U is the result of thiolation at C4 atom of uracil, resulting in 4-thiouracil (s^4^U), which affects the border between the Watson–Crick and the Hoogsten edges, while the sugar edge is unaffected. In the data set analyzed, this modification is involved in only one base pair, s^4^U:A tWH(#**18**), for which we recorded 21 instances, all in tRNA molecules, at the positions 8–14, which are actually part of the 8–14–21 tertiary interaction keeping together the tRNA acceptor stem and D arm.

##### Dihydrouracil (H2U)

The third modification of U corresponds to the reduction of uracil at the C5 and C6 positions, resulting in dihydrouridine (H2U), which is a non-planar nucleobase, as a consequence of the loss of the double bond between C5 and C6. The two additional hydrogen atoms are therefore located on the Hoogsten edge, while the Watson–Crick and sugar edges are virtually unaffected. For this modification, we could detect three distinct base pairs. 12 instances were observed for the H2U:U tWW(#**19**) pair, present at positions 16–59 (corresponding to canonical positions 17–59) of tRNA molecules, an additional interaction between the D and T loops, besides the known ‘canonical’ ones (28,30). One instance was observed at that position for H2U:G cHS1(#**20**), characterized by a single H-bond, which represents a dinucleotide platform interaction (i.e. two consecutive residues H-bonded to each other (63,64)) in the D loop of tRNA(Asp). Similarly, a single instance was recorded for the H2U:G tWS(#**21**) base pair, as part of the *T. thermophilus* tRNA(Ser) D loop. Therefore, all the H2U occurrences we found are located within the D loop of tRNA molecules, a region also known to be involved in interaction with aminoacyl tRNA synthetases.

##### Pseudouracil (Ψ)

Pseudouracil, Ψ, is connected to the sugar backbone not through the pyrimidine N1 atom but through C5, as a result of an isomerization (38). In Ψ, an additional polar hydrogen bond donor N1-H is present on the Hoogsteen edge, at the equivalent site of uracil C5-H, while the Watson–Crick and sugar edges are unaffected compared to unmodified uracil. Our database search probed six distinct base pairs involving Ψ, with all the canonical A/U/G/C bases. The first base pair is Ψ:A cWW(#**22**), where Ψ forms a Watson–Crick pair with an adenine. For this base pair we recorded 24 occurrences, 13 of which represented the terminal pair in the hairpin loop, 3 in the anti-codon stem and 2 in the acceptor stem of different tRNAs, while 3 instances correspond to codon(mRNA)-anticodon(tRNA) interactions, and the remaining 3 ones were observed in U2 small nuclear (sn)RNAs. The second pair is a Ψ:A cHW(#**23**), where Ψ is rotated by 180° compared to the previous pairing around the C5-C1′ bond (it is in the ‘syn’ conformation, thus utilizing its Hoogsteen edge (65)), yielding however a similar H-bonding pattern to that of the Watson–Crick edge. This base pair we observed only once, in yeast tRNA(Phe), at positions 39–31, i.e. the last, usually non canonical, pairing before the anticodon loop. It is interesting that, of the three tRNA molecules present in the corresponding X-ray structure (PDB ID: 1TTT (66)) only one (chain D) presents this specific geometry, while the other two pairs present a Ψ:A cWW geometry instead. We observed 45 instances of the Ψ:U tWW(#**24**) base pair, all in 23S rRNA from *H. marismortui* (PDB numbering: Ψ2621:U1838). Interestingly, U1838 is present in the 23S rRNA domain IV, while Ψ2621 is present as a part of a junction in domain V. Thus, this is a tertiary interaction between two different domains that may be important for stabilization of the ribosome structure.

Another base pair involving Ψ is Ψ:G tBsW(#**25**), which is a bifurcated H-bonding interaction involving the sugar edge of Ψ and the Watson–Crick edge of G, characterized by the N1(G)-O4(Ψ) and N2(G)-O4(G) H-bonds. We found 48 instances of this base pair, in tRNA structures, at positions corresponding to canonical 55–18, i.e. one of the key tertiary pairs keeping together the D and T loops, at the elbow of the ‘L-shaped’ structure. In addition, a conserved H-bonding interaction was observed between N3(Ψ55) and O2P(A58), that was however not explicitly simulated in our calculations, as we did not consider H-bonding with the ribose-phosphate backbone. Further, 14 instances of the Ψ:G cWW(#**26**) base pair for which, 9 instances were found at 13–21 position, last pair of D-loop in tRNAs, 3 instances were recorded at the terminal pair of T hairpin stem, one instance in the regular stem of anticodon region, and another one instance was found in a U2 small nuclear (sn)RNA, analogously to the Ψ:A cWW pase pair discussed above. Actually, the two U2 snRNA structures only differ for the identity of residue 20 (67). When a G20 is there, the Ψ pairs with it, with a cWW geometry, while residue 21 (A21) protrudes from the double helix toward the solvent; whereas when a A20 is there, Ψ pairs with A21 and it is A20 to protrude from the double helix. Finally, we could observe 7 instances of the Ψ:C cS(r)W(#**27**) interaction, which is part of an internal loop of helix 18 in 16s rRNA from *T. thermophilus*.

#### Base pairs involving non-natural modifications

**Figure 7.**
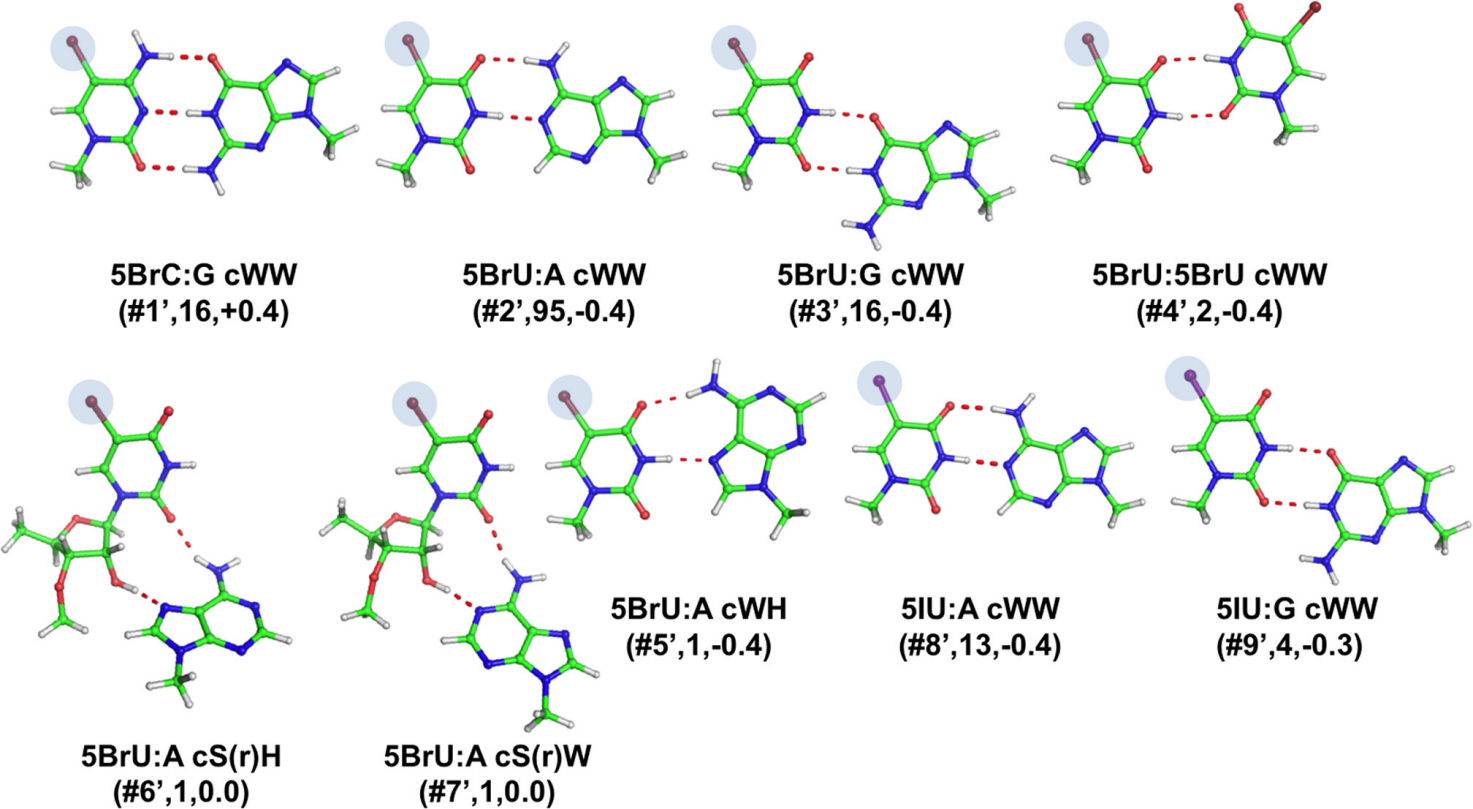
Stick representation of base pairs including non-natural modifications. Under the base pair classification, the identifier of the modified pair, preceded by a #, its number of occurrences and the E_Mod_ values, in kcal/mol, are reported.

Three non-natural modifications were observed in the data set analyzed, all corresponding to halogenation (bromination or iodination) of pyrimidine bases at C5, introduced into RNA molecules to help in solving the X-ray phase problem. (See Materials and Methods for the adopted nomenclature). All these modifications affect the Hoogsteen edge of the corresponding nucleobases, leaving the Watson–Crick and sugar edges unaffected. They are involved in 9 different types of base pairs (Table 1, Figures 1 and 7).

##### 5-Bromocytosine (5BrC) and 5-Bromouracil (5BrU)

In the data set analyzed, 5BrC is involved only in 5BrC:G cWW(#**1’**) pairs. We observed 16 instances of this base pair, 3 of which were in helix 6 of synthetic human SRP (signal recognition particle) RNA, 12 in engineered brominated RNA, and one in the sarcin/ricin loop in synthetic 28S rRNA from rat. 5BrU is observed to be involved in six different modified base pairs. The 5BrU:A cWW(#**2’**), 5BrU:G cWW (#**3’**) and 5BrU:5BrU cWW(#**4’**) base pairs (95, 16, and 2 instances, respectively) all share a Watson Crick geometry and were found in stem regions of various synthetic molecules. In the 5BrU:A cWH(#**5’**) base pair, the Watson–Crick edge of 5BrU hydrogen-bonds to the Hoogsten edge of an adenine. The remaining two base pairs, 5BrU:A with a cS(r)H(#**6’,7’**) and cW(r)S, both present an H-bond between O2(5BrU) and N6(A). In addition, the ribose O2′ atom of 5BrU hydrogen-bonds to N7(A) and N1(A), respectively. One only instance for each of the last three modified pairs was found, in HCV RNA and a synthetic RNA aptamer.

##### 5-Iodouracil (5IU)

In the data set analyzed, the 5IU modification was found in two modified base pairs, both Watson–Crick: 5IU:A cWW(#**8’**) and 5IU:G cWW(#**9’**). 13 and 4 occurrences of such pairs were found, all in synthetic molecules, some of them reproducing fragments of viral genomes, signal recognition particle RNA, RNA aptamers or snoRNA.

### Geometry and energetics

Optimal geometries and interaction energies have been calculated by quantum mechanics for representatives of the 36 modified base pair combinations we classified (Supplementary Tables S3 and 2). To investigate the effect of the modifications on the base pairs, we also compared the geometry and interaction energy of the modified base pairs with those of the corresponding unmodified ones. In the following, the main findings are reported, while details on geometry and energetics of each base pair are given in the Supplementary Information.

#### Geometric comparison of experimental versus optimized base pairs

Most of the X-ray geometries were maintained after optimization in the gas phase, indicating that they are also stable as isolated base pairs, independently from the structural context. Most differences in the H-bond distances between the optimized and experimental pairs are within 0.26 Å (Supplementary Table S3), which is in the expected range for this kind of calculations (22,25,28–30,46,47).

The H-bonding pattern was not maintained in only two cases, m^2^G:C cWW1(#**9**) and m^5^C:G tWW(#**14**). The m^5^C:G tWW pair is analogous to the well-known and widely characterized case of C:G tWW, shown to not be stable as an isolated base pair (28,68) and to be possibly stabilized by additional factors in the context of RNA structures (30,31). Due to repulsive amino-amino and carbonyl-carbonyl contacts, during the gas phase optimization the base pair indeed moves to a bifurcated H-bonding pattern, involving the central section of the G Watson–Crick face and the C carbonyl group adjacent to the C1′, which is classifiable as a G–C tWBs. This severe geometric rearrangement leads to an RMSD of 1.28 Å for the superposition of the optimized versus the experimentally determined structure (Supplementary Table S3). For m^2^G:C cWW1, a more stable Watson–Crick like arrangement is reached in the optimized structure, which is not observed in any of the three experimental occurrences available for this base pair type, although no impediment to it seems to exist. Furthermore, the base pair, which is propeller-twisted in the experimentally observed structure, converges to a planar geometry after optimization. The optimized geometry thus significantly deviates from the experimentally determined structure with an RMSD of 0.75 Å.

Only other three base pairs, although maintaining the H-bonding pattern upon optimization, undergo a conformational rearrangement resulting in RMSD values for superimposition of the experimental and optimized structures above 0.50 Å. Two of them, m^5^U:G tWH1(#**16**) and H2U:G cHS1(#**20**), are characterized by a single H-bond and feature a significant rearrangement of the bases around the axis of the single H-bond. In particular, the m^5^U:G tWH1 base pair, undergoes an opening of its single H-bond, which is elongated by 0.49 Å (the RMSD for superimposition being 0.54 Å). The elongation in the N3(m^5^U)-N7(G) distance is probably consequence of the repulsion between the negatively charged O2(m^5^U) and O6(G) atoms, only 2.55 Å apart in the experimental structure. As for the H2U:G cHS1 pair, which assumes experimentally a planar geometry, after optimization it loses planarity with the two bases becoming almost perpendicular, while the single N2(G)-O4(H2U) H-bond is elongated by 0.47 Å. Such rearrangement also implies a shortening of the distance between the two C1′ atoms, from 6.3 to 5.3 Å. This value is not realistic for two consecutive nucleobases, (this is indeed a dinucleotide platform), therefore we decided to simulate the entire two nucleotides, with their ribose-phosphate backbone. As a result, we found a C1′-C1′ distance of 7.16 Å, although a similar elongation in the N2(G)-O4(H2U) H-bond, by 0.54 Å, and an optimized geometry substantially propeller twisted was observed.

The remaining base pair undergoing a significant geometric rearrangement upon optimization is m^2^_2_G:A cWW(#**10**). The optimized geometry of m^2^_2_G:A cWW is more propeller-twisted than the experimental one, to avoid the steric repulsion between the methyl groups on N2 of m^2^_2_G and the hydrogen on the C2 atom of the adenine. The RMSD value for the experimental versus optimized geometry is 0.51 Å.

#### Geometric comparison of optimized modified versus optimized unmodified base pairs

We also compared the geometries of optimized modified pairs with those of the corresponding optimized unmodified pairs. Most differences in the H-bond distances are within 0.27 Å, whereas the RMSD values for best superimposition are within 0.22 Å (Supplementary Table S3). The only base pair largely deviating from its optimized unmodified counterpart is H2U:G cHS1(#**20**) (RMSD of 1.27 Å). However this is a base pair maintained by one only H-bond, and a significant rearrangement was also observed between the experimental and optimized geometries. Moderate geometric rearrangements were observed in other four cases: m^1^A:A tHW(#**1**), m^2^G:C cWW1(#**9**), m^2^_2_G:A cWW(#**10**), Ψ:A cHW(#**23**) (RMSD values in the range 0.36–0.55 Å). These findings indicate that modifications do not usually have a dramatic impact on the geometry of the base pairs they participate in, if the modification is distal from the edge involved in the base pairing.

#### Interaction Energies of Modified base pairs

Interaction energies are not surprisingly quite variable, as they are the result of multiple factors, such as the base pair geometry, the nucleobase identity, and the type of modification itself (see Table 2). The lowest E_Int_ value, −8.6 kcal/mol, was recorded for H2U:G tHS1(#**20**), characterized by a single H-bond. The highest E_Int_ value of −37.2 kcal/mol was instead obtained for the m^7^G:C cWW(#**11**) pair, characterized by a regular Watson–Crick geometry with three H-bonds, and enforced by a positive charge on the G pair. As a general trend, modified pairs with the modification introducing a positive charge (m^1^A and m^7^G) possess the highest interaction energies, ranging from −19.7 to −37.2 kcal/mol. Not surprisingly, the base pairs stabilized by a single strong H-bond, such as H2U:G cHS1(#**20**), m^5^U:G tWH1(#**16**) and m^1^G:C tHH1(#**6**), possess E_Int_ as low as −8.6, −9.8 and −10.7 kcal/mol, respectively.

**Table 2.** Interaction energies, in kcal/mol, of the modified base pairs, of the corresponding unmodified base pair, and of the modification energy E_mod_

Parent Base	#	Base Pair	E (modified)	E (unmodified)	E_mod_
Adenine	1	m^1^A:A tHW	−19.74	−12.24	−7.50
	2	m^1^A:U tHW(w)	−31.13	−20.11	−11.02
	3	m^1^A:U tHW	−21.75	−15.72	−6.03
	4	m^1^A: m^5^U tHW	−22.54	−15.79	−6.35
	5	m^6^_6_A:G tS(W)S(r)	−18.46	−17.24	−1.22
Guanine	6	m^1^G:C tHH1	−10.67	−10.38	−0.29
	7	m^2^G:U cWW	−15.77	−15.59	−0.18
	8	m^2^G:C cWW	−27.90	−27.02	−0.88
	9	m^2^G:C cWW1	−17.60	−27.02	9.42
	10	m^2^_2_G:A cWW	−16.09	−17.19	1.10
	11	m^7^G:C cWW	−37.17	−27.02	−10.15
	12	m^7^G:G tWH	−34.55	−19.43	−15.12
Cytosine	13	m^5^C:G cWW	−27.68	−27.02	−0.66
	14	m^5^C:G tWW	−17.96	−17.49	−0.47
Uracil	4	m^5^U: m^1^A tWH	−22.54	−21.74	−0.80
	15	m^5^U:A tWH	−15.80	−15.72	−0.08
	16	m^5^U:G cWW	−16.00	−15.59	−0.41
	17	m^5^U:G tWH1	−9.83	−10.08	0.25
	18	s^4^U:A tWH	−16.11	−15.72	−0.39
	19	H2U:U tWW	−11.86	−11.84	−0.02
	20	H2U:G cHS1	−8.55	−8.29	−0.26
	21	H2U:G tWS	−13.72	−13.66	−0.06
	22	Ψ:A cWW	−14.54	−14.93	0.39
	23	Ψ:A cHW	−16.00	−9.09	−6.91
	24	Ψ:U tWW	−12.35	−12.77	0.42
	25	Ψ:G tBsW	−12.85	−13.23	0.38
	26	Ψ: G cWW	−16.82	−15.59	−1.23
	27	Ψ:C cS(r)W	−19.44	−21.03	1.59
Non-natural	1’	5BrC:G cWW	−26.59	−27.02	0.43
	2’	5BrU:A cWW	−15.35	−14.93	−0.42
	3’	5BrU:G cWW	−15.96	−15.59	−0.37
	4’	5BrU:5BrU cWW	−12.63	−12.19	−0.44
	5’	5BrU:A cWH	−16.41	−15.98	−0.43
	6’	5BrU:A cS(r)H	−17.31	−17.27	−0.04
	7’	5BrU:A cW(r)S	−17.89	−17.93	0.04
	8’	5IU:A cWW	−15.35	−14.93	−0.42
	9’	5IU:G cWW	−15.91	−15.59	−0.32a

**Figure 8.**
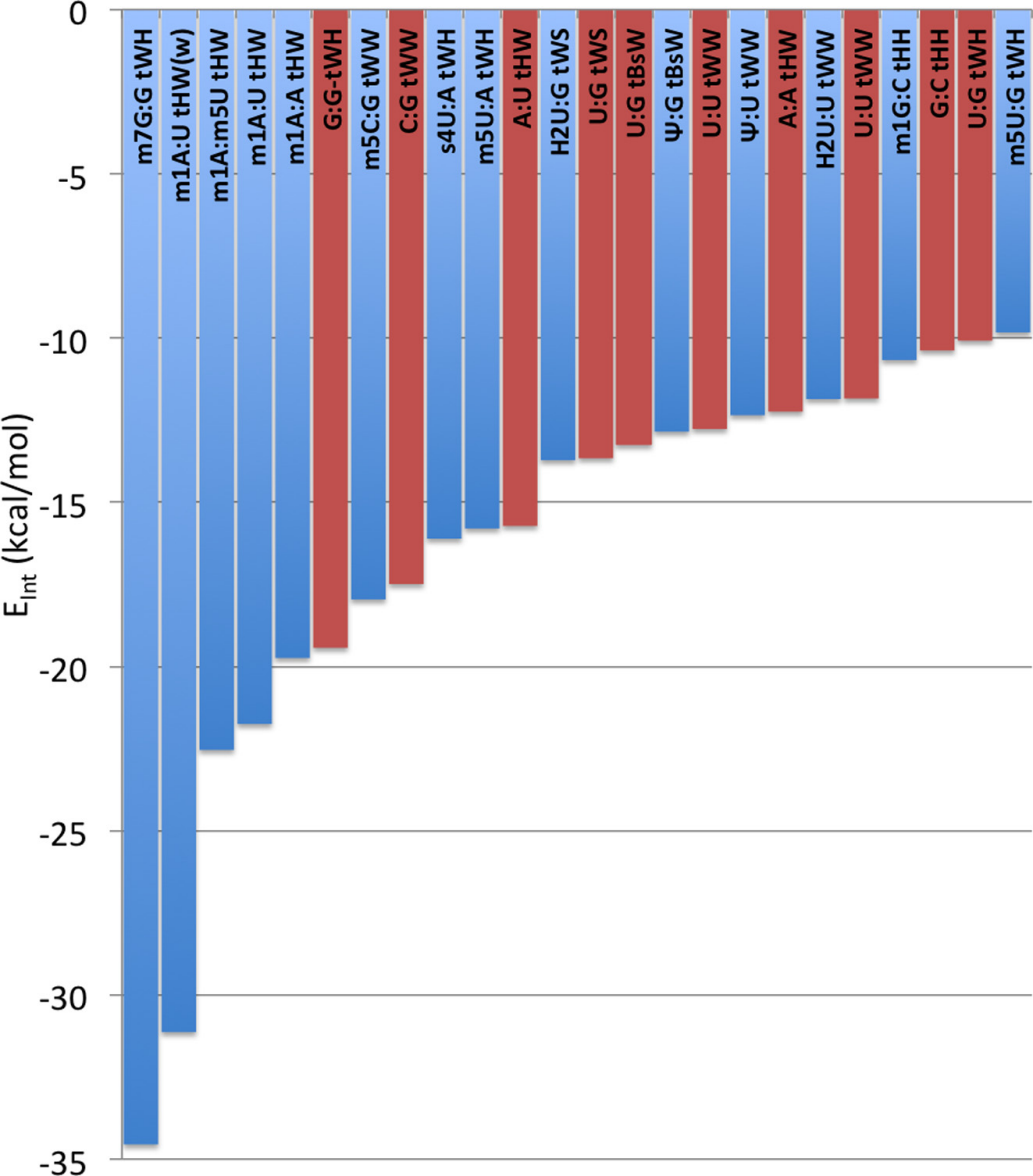
Trend in the interaction energies, E_Int_, in kcal/mol, of base pairs involved in tertiary interactions and containing at least one modified base (blue columns). The red columns report the E_Int_ values calculated for the corresponding unmodified base pairs.

To have an overall view of the impact of the modifications in tuning the pairs interaction energy, the E_Int_ of modified pairs was plotted together with the E_Int_ of the corresponding non-modified pairs, see Figure 8. Analysis is focused on pairs involved in tertiary interactions, since they represent the highest fraction of structural motifs containing modified pairs, see Figure 2C. Visual inspection of Figure 8 clearly indicates that modifications expand the range and finely tune the interaction energy values, allowing the geometry of a specific non canonical interaction to be maintained, with a modified stability. For instance, modifications introducing a positive charge, such m^1^A and m^7^G, enable the m^1^A:A tHW base pair to reach a stability comparable to that of the non-modified G:G tHW pair, or the m^7^G:G tWH pair to reach a stability comparable to that of the strongest canonical G:C cWW pair (E_Int_ = −27.0 kcal/mol, see Table 2).

#### Interaction energies comparison between modified and unmodified base pairs

To investigate the stabilizing/destabilizing effect of modifications on the corresponding base pairs, we calculated the modification energy, E_Mod_, that is the difference between the interaction energy of the modified and unmodified base pair (see Materials and Methods). E_Mod_ is defined so that a negative sign means that the modification stabilizes the base pair and *vice versa*. The calculated *E*_Mod_ values, reported in Table 2 and Figures 3–7, range from −15.1 kcal/mol, in m^7^G:G tWH(#**12**), with the modified base pair strongly stabilized by the positive charge introduced by the m^7^G modification, to 9.4 kcal/mol in m^2^G:C cWW1(#**9**), with the modified base pair presenting one H-bond less compared to the unmodified pair, as a consequence of the m^2^G modification. Nevertheless, in most of the cases the impact of the modification on the base pair stability is moderate, lying within 2 kcal/mol. As a general trend, methylations that introduce a positive charge on the base pairs are highly stabilizing. Conversely, methylations that introduce no charge on base pairs are marginally stabilizing. Similarly, hydrogenation and thiolation of the bases results in marginal increase in the stability of the base pairs. It is interesting to point out that, when focusing on the H-bonded bases, the Ψ modification seems rather to have a destabilizing than a stabilizing effect (see Conclusions).

To rationalize whether the stabilizing effect of methyl groups not introducing charges is due to inductive effects (through the σ-bonds skeleton of G/C/U), or to additional stabilizing dispersion interactions upon methylation, we compared the *E*_Mod_ values obtained by B3LYP, the approach used in the geometry optimizations, with values obtained by the B3LYP-D3 method, which includes an explicit term to account for dispersion interactions (69), for some exemplary cases. The selected test cases were: the m^2^G:U cWW (#7), m^2^G:C cWW (#8), m^5^C:G cWW (#13) and m^5^U:G cWW (#16) base pairs. The slight difference in the *E*_Mod_ calculated with the two methods, −0.1 for m^2^G:U cWW, m^5^C:G cWW and m^5^U:G cWW and −0.2 kcal/mol for m^2^G:C cWW, indicates that dispersion interactions contribute to a minor extent to the stability of the modified base pairs. Similar decomposition of the E_Int_ of the above base pairs between the Hartree–Fock and MP2 contribution terms, normally associated to the H-bond and to the dispersion interaction terms, also supports this conclusion.

#### Geometry and interaction energy of base pairs involving non-natural modifications

As for the 9 base pairs presenting non-natural modifications, they are mostly found in regular stems. They are also stable as isolated base pairs and their optimal geometries are highly similar to the experimental ones, but for the two of them having a ribose directly involved in H-bonds. The interaction energies, in most of the cases we investigated, show a small stabilizing effect, whose entity is comparable or higher than that of most of the neutral natural modifications we investigated, ranging between −0.44 and −0.04 kcal/mol, with the exception of 5BrU:A cW(r)S (#**7’**) and 5BrC:G cWW (#**1’**) with E_Int_ of +0.04 +0.43 kcal/mol. To test if the more polarizable halides could result in a greater contribution of dispersion interactions to *E*_Mod_, relative to the case of methyl modified base pairs, we compared the *E*_Mod_ calculated with the B3LYP and the B3LYP-D3 methods (the latter specifically tuned to include dispersion interactions) for an exemplary case, specifically for the 5BrU:G cWW (#**3’**) base pair. Also in this case we found that dispersion interactions contribute to a minor extent to the stability of the modified base pair, since the B3LYP-D3 *E*_Mod_ is less than −0.1 kcal/mol lower than *E*_Mod_ calculated at the B3LYP level.

## DISCUSSION AND CONCLUSIONS

As we have shown here, about half of natural posttranscriptional modifications in experimental structures of RNA molecules are involved in base pairs. This suggests that modifications can also be programmed by nature for the effect they have on the corresponding base pairs. We classified 27 distinct types of base pairs in RNA structures, characterized by the presence of naturally modified nucleobases (other 9 base pair types presented non-natural modifications). Naturally modified base pairs were particularly common in tRNAs, but were also found in ribosomal RNAs, ribozymes, snRNAs, and in various synthetic constructs. Eleven different natural modifications were included in our analysis, comprising neutral and positively charged, single and double methylated, thiolated, reduced and isomerized nucleobases. The geometries of these base pairs were very variable and involved all the possible pairing edges, Watson–Crick, sugar and Hoogsteen. A classical Watson–Crick pairing was only observed for 8 types of base pairs. Remarkably, 15 base pair types out of the 27 naturally modified ones have a *trans* orientation, i.e. opposite to the *cis* arrangement of the base pairs in the ‘canonical’ (antiparallel-stranded) double helix. These base pairs were indeed observed to be located in a variety of tertiary motifs, such as pairs and triplets corresponding to tRNA tertiary interactions, the single base pair in the lonepair tri-loop motif corresponding to tRNA T-loop and 25/26 junction in domain II of 23S rRNA, as well as mediating the interaction between the 23S domains IV and V and between 23S rRNA itself and a tRNA molecule on the ribosome from *H. marismortui*.

We also investigated the optimal geometry and energetics of representative of all the modified base pair types we classified, finding that most of them are also stable as isolated interactions in the gas phase. Further, we studied the effect of each modification on the geometry and energetics of the corresponding base pairs. General conclusions on the stabilizing/destabilizing effect of the different modifications are given below.

- 9 out of the 10 examined base pairs, which present modifications that introduce additional methyl groups, not engaged in repulsive steric interaction with the other base and not introducing a positive charge (namely m^6^_6_A, m^1^G, m^2^G, m^5^C and m^5^U), are slightly stabilized by the modification, with an *E*_Mod_ within −1.2 kcal/mol.
  Comparison of the B3LYP and B3LYP-D3 energies indicated that dispersion interactions contribute to a minor extent to the modified base pair stability, thus suggesting that the main driving force is in the H-bonding term. The ability of methylated bases to form stronger H-bonds can be related to the inductive effect of the added electron-donating methyl group, which reinforces the H-bond acceptor capability of the base pair. For example, the added methyl on m^5^U increases slightly, by −0.01e, the negative charge on the O2 and O4 atoms. The only case where methylation reduces the stability of the base pair, by 0.2 kcal/mol only, is for the m^5^U:G tWH1(#**16**) base pair. However, in this case the base pair presents a single H-bond, and m^5^U is engaged as a H-bond donor, while the added methyl enhances the H-bond accepting capability of the base. In this context, the slightly stabilizing effect of thiolate modification in the s^4^U:A can be rationalized considering that the H3 atom is slightly more acidic, by +0.01e, in s^4^U relative to U.
- Modifications introducing steric clashes with the interacting base, like the G to m^2^_2_G modification in m^2^_2_G:A cWW(#**10**), destabilize the corresponding base pairs. This is in line with a current view that modifications can stabilize functional RNA structures either by specifically contributing stability to a secondary or tertiary interaction (31,70–72), or by preventing certain pairings (usually Watson–Crick) that would otherwise lead to non-functional 3D structures (73–77). Often this impediment of an alternative pairing is due to a large steric hindrance on the modified base pair affecting its pairing potential. For instance, specifically m^2^_2_G at position 26 has been proposed to prevent potential misfolding of human tRNA(Asn) by preventing G26 from forming a Watson–Crick pair with C11 (73).
- Modifications that introduce a positive charge, like m^1^A or m^7^G, strongly stabilize the corresponding base pairs, with *E*_Mod_ in the −6.0 to −15.1 kcal/mol range in the five examined cases. This includes the case of m^1^A:A tHW(#**1**), in the T-loop of yeast initiator tRNA, where the m^1^A modification at position 58 is known to protect the RNA molecule from degradation (58,59). The stabilizing effect of positively charged modified nucleobases on H-bonding had already been reported by us (28,29,31), and mainly derives from improved electrostatic interaction between electron density on the unmodified neutral base with the positive charge on the modified base.
- Pseudouridine is usually reported to improve the RNA stability (37,78–81), by favoring a 3′-endo conformation of the ribose, which enhances the local stacking, and by a water-mediated H-bond between N1 (its additional H-bond donor) and the RNA backbone (82,83), which rigidifies the base itself and the backbone upstream (although a possible role as a conformational switch has also been proposed for it, based on the low energetic barrier for thesyn/anti transition (38,84). Still, the effect of this modification on base pairing interactions is also of interest. As a result of our study, we can say that it is easily rationalized considering that structurally similar base pair geometries (e.g the U:A and Ψ:A cWW) require that the H-bond involving the N1(A) donor is engaged with the O2 and the O4 H-bond acceptors, in U and Ψ, respectively. This change in the H-bond acceptor results in a less stable Ψ:A cWW base pair, since the O2 atom, with an atomic charge of −0.71e in U, is a better H-bond acceptor than the O4 atom, with an atomic charge of −0.68e. Similar reasoning explains the effect of the modification in all the other investigated base pairs involving Ψ, but for the Ψ:C cS(r)W(#**27**) and the Ψ:G tBsW(#**25**) pairs, as the first incorporates the ribose and the second presents a bifurcated H-bond only, thus making the analysis more complex. In the Ψ:G cWW(#**26**) base pair (here O4 of U is replaced by O2 in the modified base pair), the modified base pair is engaged in the H-bonding through the better H-bond acceptor O4, which immediately explains its higher stability. These base concepts can also be applied to explain the decreased stability of Ψ:U tWW(#**24**). The Ψ:A cHW pair represents instead a special case, as substituting the Ψ with an unmodified U in the same orientation means losing one H-bonding donor, N1, which is substituted by C5. The H-bonding acceptor O2 is instead substituted by an ‘equivalent’ O4. Therefore, energy optimization of U:A cHW results in an opening of the base pair from the minor groove with a remarkable elongation of the C5(U)-N1(A) distance (it was N1-N1 in Ψ), by 0.75 Å. The Ψ:A cHW pair, with one more H-bond than its unmodified counterpart, is clearly more stable, with an *E*_Mod_ of −6.9 kcal/mol. It is interesting that, when focusing on the H-bonded bases, the Ψ modification seems rather to have a destabilizing than a stabilizing effect.
- It should be noted that the dihydrouridine modification results in increased RNA flexibility, also by destabilization of the C3′-endo ribose puckering, associated with base stacked and ordered A-type helical RNA (40). Such factors, concerning the effect of the modification on the RNA backbone, have not been considered here. However, as this modification results in removing the aromaticity of the parent base, it is of great interest to investigate which is its effect on the base pairing potential. As a first result, we observed that the H2U modification, with the hydrogenation of the C5-C6 bond, results in a deformation from planarity of the base, with the N1-C6-C5-C4 and N1-C2-N3-C4 dihedral angles in H2U roughly −50° and −10°, respectively, versus nearly perfect planarity in unmodified U. This deformation reduces the propensity of H2U to engage in perfectly planar base pairs, and even H2U:U tWW(#**19**) assumes a twisted propeller conformation. In terms of H-bonding propensity, reduction of conjugation to the N1-C2-N3-C4 atoms reinforces the H-bond accepting capability of both the O4 and the O2 atoms, by increasing their negative partial charge by roughly −0.01e. This may explain the slightly higher stability of the base pairs including H2U.
- The halide modification on uracil has stabilizing effect (with the exception of 5BrU:A cS(r)W(#**7’**) and 5BrU:A cS(r)H(#**6’**) pairs, which incorporate the ribose). The only base pair we observed with a halogenated cytosine was instead destabilized by the modification. Similar *E*_Mod_ calculated at the B3LYP and B3LYP-D3 levels indicates that, like methylation, dispersion interactions have a minor role in determining the modified base pair stability, thus suggesting that the impact of the halide can be explained considering its inductive effect. Halides are electron-withdrawing substituents, thus depleting electron density from the aromatic ring. This makes the O and N atoms in the ring poorer H-bond acceptors, while making the N-H groups better H-bond donors. In line with this scheme, the destabilizing effect of bromination in 5BrC:G cWW(#**1’**) is explained considering that 5BrC participates in the H-bonding through the N3 and O2 H-bond donors, which are poorer donors compared to the same atoms in unmodified C, while the exocyclic N4-H group would instead stabilize the base pair. The stabilizing effect of the halide when 5BrU and 5IU are involved can similarly be explained considering that they are engaged in the H-bonding through the endocyclic N3-H group, which is a better H-bond donor compared to the same atom in non-modified U. These non-natural modifications thus have a minor but not negligible effect on the energetics of base pairs hosting them, indicating that they could have an impact on the RNA folding analogous to that induced by naturally occurring modifications.

## SUPPLEMENTARY DATA

Supplementary Data are available at NAR Online.

SUPPLEMENTARY DATA
