# An atlas of RNA base pairs involving modified nucleobases with optimal geometries and accurate energies

**DOI:** 10.1093/nar/gkv925

**Published:** 2015-09-10

**Authors:** Mohit Chawla, Romina Oliva, Janusz M. Bujnicki, Luigi Cavallo

*Nucl. Acids Res*. 43 (14): 6714–6729. doi: 10.1093/nar/gkv606

The authors wish to make the following corrections to their article:

Throughout the article, in the main text, figures, tables, and in supplementary material, H2U is used as an abbreviation for dihydrouridine, while D should be used instead. H2U has been used earlier as an abbreviation for 4-pyrimidinone.

The results and conclusion of the article are not affected and remain valid. The authors apologise to the readers for the inconvenience caused.

