# Supplementary material for: An atlas of RNA base pairs involving modified nucleobases with optimal geometries and accurate energies
Source: Nucleic Acids Res. 2015 Jun 27;43(14):6714–29. doi: 10.1093/nar/gkv606 (PMC4538814; doi:10.1093/nar/gkv606)
Supplement: SUPPLEMENTARY DATA [file supp_43_14_6714__index.html]

An atlas of RNA base pairs involving modified nucleobases with optimal geometries and accurate energies — SUPPLEMENTARY DATA 

# An atlas of RNA base pairs involving modified nucleobases with optimal geometries and accurate energies

## SUPPLEMENTARY DATA

- SUPPLEMENTARY DATA
