# Supplementary material for: An atlas of RNA base pairs involving modified nucleobases with optimal geometries and accurate energies
Source: Nucleic Acids Res. 2015 Jun 27;43(14):6714–29. doi: 10.1093/nar/gkv606 (PMC4538814; doi:10.1093/nar/gkv606)
Supplement: SUPPLEMENTARY DATA [file supp_gkv606_nar-00960-h-2015-File011.docx]

**Supplementary Materials for:**

“An atlas of RNA base pairs involving modified nucleobases with optimal geometries and accurate energies”, by Chawla *et al*.

**Quantum mechanics calculations: optimal geometries and energies**

**1. Base pairs involving modified adenine**

***1a. 1-methyladenine (***m^1^A***)***

***Optimized geometries and energies****.* The optimized gas phase geometries for the four model systems involving m^1^A are similar to their corresponding experimental geometries, with conservation of the H-bonding patterns. Most differences in the H-bond distances between optimized and experimental pairs are within 0.23 Å, which is in the expected range for this kind of calculations ([28-31](#_ENREF_28),[51](#_ENREF_51)) However, in the m^1^A:A tHW pair, which has a planar geometry in the experimental context, while is slightly propeller twisted when optimized, the N6(A)-N7(m^1^A) H-bond is elongated by 0.35 Å. On the contrary, the N6(m^1^A)-O2(U) H-bond in m^1^A:U tHW and the N6(m^1^A)-O2(m^5^U) H-bond in m^1^A: m^5^U tWH are significantly shortened, by 0.43 Å and 0.53 Å, respectively. RMSD values for the heavy atoms superposition of optimized pairs on the corresponding experimentally determined ones are all within 0.26 Å, indicating a high similarity. As for the comparison of optimized modified pairs with corresponding optimized unmodified pairs, difference in the H-bond distances are within 0.30 Å (in few cases within 0.10 Å), whereas RMSD values are around 0.20-0.25 Å, showing that the modification does not have substantial impact on their geometry. The larger difference is observed for m^1^A:A tHW, as in the absence of the modification A:A tHW assumes an X-ray-like planar geometry (RMSD is 0.38 Å).

To investigate the possible stabilizing/destabilizing effect of the modifications, we also compared the interaction energy of the modified vs. the corresponding unmodified base pairs. In line with previous results ([28](#_ENREF_28),[31](#_ENREF_31)) the positive charge on m^1^A clearly stabilizes the modified base pairs (*E*_Mod_ in the range -6.0 to -11.0 kcal/mol). The highest stabilization, 11.0 kcal/mol, was observed for the water-mediated m^1^A:U tHW(w) base pair. Further, the interaction energy between the explicit water and the base pair in m^1^A:U tH:W(w) is -19.8 kcal/mol, thus indicating that the water molecule is firmly held in place.

***1b. N6-dimethyladenine (***m^6^_6_A***)***

***Optimized geometries and energies.*** The optimized m^6^_6_A:G tS(W)S(r) geometry deviates insignificantly from the experimentally determined geometry, with differences in the H-bond distances within 0.11 Å, except for N2(G)-N3(m^6^_6_A), which is elongated by 0.37 Å, and a RMSD from the experimental geometry of 0.45 Å. The optimized modified and corresponding unmodified pairs are very similar (H-bonds within 0.06 Å, RMSD of 0.16 Å). As for the interaction energy, the modified m^6^_6_A:G tS(W)S(r) pair, with a *E*_Mod_ of -1.2 kcal/mol, is more stable than the unmodified pair.

**2. Base pairs involving modified guanine**

***2a. 1-methylguanine (***m^1^G***)***

***Optimized geometry and energies.*** The optimized m^1^G:C tHH1 base pair geometry is very similar to the experimentally observed planar geometry, with only an elongation of the single N4(C)-N7(m^1^G) H-bond by 0.29 Å. Moreover, the geometry of m^1^G:C tHH1 is extremely similar to that of the unmodified pair (H-bonds distances within 0.02 Å and RMSD of 0.02 Å), with the modified pair marginally more stable, *E*_Mod_ of -0.3 kcal/mol, than the unmodified one.

***2b. N2-methylguanine (***m^2^G***)***

***Optimized geometries and energies.*** The optimized geometry of the m^2^G:U cWW pair is overall similar to the experimental geometry, with an elongation of N3(U)-O6(m^2^G) by 0.33 Å. The modified pair is in addition extremely similar to the corresponding unmodified one (H-bonds within 0.01 Å and RMSD of 0.05 Å). The modified pair, with a *E*_Mod_ of -0.2 kcal/mol, is slightly more stable than the unmodified pair. As explained above, m^2^G:C cWW exhibits two different conformations. Once optimized, the geometry of the m^2^G:C cWW base pair is extremely similar to the experimentally observed one (H-bonds within 0.05 Å and RMSD of 0.09 Å) as well as to the optimized geometry of the unmodified pair (H-bonds within 0.01 Å, RMSD of 0.01 Å). The *E*_Mod_ of -0.9 kcal/mol indicates that the modification increases the stability of the base pair. On the other hand, optimization of m^2^G:C cWW1, which is propeller-twisted in the experimentally observed structure, converges to a planar geometry, and after optimization the N1(m^2^G)-O2(C) H-bond breaks, and two Watson-Crick like H-bonds, N4-H(C)-O6(m^2^G) and N1-H(m^2^G)-N3(C), are formed. The optimized geometry thus significantly deviates from the experimentally determined structure with an RMSD of 0.75 Å. Not surprisingly, the optimized m^2^G:C WW1 geometry, with two H-bonds only and an *E*_Mod_ of 9.4 kcal/mol, is remarkably less stable than the corresponding ‘canonical’ unmodified pair, characterized by three H-bonds.

To rationalize if the stabilizing effect of the methyl group is due to inductive effects through the σ−bonds skeleton of G, or it is due to additional stabilizing dispersion interactions upon methylation, as exemplary cases we compared the *E*_Mod_ calculated with the B3LYP and the B3LYP-D3 methods for the m^2^G:U (#7) and the m^2^G:C cWW (#8) base pairs. The B3LYP method corresponds to the approach used in the geometry optimizations, the B3LYP-D3 method includes an explicit term to account for dispersion interactions ([53](#_ENREF_53)). The difference in the *E*_Mod_ calculated with the two methods, -0.1 and -0.2 kcal/mol for m^2^G:U cWW and m^2^G:C cWW, indicates that dispersion interactions contribute to a minor extent to the stability of the modified base pair.

***2c. N2,N2-dimethylguanine (***m^2^_2_G***)***

***Optimized geometries and energies.*** Both the optimized and experimentally observed geometries are substantially non-planar (differences in H-bond distances within 0.20 Å, RMSD of 0.51 Å). The m^2^_2_G:A cWW geometry is propeller-twisted to avoid the steric repulsion between the methyl groups on N2 of m^2^_2_G and the hydrogen on the C2 atom of the adenine. The unmodified geometry is more planar, although still slightly propeller-twisted, resulting in a RMSD value for superimposition of 0.55 Å, while H-bonds distances are similar in both structures. The unmodified G:A cWW pair, with an *E*_Mod_ of -1.1 cal/mol, is more stable than the modified pair.

***2d. 7-methylguanine (***m^7^G***)***

***Optimized geometries and energies.*** The geometry of the optimized m^7^G:C cWW pair is quite similar to the experimentally determined geometry (H-bond distances within 0.26 Å and RMSD of 0.21 Å). Similarly, the modified and unmodified pairs are also very similar (H-bond distances within 0.15 Å, RMSD of 0.08 Å). Similar good agreement between the optimized and the experimental geometry is found for the m^7^G:G tWH base pair (H-bonds within 0.17 Å and RMSD of 0.22 Å). The m^7^G modification introduces a positive charge and stabilizes the m^7^G:C cWW and m^7^G:G tWH base pairs, with an *E*_Mod_ of -10.2 and -15.1 kcal/mol.

**3. Base pairs involving cytosine modification**

***3a. 5-methylcytosine (***m^5^C***)***

***Optimized geometries and energies.*** The geometry of the optimized m^5^C:G cWW pair is similar to the experimentaly observed geometry, with the exception of the shortening of the N4(m^5^C)-O6(G) H-bond by 0.56 Å. The optimized modified and the corresponding unmodified pairs are very similar to each other (H-bonds within 0.01 Å, and RMSD of 0.01 Å). The m^5^C:G cWW base pair, with an *E*_Mod_ of -0.7 kcal/mol, is more stable than the unmodified pair. As for m^5^C:G tWW, it is not geometrically stable as an isolated base pair in the gas phase. Similarly to what we have previously shown for the G:C tWW pair, due to repulsive amino-amino and carbonyl-carbonyl contacts, during the gas phase optimization, the double H-bond stabilized m^5^C:G tWW geometry moves to a bifurcated H-bond pattern, with breaking of the N2(G)-N3(m^5^C) H-bond seen in the experimentally determined structure ([28](#_ENREF_28),[30](#_ENREF_30),[31](#_ENREF_31)). This severe geometric rearrangement leads to an RMSD of 1.28 Å of the optimized vs. the experimentally determined structure. The optimized m^5^C:G tWW base pair, with an *E*_Mod_ of -0.5 kcal/mol, is slightly more stable than the unmodified C:G tWW pair. Finally, comparison of the *E*_Mod_ calculated with the B3LYP and the B3LYP-D3 methods for the m^5^C:G cWW base pair, used as test case, indicates again that dispersion interactions contribute to a minor extent to the stability of the modified base pair, since the B3LYP-D3 *E*_Mod_ is only -0.1 kcal/mol lower than *E*_Mod_ calculated at the B3LYP level.

**4. Base pairs involving uracil modification**

***4a. 5-methyluracil (m^5^U)***

***Optimized geometries and energies.*** The optimized m^5^U:A tWH pair geometry is very similar to the experimentally determined one, with minimal alterations of the H-bonding distances (within 0.19 Å) and a RMSD of 0.20 Å. Its geometry is also coincident with that of the corresponding unmodified pair (H-bonds within 0.01 Å, RMSD = 0.0 Å), with minimal impact on stability, with an *E*_Mod_ of only -0.1 kcal/mol. The second optimized pair, m^5^U:G cWW, is also very similar to the experimentally determined structure, with just an elongation of the N3(m^5^U)-O6(G) H-bond by 0.29 Å, but modified pair is slightly more stable, with an *E*_Mod_ of -0.4 kcal/mol, than its unmodified counterpart. Finally, the geometry of the m^5^U:G tWH1 base pair, which is planar in the experimental structure and stabilized by a single strong H-bond between N3(m^5^U)-N7(G), after optimization undergoes an opening with an elongation of the N3(m^5^U)-N7(G) H-bond by 0.49 Å and a RMSD of 0.54 Å, while staying planar. The elongation in the N3(m^5^U)-N7(G) distance is probably consequence of the repulsion between the negatively charged O2(m^5^U) and O6(G) atoms, only 2.55 Å apart in the experimental structure. The optimized geometry of m^5^U:G tWH1 is instead extremely similar to that of the unmodified pair and, with an *E*_Mod_ of -0.2 kcal/mol, slightly more stable. Finally, also in this case we compared the *E*_Mod_ calculated with the B3LYP and the B3LYP-D3 methods for an exemplary case, specifically for the m^5^U:G cWW base pair. Also in this case we found that dispersion interactions contribute to a minor extent to the stability of the modified base pair, since the B3LYP-D3 *E*_Mod_ is only -0.1 kcal/mol lower than *E*_Mod_ calculated at the B3LYP level.

***4b. 4-thiouracil (s^4^U)***

***Optimized geometries and energies.*** The optimized s^4^U:A tWH pair has a geometry similar to the experimentally determined one (differences in H-bond distances within 0.31 Å and RMSD = 0.18 Å). Furthermore, the geometry of the optimized modified pair is very similar to that of the unmodified one. The s^4^U:A tWH interaction is marginally stronger, with an *E*_Mod_ of -0.4 kcal/mol, than the unmodified counterpart.

***4c. Dihydrouracil (H2U)***

***Optimized geometries and energies.*** The experimentally determined H2U:U tWW pair is slightly propeller-twisted. However, upon optimization, the base pair gets planar, with an elongation of the N3(H2U)-O2(U) and N3(U)-O2(H2U) H-bonds by 0.24 Å and 0.21 Å, respectively and an RMSD from the X-ray structure of 0.30 Å. This elongation could be due to repulsion between the exocyclic carbonyl groups of the two nucleobases in the experimentally determined structure [O2(U)-O4(H2U) and O4(H2U)-O2(U) distances are 3.2/3.7 Å]. A similar elongation in the H-bond distances is also observed for the optimized unmodified pair U:U tWW, which is more planar. H2U:U tWW is marginally more stable than the unmodified U:U tWW pair, with *E*_Mod_ of -0.02 kcal/mol,. The H2U:G cHS1 pair, stabilized by one strong H-bond and assuming experimentally a planar geometry, after optimization loses planarity with the two bases becoming almost perpendicular, while the N2(G)-O4(H2U) H-bond is elongated by 0.47 Å. Such rearrangement also implies a shortening of the distance between the two C1′ atoms, from 6.3 to 5.3 Å. This value is not realistic for two consecutive nucleobases, (this is indeed a dinucleotide platform), therefore we decided to simulate the entire two nucleotides, with their ribose-phosphate backbone. As a result, we found a C1′-C1′ distance of 7.16 Å, although a similar elongation in the N2(G)-O4(H2U) H-bond, by 0.54 Å, and an optimized geometry substantially propeller twisted was observed. Further, upon including the ribose-phosphate backbone, the RMSD value of the optimized base pair geometry *vs.* the X-ray one and *vs.* the optimized unmodified geometry is moderately reduced, to 0.89 and 0.86 Å respectively (it was 1.08 Å and 1.27 Å without the ribose-phosphate backbone). Conversely, optimization of the corresponding unmodified base pair, U:G cHS1, resulted in a planar geometry quite similar to the experimentally determined one. Nevertheless, an elongation of 0.40 Å was still observed for the N2(G)-O4(U) H-bond. Finally, the modified H2U:G cHS1 pair is slightly more stable, with an *E*_Mod_ of 0.3 kcal/mol, than its unmodified counterpart. For the H2U:G tWS pair, the experimental and optimized geometries exhibit a similar propeller-twist (RMSD is 0.33 Å). Optimized modified and unmodified pairs are highly similar. The modified pair is slightly more stable, *E*_Mod_ of only -0.1 kcal/mol.

***4d. Pseudouracil (***Ψ***)***

***Optimized geometries and energies.*** Overall the optimized geometries of base pairs involving Ψ are quite similar to the experimentally observed geometries. In particular, RMSD values for the superimposition on the X-ray structures are within 0.18 Å and differences in H-bond distances within 0.10 Å for Ψ:A cWW, Ψ:U tWW, and Ψ:G cWW, while all the RMSD values are within 0.32 Å and all differences in H–bonds within 0.24 Å. The only exception is represented by the N6(A)-O2(Ψ) H-bond in Ψ:A cHW, which is shortened by 0.36 Å upon optimization. As for comparison with corresponding unmodified paàirs, again the Ψ:A cWW, Ψ:U tWW, and Ψ:G cWW base pairs have optimized geometries extremely similar to those of the corresponding unmodified pairs (H-bonds within 0.03 Å and RMSD within 0.06 Å). The Ψ:G tBsW (slightly twisted) and Ψ:C cSW pairs are also quite similar to the corresponding unmodified pairs (H-bonds within 0.08 Å and RMSD within 0.13 Å).

The Ψ:A cHW pair represents instead a special case, as substituting the Ψ with an unmodified U in the same orientation means losing one H-bonding donor, N1, which is substituted by C5. The H-bonding acceptor O2 is instead substituted by an “equivalent” O4. Therefore, energy optimization of U:A cHW results in an opening of the base pair from the minor groove with a remarkable elongation of the C5(U)-N1(A) distance (it was N1-N1 in Ψ), by 0.75 Å. The RMSD for the superimposition of the modified and unmodified pairs is 0.36 Å.

The Ψ:A cHW pair, with one more H-bond than its unmodified counterpart, is clearly more stable, with an *E*_Mod_ of -6.9 kcal/mol. The only other pair containing a Ψ where modification stabilizes the base pair, with an *E*_Mod_ of -1.2 kcal/mol, is the ‘wobble’ Ψ:G cWW. All the remaining four base pairs containing Ψ, Ψ:A cWW, Ψ:U tWW, Ψ:G tBsW, and Ψ:C cSW are less stable than the corresponding unmodified pairs, with an *E*_Mod_ of 0.4 to 1.6 kcal/mol.

The Ψ:A cHW pair represents instead a special case, as substituting the Ψ with an unmodified U in the same orientation means losing one H-bonding donor, N1, which is substituted by C5. The H-bonding acceptor O2 is instead substituted by an “equivalent” O4. Therefore, energy optimization of U:A cHW results in an opening of the base pair from the minor groove with a remarkable elongation of the C5(U)-N1(A) distance (it was N1-N1 in Ψ), by 0.75 Å. The Ψ:A cHW pair, with one more H-bond than its unmodified counterpart, is clearly more stable, with an *E*_Mod_ of -6.9 kcal/mol.

**5. Base pairs involving non-natural modifications**

***5a. 5-Bromocytosine (5BrC) and 5-Bromouracil (5BrU)***

***Optimized geometries and energies.*** The 5BrC:G cWW base pair geometry is very similar to the experimentally determined one, with differences in H-bond distances within 0.18 Å and an RMSD of 0.21 Å. Similarly, the modified geometry corresponding to 5BrC:G cWW is very similar to that of the unmodified C:G cWW, with differences in H-bonds within 0.01 Å and an RMSD of 0.01 Å. Nevertheless, the modified base pair, with an *E*_Mod_ of 0.4 kcal/mol, is slightly less stable than the unmodified pair. The optimized geometry for the six base pair models involving one 5BrU are extremely similar to the experimental geometries, with no alteration of the H-bonding pattern. The difference in most of the H-bond distances is normally within 0.25 Å, with the single exception of N3(5BrU)-O2(5BrU), in 5BrU-5BrU cWW, which is remarkably elongated upon optimization, by 0.50 Å. For the two systems 5BrU:A cSH and 5BrU:A cWS, ribose was also included in the calculations, due to involvement of O2’ atom in H-bonding interactions. However, upon optimization, a certain deformation, as compared to the experimental geometry, is observed for 5BrU:A cS(r)H and 5BrU:A cW(r)S, which is reflected by large RMSD values of 1.05 Å and 0.80 Å, respectively (RMSD on the nucleobases are still as high as 0.90 Å and 0.73 Å). Indeed, while the 5BrU:A cW(r)S experimental structure is planar, the optimized one appears substantially propeller twisted. The 5BrU:A cS(r)H X-ray geometry is instead slightly twisted and becomes buckled upon optimization. Nevertheless, the optimized H-bonds for these two geometries are within 0.21 Å from the experimental values. As for 5BrU, base pairs presenting such modification are in very good agreement with the geometries of corresponding unmodified pairs (RMSD within 0.07 Å). Moving to energies, most of the modified pairs, namely 5BrU:A cWW, 5BrU:G cWW, 5BrU:5BrU cWW and 5BrU:A cWH, with an *E*_Mod_ of -0.4 kcal/mol, are marginally more stable than their corresponding unmodified pair, while the stability of the 5BrU:A cS(r)H and 5BrU:A cW(r)S base pairs, with an *E*_Mod_ less than 0.04 kcal/mol, is basically identical to that of the corresponding unmodified pairs.

To test if the more polarizable halides could result in a greater contribution of dispersion interactions to *E*_Mod_, relative to the case of methyl modified base pairs, we compared the *E*_Mod_ calculated with the B3LYP and the B3LYP-D3 methods for an exemplary case, specifically for the 5BrU:G cWW base pair. Nevertheless, also in this case we found that dispersion interactions contribute to a minor extent to the stability of the modified base pair, since the B3LYP-D3 *E*_Mod_ is less than -0.1 kcal/mol lower than *E*_Mod_ calculated at the B3LYP level.

***5b. 5-Iodouracil (5IU)***

***Optimized geometries and energies.*** The optimized geometry of the 5IU:A cWW and 5IU:G cWW base pairs is extremely similar to the corresponding experimental geometry (differences in H-bond distances within 0.11 Å, and RMSD within 0.22 Å). Similarly, optimized modified and corresponding unmodified geometries for 5IU:A cWW and 5IU:G cWW are in very good agreement (H-bonds within 0.03 Å and RMSD within 0.07 Å). Finally, the modified pairs 5IU:A cWW and 5IU:G cWW, with an *E*_Mod_ of -0.4 kcal/mol, are slightly more stable than the unmodified U:A cWW and U:G cWW pairs.

**References**

1. Mercer, T.R., Gerhardt, D.J., Dinger, M.E., Crawford, J., Trapnell, C., Jeddeloh, J.A., Mattick, J.S. and Rinn, J.L. (2012) Targeted RNA sequencing reveals the deep complexity of the human transcriptome. *Nature biotechnology*, **30**, 99-104.

2. Cantara, W.A., Crain, P.F., Rozenski, J., McCloskey, J.A., Harris, K.A., Zhang, X., Vendeix, F.A., Fabris, D. and Agris, P.F. (2011) The RNA Modification Database, RNAMDB: 2011 update. *Nucleic acids research*, **39**, D195-201.

3. Machnicka, M.A., Milanowska, K., Osman Oglou, O., Purta, E., Kurkowska, M., Olchowik, A., Januszewski, W., Kalinowski, S., Dunin-Horkawicz, S., Rother, K.M. *et al.* (2013) MODOMICS: a database of RNA modification pathways--2013 update. *Nucleic acids research*, **41**, D262-267.

4. Maden, E.H. and Wakeman, J.A. (1988) Pseudouridine distribution in mammalian 18 S ribosomal RNA. A major cluster in the central region of the molecule. *The Biochemical journal*, **249**, 459-464.

5. Maden, B.E. (2001) Mapping 2'-O-methyl groups in ribosomal RNA. *Methods*, **25**, 374-382.

6. Lane, B.G., Ofengand, J. and Gray, M.W. (1992) Pseudouridine in the large-subunit (23 S-like) ribosomal RNA. The site of peptidyl transfer in the ribosome? *FEBS letters*, **302**, 1-4.

7. Del Campo, M., Recinos, C., Yanez, G., Pomerantz, S.C., Guymon, R., Crain, P.F., McCloskey, J.A. and Ofengand, J. (2005) Number, position, and significance of the pseudouridines in the large subunit ribosomal RNA of Haloarcula marismortui and Deinococcus radiodurans. *Rna*, **11**, 210-219.

8. Agris, P.F. (1996) The importance of being modified: roles of modified nucleosides and Mg2+ in RNA structure and function. *Progress in nucleic acid research and molecular biology*, **53**, 79-129.

9. Dunkle, J.A., Vinal, K., Desai, P.M., Zelinskaya, N., Savic, M., West, D.M., Conn, G.L. and Dunham, C.M. (2014) Molecular recognition and modification of the 30S ribosome by the aminoglycoside-resistance methyltransferase NpmA. *Proceedings of the National Academy of Sciences of the United States of America*, **111**, 6275-6280.

10. Hoerter, J.A. and Walter, N.G. (2007) Chemical modification resolves the asymmetry of siRNA strand degradation in human blood serum. *Rna*, **13**, 1887-1893.

11. Engelke, D.R. and Hopper, A.K. (2006) Modified view of tRNA: stability amid sequence diversity. *Molecular cell*, **21**, 144-145.

12. Motorin, Y. and Helm, M. (2010) tRNA stabilization by modified nucleotides. *Biochemistry*, **49**, 4934-4944.

13. Yi, C. and Pan, T. (2011) Cellular dynamics of RNA modification. *Accounts of chemical research*, **44**, 1380-1388.

14. Carell, T., Brandmayr, C., Hienzsch, A., Muller, M., Pearson, D., Reiter, V., Thoma, I., Thumbs, P. and Wagner, M. (2012) Structure and function of noncanonical nucleobases. *Angewandte Chemie*, **51**, 7110-7131.

15. Helm, M. (2006) Post-transcriptional nucleotide modification and alternative folding of RNA. *Nucleic acids research*, **34**, 721-733.

16. Karijolich, J., Kantartzis, A. and Yu, Y.T. (2010) RNA modifications: a mechanism that modulates gene expression. *Methods in molecular biology*, **629**, 1-19.

17. Hori, H. (2014) Methylated nucleosides in tRNA and tRNA methyltransferases. *Frontiers in genetics*, **5**, 144.

18. Torres, A.G., Batlle, E. and Ribas de Pouplana, L. (2014) Role of tRNA modifications in human diseases. *Trends in molecular medicine*, **20**, 306-314.

19. Berman, H., Henrick, K., Nakamura, H. and Markley, J.L. (2007) The worldwide Protein Data Bank (wwPDB): ensuring a single, uniform archive of PDB data. *Nucleic acids research*, **35**, D301-303.

20. Ennifar, E., Bernacchi, S., Wolff, P. and Dumas, P. (2007) Influence of C-5 halogenation of uridines on hairpin versus duplex RNA folding. *Rna*, **13**, 1445-1452.

21. Sponer, J.E., Spackova, N., Leszczynski, J. and Sponer, J. (2005) Principles of RNA base pairing: structures and energies of the trans Watson-Crick/sugar edge base pairs. *The journal of physical chemistry. B*, **109**, 11399-11410.

22. Sponer, J.E., Spackova, N., Kulhanek, P., Leszczynski, J. and Sponer, J. (2005) Non-Watson-Crick base pairing in RNA. quantum chemical analysis of the cis Watson-Crick/sugar edge base pair family. *The journal of physical chemistry. A*, **109**, 2292-2301.

23. Sponer, J.E., Leszczynski, J., Sychrovsky, V. and Sponer, J. (2005) Sugar edge/sugar edge base pairs in RNA: stabilities and structures from quantum chemical calculations. *The journal of physical chemistry. B*, **109**, 18680-18689.

24. Sponer, J., Mladek, A., Sponer, J.E., Svozil, D., Zgarbova, M., Banas, P., Jurecka, P. and Otyepka, M. (2012) The DNA and RNA sugar-phosphate backbone emerges as the key player. An overview of quantum-chemical, structural biology and simulation studies. *Physical chemistry chemical physics : PCCP*, **14**, 15257-15277.

25. Sponer, J., Jurecka, P. and Hobza, P. (2004) Accurate interaction energies of hydrogen-bonded nucleic acid base pairs. *Journal of the American Chemical Society*, **126**, 10142-10151.

26. Sharma, P., Sharma, S., Chawla, M. and Mitra, A. (2009) Modeling the noncovalent interactions at the metabolite binding site in purine riboswitches. *Journal of molecular modeling*, **15**, 633-649.

27. Sharma, P., Chawla, M., Sharma, S. and Mitra, A. (2010) On the role of Hoogsteen:Hoogsteen interactions in RNA: ab initio investigations of structures and energies. *Rna*, **16**, 942-957.

28. Oliva, R., Cavallo, L. and Tramontano, A. (2006) Accurate energies of hydrogen bonded nucleic acid base pairs and triplets in tRNA tertiary interactions. *Nucleic acids research*, **34**, 865-879.

29. Chawla, M., Sharma, P., Halder, S., Bhattacharyya, D. and Mitra, A. (2011) Protonation of base pairs in RNA: context analysis and quantum chemical investigations of their geometries and stabilities. *The journal of physical chemistry. B*, **115**, 1469-1484.

30. Chawla, M., Abdel-Azeim, S., Oliva, R. and Cavallo, L. (2014) Higher order structural effects stabilizing the reverse Watson-Crick Guanine-Cytosine base pair in functional RNAs. *Nucleic acids research*, **42**, 714-726.

31. Oliva, R., Tramontano, A. and Cavallo, L. (2007) Mg2+ binding and archaeosine modification stabilize the G15 C48 Levitt base pair in tRNAs. *Rna*, **13**, 1427-1436.

32. Zirbel, C.L., Sponer, J.E., Sponer, J., Stombaugh, J. and Leontis, N.B. (2009) Classification and energetics of the base-phosphate interactions in RNA. *Nucleic acids research*, **37**, 4898-4918.

33. Sharma, P., Sponer, J.E., Sponer, J., Sharma, S., Bhattacharyya, D. and Mitra, A. (2010) On the role of the cis Hoogsteen:sugar-edge family of base pairs in platforms and triplets-quantum chemical insights into RNA structural biology. *The journal of physical chemistry. B*, **114**, 3307-3320.

34. Bullock, T.L., Sherlin, L.D. and Perona, J.J. (2000) Tertiary core rearrangements in a tight binding transfer RNA aptamer. *Nature structural biology*, **7**, 497-504.

35. Leontis, N.B., Stombaugh, J. and Westhof, E. (2002) The non-Watson-Crick base pairs and their associated isostericity matrices. *Nucleic acids research*, **30**, 3497-3531.

36. Leontis, N.B. and Westhof, E. (2001) Geometric nomenclature and classification of RNA base pairs. *Rna*, **7**, 499-512.

37. Lemieux, S. and Major, F. (2002) RNA canonical and non-canonical base pairing types: a recognition method and complete repertoire. *Nucleic acids research*, **30**, 4250-4263.

38. Yang, H., Jossinet, F., Leontis, N., Chen, L., Westbrook, J., Berman, H. and Westhof, E. (2003) Tools for the automatic identification and classification of RNA base pairs. *Nucleic acids research*, **31**, 3450-3460.

39. Sponer, J.E., Reblova, K., Mokdad, A., Sychrovsky, V., Leszczynski, J. and Sponer, J. (2007) Leading RNA tertiary interactions: structures, energies, and water insertion of A-minor and P-interactions. A quantum chemical view. *The journal of physical chemistry. B*, **111**, 9153-9164.

40. Becke, A.D. (1996) Density-functional thermochemistry. *Abstr Pap Am Chem S*, **212**, 112-COMP.

41. Becke, A.D. (1993) Density-Functional Thermochemistry .3. The Role of Exact Exchange. *J Chem Phys*, **98**, 5648-5652.

42. Dunning, T.H. (1989) Gaussian-Basis Sets for Use in Correlated Molecular Calculations .1. The Atoms Boron through Neon and Hydrogen. *J Chem Phys*, **90**, 1007-1023.

43. Moller, C. and Plesset, M.S. (1934) Note on an approximation treatment for many-electron systems. *Phys Rev*, **46**, 0618-0622.

44. Weigend, F. and Haser, M. (1997) RI-MP2: first derivatives and global consistency. *Theor Chem Acc*, **97**, 331-340.

45. Boys, S.F. and Bernardi, F. (1970) Calculation of Small Molecular Interactions by Differences of Separate Total Energies - Some Procedures with Reduced Errors. *Mol Phys*, **19**, 553-&.

46. Charette, M. and Gray, M.W. (2000) Pseudouridine in RNA: what, where, how, and why. *IUBMB life*, **49**, 341-351.

47. Carlile, T.M., Rojas-Duran, M.F., Zinshteyn, B., Shin, H., Bartoli, K.M. and Gilbert, W.V. (2014) Pseudouridine profiling reveals regulated mRNA pseudouridylation in yeast and human cells. *Nature*.

48. Anderson, J., Phan, L., Cuesta, R., Carlson, B.A., Pak, M., Asano, K., Bjork, G.R., Tamame, M. and Hinnebusch, A.G. (1998) The essential Gcd10p-Gcd14p nuclear complex is required for 1-methyladenosine modification and maturation of initiator methionyl-tRNA. *Genes & development*, **12**, 3650-3662.

49. Anderson, J., Phan, L. and Hinnebusch, A.G. (2000) The Gcd10p/Gcd14p complex is the essential two-subunit tRNA(1-methyladenosine) methyltransferase of Saccharomyces cerevisiae. *Proceedings of the National Academy of Sciences of the United States of America*, **97**, 5173-5178.

50. Li, Z., Gillis, K.A., Hegg, L.A., Zhang, J. and Thurlow, D.L. (1996) Effects of nucleotide substitutions within the T-loop of precursor tRNAs on interaction with ATP/CTP:tRNA nucleotidyltransferases from Escherichia coli and yeast. *The Biochemical journal*, **314 ( Pt 1)**, 49-53.

51. Oliva, R. and Cavallo, L. (2009) Frequency and effect of the binding of Mg2+, Mn2+, and Co2+ ions on the guanine base in Watson-Crick and reverse Watson-Crick base pairs. *The journal of physical chemistry. B*, **113**, 15670-15678.

52. Schmeing, T.M., Huang, K.S., Strobel, S.A. and Steitz, T.A. (2005) An induced-fit mechanism to promote peptide bond formation and exclude hydrolysis of peptidyl-tRNA. *Nature*, **438**, 520-524.

53. Grimme, S., Antony, J., Ehrlich, S. and Krieg, H. (2010) A consistent and accurate ab initio parametrization of density functional dispersion correction (DFT-D) for the 94 elements H-Pu. *J Chem Phys*, **132**.

54. Adams, P.L., Stahley, M.R., Kosek, A.B., Wang, J.M. and Strobel, S.A. (2004) Crystal structure of a self-splicing group I intron with both exons. *Nature*, **430**, 45-50.

55. Gate, J.H., Gooding, A.R., Podell, E., Zhou, K.H., Golden, B.L., Szewczak, A.A., Kundrot, C.E., Cech, T.R. and Doudna, J.A. (1996) RNA tertiary structure mediation by adenosine platforms. *Science*, **273**, 1696-1699.

56. Lu, X.J., Olson, W.K. and Bussemaker, H.J. (2010) The RNA backbone plays a crucial role in mediating the intrinsic stability of the GpU dinucleotide platform and the GpUpA/GpA miniduplex. *Nucleic acids research*, **38**, 4868-4876.

57. Moulinier, L., Eiler, S., Eriani, G., Gangloff, J., Thierry, J.C., Gabriel, K., McClain, W.H. and Moras, D. (2001) The structure of an AspRS-tRNA(Asp) complex reveals a tRNA-dependent control mechanism. *Embo J*, **20**, 5290-5301.

58. Nissen, P., Kjeldgaard, M., Thirup, S., Polekhina, G., Reshetnikova, L., Clark, B.F. and Nyborg, J. (1995) Crystal structure of the ternary complex of Phe-tRNAPhe, EF-Tu, and a GTP analog. *Science*, **270**, 1464-1472.

59. Lin, Y. and Kielkopf, C.L. (2008) X-ray structures of U2 snRNA-branchpoint duplexes containing conserved pseudouridines. *Biochemistry*, **47**, 5503-5514.

60. Zhanpeisov, N.U., Sponer, J. and Leszczynski, J. (1998) Reverse Watson-Crick isocytosine-cytosine and guanine-cytosine base pairs stabilized by the formation of the minor tautomers of bases. An ab initio study in the gas phase and in a water cluster. *Journal of Physical Chemistry A*, **102**, 10374-10379.

61. Watanabe, K., Shinma, M., Oshima, T. and Nishimura, S. (1976) Heat-Induced Stability of Transfer-Rna from an Extreme Thermophile, Thermus-Thermophilus. *Biochem Bioph Res Co*, **72**, 1137-1144.

62. Kowalak, J.A., Dalluge, J.J., Mccloskey, J.A. and Stetter, K.O. (1994) The Role of Posttranscriptional Modification in Stabilization of Transfer-Rna from Hyperthermophiles. *Biochemistry*, **33**, 7869-7876.

63. Horie, N., Harayokoyama, M., Yokoyama, S., Watanabe, K., Kuchino, Y., Nishimura, S. and Miyazawa, T. (1985) 2 Transfer-Rna 1ile Species from an Extreme Thermophile, Thermus-Thermophilus Hb8 - Effect of 2-Thiolation of Ribothymidine on the Thermostability of Transfer-Rna. *Biochemistry*, **24**, 5711-5715.

64. Steinberg, S. and Cedergren, R. (1995) A correlation between N-2-dimethylguanosine presence and alternate tRNA conformers. *Rna-a Publication of the Rna Society*, **1**, 886-891.

65. Urbonavicius, J., Armengaud, J. and Grosjean, H. (2006) Identity elements required for enzymatic formation of N-2, N-2-dimethylguanosine from N-2-monomethylated derivative and its possible role in avoiding alternative conformations in archaeal tRNA. *Journal of molecular biology*, **357**, 387-399.

66. Helm, M., Brule, H., Degoul, F., Cepanec, C., Leroux, J.P., Giege, R. and Florentz, C. (1998) The presence of modified nucleotides is required for cloverleaf folding of a human mitochondrial tRNA. *Nucleic acids research*, **26**, 1636-1643.

67. Helm, M., Giege, R. and Florentz, C. (1999) A Watson-Crick base-pair-disrupting methyl group (m1A9) is sufficient for cloverleaf folding of human mitochondrial tRNALys. *Biochemistry*, **38**, 13338-13346.

68. Voigts-Hoffmann, F., Hengesbach, M., Kobitski, A.Y., van Aerschot, A., Herdewijn, P., Nienhaus, G.U. and Helm, M. (2007) A methyl group controls conformational equilibrium in human mitochondrial tRNA(Lys). *Journal of the American Chemical Society*, **129**, 13382-+.

69. Sumita, M., Desaulniers, J.P., Chang, Y.C., Chui, H.M., Clos, L., 2nd and Chow, C.S. (2005) Effects of nucleotide substitution and modification on the stability and structure of helix 69 from 28S rRNA. *Rna*, **11**, 1420-1429.

70. Meroueh, M., Grohar, P.J., Qiu, J., SantaLucia, J., Jr., Scaringe, S.A. and Chow, C.S. (2000) Unique structural and stabilizing roles for the individual pseudouridine residues in the 1920 region of Escherichia coli 23S rRNA. *Nucleic acids research*, **28**, 2075-2083.

71. Ofengand, J., Bakin, A., Wrzesinski, J., Nurse, K. and Lane, B.G. (1995) The pseudouridine residues of ribosomal RNA. *Biochemistry and cell biology = Biochimie et biologie cellulaire*, **73**, 915-924.

72. Davis, D.R. (1995) Stabilization of RNA stacking by pseudouridine. *Nucleic acids research*, **23**, 5020-5026.

73. Jiang, J., Aduri, R., Chow, C.S. and SantaLucia, J., Jr. (2014) Structure modulation of helix 69 from Escherichia coli 23S ribosomal RNA by pseudouridylations. *Nucleic acids research*, **42**, 3971-3981.

74. Arnez, J.G. and Steitz, T.A. (1994) Crystal structure of unmodified tRNA(Gln) complexed with glutaminyl-tRNA synthetase and ATP suggests a possible role for pseudo-uridines in stabilization of RNA structure. *Biochemistry*, **33**, 7560-7567.

75. Newby, M.I. and Greenbaum, N.L. (2002) Investigation of Overhauser effects between pseudouridine and water protons in RNA helices. *Proceedings of the National Academy of Sciences of the United States of America*, **99**, 12697-12702.

76. Dalluge, J.J., Hashizume, T., Sopchik, A.E., McCloskey, J.A. and Davis, D.R. (1996) Conformational flexibility in RNA: The role of dihydrouridine. *Nucleic acids research*, **24**, 1073-1079.

**Table S1.** Complete list of the occurrences in PDB structures of different modified base pairs

| **Base pair** | **Occurence** | **PDB ID** | **Chain ID** | **Res1 ID** | **Res2 ID** | **Additional moiety** |
| --- | --- | --- | --- | --- | --- | --- |
| **m^1^A:A tHW** | 1 | 1YFG | A | 58 | 54 |  |
| **m^1^A:U tH** | 1 | 1OB2 | B | 58 | 54 |  |
| **m^1^A:U tHW(w)** | 1 | 1S72 | 0 | 628 | 624 | W9644 |
|  | 2 | 1VQ4 | 0 | 628 | 624 | W3434 |
|  | 3 | 1VQ5 | 0 | 628 | 624 | W3446 |
|  | 4 | 1VQ6 | 0 | 628 | 624 | W3432 |
|  | 5 | 1VQ7 | 0 | 628 | 624 | W3447 |
|  | 6 | 1VQ8 | 0 | 628 | 624 | W3728 |
|  | 7 | 1VQ9 | 0 | 628 | 624 | W3705 |
|  | 8 | 1VQK | 0 | 628 | 624 | W3736 |
|  | 9 | 1VQL | 0 | 628 | 624 | W3725 |
|  | 10 | 1VQM | 0 | 628 | 624 | W3731 |
|  | 11 | 1VQN | 0 | 628 | 624 | W3727 |
|  | 12 | 1VQO | 0 | 628 | 624 | W3746 |
|  | 13 | 1VQP | 0 | 628 | 624 | W3738 |
|  | 14 | 1YHQ | 0 | 628 | 624 | W3625 |
|  | 15 | 1YI2 | 0 | 628 | 624 | W3126 |
|  | 16 | 1YIJ | 0 | 628 | 624 | W3134 |
|  | 17 | 1YIT | 0 | 628 | 624 | w2632 |
|  | 18 | 1YJ9 | 0 | 628 | 624 | W9938 |
|  | 19 | 1YJN | 0 | 628 | 624 | W3132 |
|  | 20 | 1YJW | 0 | 628 | 624 | W5416 |
|  | 21 | 2OTJ | 0 | 628 | 624 | W3138 |
|  | 22 | 2OTL | 0 | 628 | 624 | W3130 |
|  | 23 | 2QEX | 0 | 628 | 624 | W9954 |
|  | 24 | 3CC2 | 0 | 628 | 624 | W9669 |
|  | 25 | 3CC4 | 0 | 628 | 624 | W3163 |
|  | 26 | 3CC7 | 0 | 628 | 624 | W3160 |
|  | 27 | 3CCE | 0 | 628 | 624 | W3145 |
|  | 28 | 3CCJ | 0 | 628 | 624 | W3126 |
|  | 29 | 3CCL | 0 | 628 | 624 | W3146 |
|  | 30 | 3CCM | 0 | 628 | 624 | W3148 |
|  | 31 | 3CCQ | 0 | 628 | 624 | W3145 |
|  | 32 | 3CCR | 0 | 628 | 624 | W3132 |
|  | 33 | 3CCS | 0 | 628 | 624 | W3128 |
|  | 34 | 3CCU | 0 | 628 | 624 | W3140 |
|  | 35 | 3CCV | 0 | 628 | 624 | W3135 |
|  | 36 | 3CD6 | 0 | 628 | 624 | W3150 |
|  | 37 | 3CMA | 0 | 628 | 624 | W4026 |
|  | 38 | 3CME | 0 | 628 | 624 | W4032 |
|  | 39 | 3G4S | 0 | 628 | 624 | W7070 |
|  | 40 | 3G6E | 0 | 628 | 624 | W7177 |
|  | 41 | 3G71 | 0 | 628 | 624 | W7249 |
|  | 42 | 3I55 | 0 | 628 | 624 | W6756 |
|  | 43 | 3I56 | 0 | 628 | 624 | W7276 |
|  | 44 | 4HUB | 0 | 628 | 624 | - |
| **m^1^A: m^5^U** | 1 | 1EHZ | A | 58 | 54 |  |
|  | 2 | 1EVV | A | 58 | 54 |  |
|  | 3 | 1F7U | B | 58 | 54 |  |
|  | 4 | 1F7V | B | 58 | 54 |  |
|  | 5 | 1FIR | A | 58 | 54 |  |
|  | 6 | 1TN1 | A | 58 | 54 |  |
|  | 7 | 1TN2 | A | 58 | 54 |  |
|  | 8 | 1TRA | A | 58 | 54 |  |
|  | 9 | 1TTT | D | 58 | 54 |  |
|  | 10 | 1TTT | E | 58 | 54 |  |
|  | 11 | 1TTT | F | 58 | 54 |  |
|  | 12 | 2DLC | Y | 58 | 54 |  |
|  | 13 | 4TNA | A | 58 | 54 |  |
|  | 14 | 4TRA | A | 58 | 54 |  |
|  | 15 | 6TNA | A | 58 | 54 |  |
|  | 16 | 1H3E | B | 58 | 54 |  |
|  | 17 | 1OB5 | B | 58 | 54 |  |
|  | 18 | 1OB5 | D | 58 | 54 |  |
|  | 19 | 1OB5 | F | 58 | 54 |  |
| **m^6^_6_A:G** | 1 | 1VQ6 | 4:0 | 76 | 2618 |  |
| **m^1^G:C** | 1 | 1YFG | A | 9 | 23 |  |
| **m^2^G:U cWW** | 1 | 1FIR | A | 6 | 67 |  |
| **m^2^G:C cWW** | 1 | 1EHZ | A | 10 | 25 |  |
|  | 2 | 1EVV | A | 10 | 25 |  |
|  | 3 | 1F7U | B | 910 | 925 |  |
|  | 4 | 1F7V | B | 910 | 925 |  |
|  | 5 | 1FIR | A | 10 | 25 |  |
|  | 6 | 1OB2 | B | 10 | 25 |  |
|  | 7 | 1TN1 | A | 10 | 25 |  |
|  | 8 | 1TN2 | A | 10 | 25 |  |
|  | 9 | 1TRA | A | 10 | 25 |  |
|  | 10 | 1TTT | D | 10 | 25 |  |
|  | 11 | 1TTT | F | 10 | 25 |  |
|  | 12 | 1YFG | A | 510 | 525 |  |
|  | 13 | 2DLC | Y | 510 | 525 |  |
|  | 14 | 4DR2 | A | 1207 | 1051 |  |
|  | 15 | 4DR3 | A | 1207 | 1051 |  |
|  | 16 | 4DR5 | A | 1207 | 1051 |  |
|  | 17 | 4DR6 | A | 1207 | 1051 |  |
|  | 18 | 4DUY | A | 1207 | 1051 |  |
|  | 19 | 4DV6 | A | 1207 | 1051 |  |
|  | 20 | 4DV7 | A | 1207 | 1051 |  |
|  | 21 | 4TNA | A | 10 | 25 |  |
|  | 22 | 4TRA | A | 10 | 25 |  |
|  | 23 | 6TNA | A | 10 | 25 |  |
| **m^2^G:C cWW** | 1 | 1OB5 | B | 10 | 25 |  |
|  | 2 | 1OB5 | D | 10 | 25 |  |
|  | 3 | 1OB5 | F | 10 | 25 |  |
| **m^2^_2_G:A cWW** | 1 | 1EHZ | A | 26 | 44 |  |
|  | 2 | 1EVV | A | 26 | 44 |  |
|  | 3 | 1OB2 | B | 26 | 44 |  |
|  | 4 | 1OB5 | D | 26 | 44 |  |
|  | 5 | 1OB5 | F | 26 | 44 |  |
|  | 6 | 1OB5 | B | 26 | 44 |  |
|  | 7 | 1TN1 | A | 26 | 44 |  |
|  | 8 | 1TN2 | A | 26 | 44 |  |
|  | 9 | 1TRA | A | 26 | 44 |  |
|  | 10 | 1TTT | D | 26 | 44 |  |
|  | 11 | 1TTT | E | 26 | 44 |  |
|  | 12 | 1YFG | A | 26 | 44 |  |
|  | 13 | 2DLC | Y | 526 | 544 |  |
|  | 14 | 3CJZ | A | 19 | 7 |  |
|  | 15 | 3CJZ | B | 6 | 20 |  |
|  | 16 | 4TNA | A | 26 | 44 |  |
|  | 17 | 4TRA | A | 26 | 44 |  |
|  | 18 | 6TNA | A | 26 | 44 |  |
|  | 19 | 1F7V | B | 926 | 944 |  |
|  | 20 | 1TRA | A | 26 | 44 |  |
| **m^7^G:G tWH** | 1 | 1EHZ | A | 46 | 22 |  |
|  | 2 | 1EVV | A | 46 | 22 |  |
|  | 3 | 1FIR | A | 46 | 22 |  |
|  | 4 | 1OB2 | B | 46 | 22 |  |
|  | 5 | 1OB5 | B | 46 | 22 |  |
|  | 6 | 1OB5 | D | 46 | 22 |  |
|  | 7 | 1OB5 | F | 46 | 22 |  |
|  | 8 | 1TN1 | A | 46 | 22 |  |
|  | 9 | 1TN2 | A | 46 | 22 |  |
|  | 10 | 1TRA | A | 46 | 22 |  |
|  | 11 | 1TTT | D | 46 | 22 |  |
|  | 12 | 1TTT | E | 46 | 22 |  |
|  | 13 | 1TTT | F | 46 | 22 |  |
|  | 14 | 1YFG | A | 46 | 22 |  |
|  | 15 | 2Y0U | Y | 46 | 22 |  |
|  | 16 | 2Y0W | Y | 46 | 22 |  |
|  | 17 | 2Y10 | Y | 46 | 22 |  |
|  | 18 | 2Y14 | Y | 46 | 22 |  |
|  | 19 | 2Y16 | Y | 46 | 22 |  |
|  | 20 | 2Y18 | Y | 46 | 22 |  |
|  | 21 | 4TNA | A | 46 | 22 |  |
|  | 22 | 4TRA | A | 46 | 22 |  |
|  | 23 | 6TNA | A | 46 | 22 |  |
|  | 24 | 1C0A | B | 46 | 22 |  |
|  | 25 | 1EFW | C | 46 | 22 |  |
|  | 26 | 1EFW | D | 46 | 22 |  |
|  | 27 | 1QF6 | B | 46 | 22 |  |
| **m^7^G:C cWW** | 1 | 4DR2 | A | 527 | 522 |  |
|  | 2 | 4DR3 | A | 527 | 522 |  |
|  | 3 | 4DR5 | A | 527 | 522 |  |
|  | 4 | 4DR6 | A | 527 | 522 |  |
|  | 5 | 4DUY | A | 527 | 522 |  |
|  | 6 | 4DV6 | A | 527 | 522 |  |
|  | 7 | 4DV7 | A | 527 | 522 |  |
| **m^5^C:G cWW** | 1 | 1ASY | R | 649 | 665 |  |
|  | 2 | 1ASY | S | 649 | 665 |  |
|  | 3 | 1ASZ | R | 649 | 665 |  |
|  | 4 | 1ASZ | S | 649 | 665 |  |
|  | 5 | 1EHZ | A | 40 | 30 |  |
|  | 6 | 1EHZ | A | 49 | 65 |  |
|  | 7 | 1EVV | A | 40 | 30 |  |
|  | 8 | 1EVV | A | 49 | 65 |  |
|  | 9 | 1F7U | B | 949 | 965 |  |
|  | 10 | 1F7V | B | 949 | 965 |  |
|  | 11 | 1FIR | A | 949 | 965 |  |
|  | 12 | 1IL2 | C | 949 | 965 |  |
|  | 13 | 1IL2 | D | 1949 | 1965 |  |
|  | 14 | 1OB2 | B | 40 | 30 |  |
|  | 15 | 1OB2 | B | 49 | 65 |  |
|  | 16 | 1OB5 | B | 49 | 65 |  |
|  | 17 | 1OB5 | B | 40 | 30 |  |
|  | 18 | 1OB5 | D | 49 | 65 |  |
|  | 19 | 1OB5 | D | 40 | 30 |  |
|  | 20 | 1OB5 | F | 49 | 65 |  |
|  | 21 | 1OB5 | F | 40 | 30 |  |
|  | 22 | 1TN1 | A | 49 | 65 |  |
|  | 23 | 1TN1 | A | 40 | 30 |  |
|  | 24 | 1TN2 | A | 49 | 65 |  |
|  | 25 | 1TN2 | A | 40 | 30 |  |
|  | 26 | 1TRA | A | 49 | 65 |  |
|  | 27 | 1TRA | A | 40 | 30 |  |
|  | 28 | 1TTT | D | 49 | 65 |  |
|  | 29 | 1TTT | D | 40 | 30 |  |
|  | 30 | 1TTT | E | 49 | 65 |  |
|  | 31 | 1TTT | E | 40 | 30 |  |
|  | 32 | 1TTT | F | 49 | 65 |  |
|  | 33 | 1TTT | F | 40 | 30 |  |
|  | 34 | 1VTQ | A | 48 | 64 |  |
|  | 35 | 1YFG | A | 49 | 65 |  |
|  | 36 | 2TRA | A | 48 | 64 |  |
|  | 37 | 3TRA | A | 48 | 64 |  |
|  | 38 | 4DR2 | A | 1404 | 1497 |  |
|  | 39 | 4DR2 | A | 1407 | 1494 |  |
|  | 40 | 4DR3 | A | 1404 | 1497 |  |
|  | 41 | 4DR3 | A | 1407 | 1494 |  |
|  | 42 | 4DR5 | A | 1404 | 1497 |  |
|  | 43 | 4DR5 | A | 1407 | 1494 |  |
|  | 44 | 4DR6 | A | 1404 | 1497 |  |
|  | 45 | 4DR6 | A | 1407 | 1494 |  |
|  | 46 | 4DUY | A | 1404 | 1497 |  |
|  | 47 | 4DUY | A | 1407 | 1494 |  |
|  | 48 | 4DV6 | A | 1404 | 1497 |  |
|  | 49 | 4DV6 | A | 1407 | 1494 |  |
|  | 50 | 4DV7 | A | 1404 | 1497 |  |
|  | 51 | 4DV7 | A | 1407 | 1494 |  |
|  | 52 | 4TNA | A | 40 | 30 |  |
|  | 53 | 4TNA | A | 49 | 65 |  |
|  | 54 | 4TRA | A | 40 | 30 |  |
|  | 55 | 4TRA | A | 49 | 65 |  |
|  | 56 | 6TNA | A | 40 | 30 |  |
|  | 57 | 6TNA | A | 49 | 65 |  |
| **m^5^C:G tWW** | 1 | 1YFG | A | 48 | 15 |  |
|  | 2 | 2DLC | Y | 548 | 515 |  |
|  | 3 | 1FIR | A | 48 | 15 |  |
| **m^5^U:m^1^A tWH** | 1 | 1EHZ | A | 58 | 54 |  |
|  | 2 | 1EVV | A | 58 | 54 |  |
|  | 3 | 1F7U | B | 58 | 54 |  |
|  | 4 | 1F7V | B | 58 | 54 |  |
|  | 5 | 1FIR | A | 58 | 54 |  |
|  | 6 | 1TN1 | A | 58 | 54 |  |
|  | 7 | 1TN2 | A | 58 | 54 |  |
|  | 8 | 1TRA | A | 58 | 54 |  |
|  | 9 | 1TTT | D | 58 | 54 |  |
|  | 10 | 1TTT | E | 58 | 54 |  |
|  | 11 | 1TTT | F | 58 | 54 |  |
|  | 12 | 2DLC | Y | 58 | 54 |  |
|  | 13 | 4TNA | A | 58 | 54 |  |
|  | 14 | 4TRA | A | 58 | 54 |  |
|  | 15 | 6TNA | A | 58 | 54 |  |
|  | 16 | 1H3E | B | 58 | 54 |  |
|  | 17 | 1OB5 | B | 58 | 54 |  |
|  | 18 | 1OB5 | D | 58 | 54 |  |
|  | 19 | 1OB5 | F | 58 | 54 |  |
| **m^5^U:A tWH** | 1 | 1ASY | R | 654 | 658 |  |
|  | 2 | 1ASY | S | 654 | 658 |  |
|  | 3 | 1ASZ | R | 654 | 658 |  |
|  | 4 | 1ASZ | S | 654 | 658 |  |
|  | 5 | 1B23 | R | 54 | 58 |  |
|  | 6 | 1C0A | B | 654 | 658 |  |
|  | 7 | 1EFW | C | 54 | 58 |  |
|  | 8 | 1EFW | D | 54 | 58 |  |
|  | 9 | 1I9V | A | 54 | 58 |  |
|  | 10 | 1IL2 | C | 954 | 958 |  |
|  | 11 | 1IL2 | D | 1954 | 1958 |  |
|  | 12 | 1QF6 | B | 54 | 58 |  |
|  | 13 | 1SER | T | 54 | 58 |  |
|  | 14 | 1VTQ | A | 54 | 58 |  |
|  | 15 | 2CZJ | B | 54 | 58 |  |
|  | 16 | 2CZJ | F | 54 | 58 |  |
|  | 17 | 2FMT | C | 54 | 58 |  |
|  | 18 | 2FMT | D | 54 | 58 |  |
|  | 19 | 2J00 | V | 54 | 58 |  |
|  | 20 | 2J02 | V | 54 | 58 |  |
|  | 21 | 2TRA | A | 53 | 57 |  |
|  | 22 | 2V46 | W | 54 | 58 |  |
|  | 23 | 2WDH | V | 54 | 58 |  |
|  | 24 | 2Y0U | Y | 54 | 58 |  |
|  | 25 | 2Y0W | Y | 54 | 58 |  |
|  | 26 | 2Y10 | Y | 54 | 58 |  |
|  | 27 | 2Y18 | Y | 54 | 58 |  |
|  | 28 | 3CW5 | A | 54 | 58 |  |
|  | 29 | 3CW6 | A | 54 | 58 |  |
|  | 30 | 3KFU | K | 54 | 58 |  |
|  | 31 | 3KFU | L | 54 | 58 |  |
|  | 32 | 3KFU | M | 54 | 58 |  |
|  | 33 | 3KFU | N | 54 | 58 |  |
|  | 34 | 3KIQ | V | 54 | 58 |  |
|  | 35 | 3KIS | V | 54 | 58 |  |
|  | 36 | 3TRA | A | 53 | 57 |  |
|  | 37 | 4BYB | V | 54 | 58 |  |
|  | 38 | 4JYZ | B | 954 | 958 |  |
| **m^5^U:G tWH** | 1 | 1H4Q | T | 54 | 58 |  |
|  | 2 | 1H4S | T | 54 | 58 |  |
| **m^5^U:G cWW** | 1 | 1U6B | D,B | 1 | 10 |  |
|  | 2 | 1ZZN | D,B | 1 | 10 |  |
| **s^4^U:A tWH** | 1 | 1B23 | R | 8 | 14 |  |
|  | 2 | 1C0A | B | 608 | 614 |  |
|  | 3 | 1EFW | C | 8 | 14 |  |
|  | 4 | 1EFW | D | 8 | 14 |  |
|  | 5 | 2FMT | C | 8 | 14 |  |
|  | 6 | 2FMT | D | 8 | 14 |  |
|  | 7 | 2XQD | Y | 8 | 14 |  |
|  | 8 | 2Y0U | Y | 8 | 14 |  |
|  | 9 | 2Y0W | Y | 8 | 14 |  |
|  | 10 | 2Y0Y | Y | 8 | 14 |  |
|  | 11 | 2Y12 | Y | 8 | 14 |  |
|  | 12 | 2Y14 | Y | 8 | 14 |  |
|  | 13 | 2Y14 | Y | 8 | 14 |  |
|  | 14 | 2Y16 | Y | 8 | 14 |  |
|  | 15 | 2Y16 | Y | 8 | 14 |  |
|  | 16 | 2Y18 | Y | 8 | 14 |  |
|  | 17 | 3CW5 | A | 8 | 14 |  |
|  | 18 | 3CW5 | A | 8 | 14 |  |
|  | 19 | 3CW6 | A | 8 | 14 |  |
|  | 20 | 3CW6 | A | 8 | 14 |  |
|  | 21 | 4JYZ | B | 908 | 914 |  |
| **H2U:U tWW** | 1 | 1ASY | S | 616 | 659 |  |
|  | 2 | 1ASZ | R | 616 | 659 |  |
|  | 3 | 1ASZ | S | 616 | 659 |  |
|  | 4 | 1IL2 | C | 916 | 959 |  |
|  | 5 | 1IL2 | D | 1916 | 1959 |  |
|  | 6 | 1OB2 | B | 16 | 59 |  |
|  | 7 | 1OB5 | B | 16 | 59 |  |
|  | 8 | 1OB5 | D | 16 | 59 |  |
|  | 9 | 1OB5 | F | 16 | 59 |  |
|  | 10 | 1TTT | D | 16 | 59 |  |
|  | 11 | 1TTT | E | 16 | 59 |  |
|  | 12 | 1TTT | F | 16 | 59 |  |
| **H2U:G tWS** | 1 | 1SER | T | 20 | 15 |  |
| **H2U:G cHS** | 1 | 1C0A | B | 620 | 619 |  |
| **Ψ:A cWW** | 1 | 1B23 | R | 39 | 31 |  |
|  | 2 | 1EVV | A | 39 | 31 |  |
|  | 3 | 1F7U | B | 901 | 972 |  |
|  | 4 | 1F7U | B | 927 | 943 |  |
|  | 5 | 1F7V | B | 901 | 972 |  |
|  | 6 | 1F7V | B | 927 | 943 |  |
|  | 7 | 1FIR | A | 27 | 43 |  |
|  | 8 | 1FIR | A | 39 | 31 |  |
|  | 9 | 1OB5 | D | 39 | 31 |  |
|  | 10 | 1OB5 | F | 39 | 31 |  |
|  | 11 | 1TN1 | A | 39 | 31 |  |
|  | 12 | 1TN2 | A | 39 | 31 |  |
|  | 13 | 1TRA | A | 39 | 31 |  |
|  | 14 | 1TTT | E | 39 | 31 |  |
|  | 15 | 1TTT | F | 39 | 31 |  |
|  | 16 | 3CGP | A | 6 | 21 |  |
|  | 17 | 3CGQ | B | 6 | 21 |  |
|  | 18 | 3CGR | B | 7 | 19 |  |
|  | 19 | 4JV5 | Y | 4 | 36 |  |
|  | 20 | 4JYA | Y | 4 | 36 |  |
|  | 21 | 4K0L | X | 19 | 36 |  |
|  | 22 | 4TNA | A | 39 | 31 |  |
|  | 23 | 4TRA | A | 39 | 31 |  |
|  | 24 | 6TNA | A | 39 | 31 |  |
| **Ψ:A cHW** | 1 | 1TTT | D | 39 | 31 |  |
| **Ψ:U tWW** | 1 | 1S72 | 0 | 2621 | 1838 |  |
|  | 2 | 1VQ4 | 0 | 2621 | 1838 |  |
|  | 3 | 1VQ5 | 0 | 2621 | 1838 |  |
|  | 4 | 1VQ6 | 0 | 2621 | 1838 |  |
|  | 5 | 1VQ7 | 0 | 2621 | 1838 |  |
|  | 6 | 1VQ8 | 0 | 2621 | 1838 |  |
|  | 7 | 1VQ9 | 0 | 2621 | 1838 |  |
|  | 8 | 1VQK | 0 | 2621 | 1838 |  |
|  | 9 | 1VQL | 0 | 2621 | 1838 |  |
|  | 10 | 1VQM | 0 | 2621 | 1838 |  |
|  | 11 | 1VQN | 0 | 2621 | 1838 |  |
|  | 12 | 1VQO | 0 | 2621 | 1838 |  |
|  | 13 | 1VQP | 0 | 2621 | 1838 |  |
|  | 14 | 1YHQ | 0 | 2621 | 1838 |  |
|  | 15 | 1Y12 | 0 | 2621 | 1838 |  |
|  | 16 | 1YIJ | 0 | 2621 | 1838 |  |
|  | 17 | 1YIT | 0 | 2621 | 1838 |  |
|  | 18 | 1YJ9 | 0 | 2621 | 1838 |  |
|  | 19 | 1YJN | 0 | 2621 | 1838 |  |
|  | 20 | 1YJW | 0 | 2621 | 1838 |  |
|  | 21 | 2OTJ | 0 | 2621 | 1838 |  |
|  | 22 | 2OTL | 0 | 2621 | 1838 |  |
|  | 23 | 2QA4 | 0 | 2621 | 1838 |  |
|  | 24 | 2QEX | 0 | 2621 | 1838 |  |
|  | 25 | 3CC2 | 0 | 2621 | 1838 |  |
|  | 26 | 3CC4 | 0 | 2621 | 1838 |  |
|  | 27 | 3CC7 | 0 | 2621 | 1838 |  |
|  | 28 | 3CCE | 0 | 2621 | 1838 |  |
|  | 29 | 3CCJ | 0 | 2621 | 1838 |  |
|  | 30 | 3CCL | 0 | 2621 | 1838 |  |
|  | 31 | 3CCM | 0 | 2621 | 1838 |  |
|  | 32 | 3CCQ | 0 | 2621 | 1838 |  |
|  | 33 | 3CCR | 0 | 2621 | 1838 |  |
|  | 34 | 3CCS | 0 | 2621 | 1838 |  |
|  | 35 | 3CCU | 0 | 2621 | 1838 |  |
|  | 36 | 3CCV | 0 | 2621 | 1838 |  |
|  | 37 | 3CD6 | 0 | 2621 | 1838 |  |
|  | 38 | 3CMA | 0 | 2621 | 1838 |  |
|  | 39 | 3CME | 0 | 2621 | 1838 |  |
|  | 40 | 3G4S | 0 | 2621 | 1838 |  |
|  | 41 | 3G6E | 0 | 2621 | 1838 |  |
|  | 42 | 3G71 | 0 | 2621 | 1838 |  |
|  | 43 | 3I55 | 0 | 2621 | 1838 |  |
|  | 44 | 3I56 | 0 | 2621 | 1838 |  |
|  | 45 | 4HUB | 0 | 2621 | 1838 |  |
| **Ψ:G tBs/Ww** | 1 | 1ASY | R | 655 | 617 |  |
|  | 2 | 1H3E | B | 55 | 18 |  |
|  | 3 | 1IL2 | C | 955 | 917 |  |
|  | 4 | 1VTQ | A | 54 | 17 |  |
|  | 5 | 2CZJ | B | 55 | 12 |  |
|  | 6 | 1ASY | S | 655 | 617 |  |
|  | 7 | 1ASZ | S | 655 | 617 |  |
|  | 8 | 1ASZ | R | 655 | 617 |  |
|  | 9 | 1B23 | R | 55 | 18 |  |
|  | 10 | 1C0A | B | 55 | 18 |  |
|  | 11 | 1EFW | C | 55 | 18 |  |
|  | 12 | 1EFW | D | 55 | 18 |  |
|  | 13 | 1EHZ | A | 55 | 18 |  |
|  | 14 | 1EVV | A | 55 | 18 |  |
|  | 15 | 1F7U | B | 955 | 917 |  |
|  | 16 | 1F7V | B | 955 | 917 |  |
|  | 17 | 1FIR | A | 55 | 18 |  |
|  | 18 | 1H4Q | T | 55 | 18 |  |
|  | 19 | 1H4S | T | 55 | 18 |  |
|  | 20 | 1IL2 | D | 1955 | 1917 |  |
|  | 21 | 1OB2 | B | 55 | 18 |  |
|  | 22 | 1OB5 | B | 55 | 18 |  |
|  | 23 | 1OB5 | D | 55 | 18 |  |
|  | 24 | 1OB5 | F | 55 | 18 |  |
|  | 25 | 1QF6 | B | 55 | 18 |  |
|  | 26 | 1SER | T | 55 | 18 |  |
|  | 27 | 1TN1 | A | 55 | 18 |  |
|  | 28 | 1TN2 | A | 55 | 18 |  |
|  | 29 | 1TRA | A | 55 | 18 |  |
|  | 30 | 1TTT | D | 55 | 18 |  |
|  | 31 | 1TTT | E | 55 | 18 |  |
|  | 32 | 1TTT | F | 55 | 18 |  |
|  | 33 | 2FMT | D | 55 | 18 |  |
|  | 34 | 2TRA | A | 54 | 17 |  |
|  | 35 | 2Y14 | Y | 55 | 18 |  |
|  | 36 | 2Y16 | Y | 55 | 18 |  |
|  | 37 | 3CW5 | A | 55 | 18 |  |
|  | 38 | 3CW6 | A | 55 | 18 |  |
|  | 39 | 3KFU | N | 55 | 18 |  |
|  | 40 | 3KFU | K | 55 | 18 |  |
|  | 41 | 3KFU | L | 55 | 18 |  |
|  | 42 | 3KFU | M | 55 | 18 |  |
|  | 43 | 3TRA | A | 54 | 17 |  |
|  | 44 | 4TNA | A | 55 | 18 |  |
|  | 45 | 4TRA | A | 55 | 18 |  |
|  | 46 | 6TNA | A | 55 | 18 |  |
|  | 47 | 2DLC | Y | 555 | 518 |  |
|  | 48 | 4JYZ | B | 955 | 918 |  |
| **Ψ:G cWW** | 1 | 1ASY | R | 613 | 622 |  |
|  | 2 | 1ASY | S | 613 | 622 |  |
|  | 3 | 1ASZ | R | 613 | 622 |  |
|  | 4 | 1ASZ | S | 613 | 622 |  |
|  | 5 | 1C0A | B | 665 | 649 |  |
|  | 6 | 1EFW | C | 65 | 49 |  |
|  | 7 | 1EFW | D | 65 | 49 |  |
|  | 8 | 1IL2 | C | 913 | 922 |  |
|  | 9 | 1IL2 | D | 1913 | 1922 |  |
|  | 10 | 1N33 | Y | 40 | 30 |  |
|  | 11 | 1VTQ | A | 13 | 22 |  |
|  | 12 | 2TRA | A | 13 | 22 |  |
|  | 13 | 3CGS | B | 6 | 20 |  |
|  | 14 | 3TRA | A | 13 | 22 |  |
| **Ψ:C cSW** | 1 | 4DR3 | A | 516 | 519 |  |
|  | 2 | 4DR5 | A | 516 | 519 |  |
|  | 3 | 4DR6 | A | 516 | 519 |  |
|  | 4 | 4DUY | A | 516 | 519 |  |
|  | 5 | 4DV7 | A | 516 | 519 |  |
|  | 6 | 4DV6 | A | 516 | 519 |  |
|  | 7 | 4DR2 | A | 516 | 519 |  |
|  |  |  |  |  |  |  |
|  |  |  |  |  |  |  |
| **5BrC:G cWW** | 1 | 1LNT | A | 22 | 3 |  |
|  | 2 | 1LNT | B | 11 | 14 |  |
|  | 3 | 1QBP | A | 18 | 14 |  |
|  | 4 | 1LNT | B | 22 | 3 |  |
|  | 5 | 1QBP | A | 3 | 29 |  |
|  | 6 | 1QBP | A | 4 | 28 |  |
|  | 7 | 1QBP | B | 19 | 13 |  |
|  | 8 | 1QBP | C | 33 | 59 |  |
|  | 9 | 1QBP | C | 34 | 58 |  |
|  | 10 | 1QBP | D | 48 | 44 |  |
|  | 11 | 1QBP | D | 49 | 43 |  |
|  | 12 | 1QBP | E | 63 | 89 |  |
|  | 13 | 1QBP | E | 64 | 88 |  |
|  | 14 | 1QBP | F | 78 | 74 |  |
|  | 15 | 1QBP | F | 79 | 73 |  |
|  | 16 | 430D | A | 27 | 3 |  |
| **5BrU:A cWW** | 1 | 1F1T | A | 36 | 3 |  |
|  | 2 | 1F1T | A | 35 | 4 |  |
|  | 3 | 1F1T | A | 11 | 22 |  |
|  | 4 | 1JID | A | 137 | 160 |  |
|  | 5 | 1JZV | A | 7 | 10 |  |
|  | 6 | 1JZV | A | 27 | 30 |  |
|  | 7 | 1KH6 | A | 36 | 25 |  |
|  | 8 | 1KH6 | A | 37 | 6 |  |
|  | 9 | 1KH6 | A | 38 | 7 |  |
|  | 10 | 1RLG | C | 5 | 21 |  |
|  | 11 | 1RLG | D | 5 | 21 |  |
|  | 12 | 1Y3O | A | 3 | 21 |  |
|  | 13 | 1Y3O | B | 3 | 21 |  |
|  | 14 | 1Y3S | A | 3 | 21 |  |
|  | 15 | 1Y3S | B | 3 | 21 |  |
|  | 16 | 1Y90 | A | 2 | 22 |  |
|  | 17 | 1Y90 | B | 2 | 22 |  |
|  | 18 | 1YXP | A | 3 | 21 |  |
|  | 19 | 1YXP | A | 3 | 21 |  |
|  | 20 | 1YXP | B | 3 | 21 |  |
|  | 21 | 1YXP | B | 3 | 21 |  |
|  | 22 | 1ZCI | A | 2 | 22 |  |
|  | 23 | 1ZCI | B | 2 | 22 |  |
|  | 24 | 1ZCI | C | 2 | 22 |  |
|  | 25 | 1ZCI | D | 2 | 22 |  |
|  | 26 | 2ANR | B | 202 | 224 |  |
|  | 27 | 2AZ0 | C | 16 | 3 |  |
|  | 28 | 2AZ0 | C | 4 | 15 |  |
|  | 29 | 2AZ0 | C | 12 | 7 |  |
|  | 30 | 2AZ0 | D | 16 | 3 |  |
|  | 31 | 2AZ0 | D | 4 | 15 |  |
|  | 32 | 2AZ0 | D | 12 | 7 |  |
|  | 33 | 2AZ2 | C | 16 | 3 |  |
|  | 34 | 2AZ2 | C | 4 | 15 |  |
|  | 35 | 2AZ2 | C | 12 | 7 |  |
|  | 36 | 2AZ2 | D | 16 | 3 |  |
|  | 37 | 2AZ2 | D | 4 | 15 |  |
|  | 38 | 2AZ2 | D | 12 | 7 |  |
|  | 39 | 2FCX | A | 3 | 21 |  |
|  | 40 | 2FCX | B | 3 | 21 |  |
|  | 41 | 2FCY | A | 3 | 21 |  |
|  | 42 | 2FCY | B | 3 | 21 |  |
|  | 43 | 2FCZ | A | 3 | 21 |  |
|  | 44 | 2FCZ | B | 3 | 21 |  |
|  | 45 | 2FCZ | C | 3 | 21 |  |
|  | 46 | 2FCZ | D | 3 | 21 |  |
|  | 47 | 2FD0 | A | 3 | 21 |  |
|  | 48 | 2FD0 | B | 3 | 21 |  |
|  | 49 | 2JLT | A | 3 | 16 |  |
|  | 50 | 2JLT | B | 14 | 3 |  |
|  | 51 | 2OEU | A,B | 11 | 10 |  |
|  | 52 | 2PN3 | A,B | 113 | 50 |  |
|  | 53 | 2PN3 | A,B | 101 | 66 |  |
|  | 54 | 2PN4 | A | 113 | 50 |  |
|  | 55 | 2PN4 | B | 101 | 66 |  |
|  | 56 | 2PN4 | C | 113 | 50 |  |
|  | 57 | 2PN4 | D | 101 | 66 |  |
|  | 58 | 2R1S | A,B | 6 | 21 |  |
|  | 59 | 2R1S | A,B | 8 | 19 |  |
|  | 60 | 2R20 | A,B | 6 | 21 |  |
|  | 61 | 2R20 | A,B | 8 | 19 |  |
|  | 62 | 2R21 | A,B | 6 | 21 |  |
|  | 63 | 2R21 | A,B | 8 | 19 |  |
|  | 64 | 3BNO | A | 9 | 12 |  |
|  | 65 | 3BNO | B | 9 | 12 |  |
|  | 66 | 3BNO | C | 9 | 12 |  |
|  | 67 | 3BNO | D | 9 | 12 |  |
|  | 68 | 3BNQ | A | 11 | 14 |  |
|  | 69 | 3BNQ | B | 11 | 14 |  |
|  | 70 | 3BNQ | C | 11 | 14 |  |
|  | 71 | 3BNQ | D | 11 | 14 |  |
|  | 72 | 3BNR | A | 11 | 13 |  |
|  | 73 | 3BNR | B | 10 | 14 |  |
|  | 74 | 3BNR | C | 10 | 14 |  |
|  | 75 | 3BNR | D | 11 | 13 |  |
|  | 76 | 3BNS | A | 11 | 13 |  |
|  | 77 | 3BNS | B | 10 | 14 |  |
|  | 78 | 3BNS | C | 10 | 14 |  |
|  | 79 | 3BNS | D | 11 | 13 |  |
|  | 80 | 3LOA | A | 1487 | 1413 |  |
|  | 81 | 3LOA | B | 1487 | 1413 |  |
|  | 82 | 3LOA | B | 1406 | 1495 |  |
|  | 83 | 3MEI | A | 12 | 11 |  |
|  | 84 | 3MEI | B | 12 | 11 |  |
|  | 85 | 3P59 | A,B | 59 | 109 |  |
|  | 86 | 3P59 | E,F | 109 | 59 |  |
|  | 87 | 3P59 | G,H | 59 | 109 |  |
|  | 88 | 3S2D | R,T | 9 | 21 |  |
|  | 89 | 3TD0 | A,B | 11 | 37 |  |
|  | 90 | 3TD0 | B,A | 34 | 14 |  |
|  | 91 | 3TD1 | A,B | 11 | 37 |  |
|  | 92 | 3TD1 | B,A | 34 | 14 |  |
|  | 93 | 3ZD5 | A,B | 18 | 3 |  |
|  | 94 | 3ZD5 | B,A | 11 | 10 |  |
|  | 95 | 406D | A | 12 | 19 |  |
| **5BrU:G cWW** | 1 | 2AO5 | A | 5 | 6 |  |
|  | 2 | 2AO5 | B | 5 | 6 |  |
|  | 3 | 2AO5 | A | 5 | 6 |  |
|  | 4 | 1JID | B | 142 | 155 |  |
|  | 5 | 406D | A | 12 | 23 |  |
|  | 6 | 406D | E | 29 | 6 |  |
|  | 7 | 406D | E | 12 | 23 |  |
|  | 8 | 2A05 | A | 5 | 6 |  |
|  | 9 | 406D | A | 29 | 6 |  |
|  | 10 | 406D | E | 12 | 27 |  |
|  | 11 | 406D | E | 29 | 10 |  |
|  | 12 | 406D | A | 29 | 6 |  |
|  | 13 | 406D | A | 29 | 6 |  |
|  | 14 | 406D | E | 12 | 23 |  |
|  | 15 | 406D | A | 12 | 27 |  |
|  | 16 | 406D | A | 10 | 29 |  |
| **5BrU:5BrU cWW** | 1 | 1RLG | C | 5 | 21 |  |
|  | 2 | 1RLG | D | 5 | 21 |  |
| **5BrU:A cSH** | 1 | 1KH6 | A | 37 | 6 |  |
| **5BrU:A cWH** | 1 | 1KH6 | A | 38 | 7 |  |
| **5BrU:A cWS** | 1 | 1F1T | A | 11 | 26 |  |
| **IU:A cWW** | 1 | 1M5K | A | 17 | 5 |  |
|  | 2 | 1M5K | D | 17 | 5 |  |
|  | 3 | 1M5K | E | 17 | 5 |  |
|  | 4 | 1YVP | C | 3 | 7 |  |
|  | 5 | 1YVP | D | 3 | 7 |  |
|  | 6 | 1YVP | E | 3 | 7 |  |
|  | 7 | 1YVP | F | 3 | 7 |  |
|  | 8 | 464D | A | 2 | 13 |  |
|  | 9 | 464D | B | 2 | 13 |  |
|  | 10 | 464D | C | 2 | 13 |  |
|  | 11 | 464D | D | 2 | 13 |  |
|  | 12 | 2H0S | A | 12 | 29 |  |
|  | 13 | 2H0S | B | 12 | 29 |  |
| **IU:G cWW** | 1 | 1YVP | C | 6 | 4 |  |
|  | 2 | 1YVP | D | 6 | 4 |  |
|  | 3 | 1YVP | E | 6 | 4 |  |
|  | 4 | 1YVP | F | 6 | 4 |  |

**Table S2.** Complete list of the occurrences in PDB structures of modified bases never involved in H-bonding interactions with other bases.

| **Base pair** | **Occurence** | **PDB ID** | **Chain ID** | **Chain ID** | **Res ID** |
| --- | --- | --- | --- | --- | --- |
| **2-methyladenine (m^2^A)** | 1 | 1EFW | 37 | C | 37 |
|  | 2 | 1EFW | 37 | D | 37 |
|  | 3 | 1GSG | 37 | T | 37 |
|  | 4 | 4JYZ | 937 | B | 937 |
| **2-methylthio-N6-isopentenyladenine (ms^2^i^6^A)** | 1 | 1B23 | 37 | R | 37 |
|  | 2 | 2XQD | 37 | Y | 37 |
|  | 3 | 2Y0U | 37 | Y | 37 |
|  | 4 | 2Y0Y | 37 | Y | 37 |
|  | 5 | 2Y10 | 37 | Y | 37 |
|  | 6 | 2Y12 | 37 | Y | 37 |
|  | 7 | 2Y14 | 37 | Y | 37 |
|  | 8 | 2Y16 | 37 | Y | 37 |
|  | 9 | 2Y18 | 37 | Y | 37 |
|  | 10 | 3I8G | 37 | D | 37 |
|  | 11 | 3I8G | 37 | B | 37 |
|  | 12 | 3I8G | 37 | C | 37 |
|  | 13 | 3I8H | 37 | D | 37 |
|  | 14 | 3I8H | 37 | B | 37 |
|  | 15 | 3I8H | 37 | C | 37 |
|  | 16 | 3UZ6 | 38 | B | 38 |
|  | 17 | 3UZ6 | 38 | D | 38 |
|  | 18 | 3UZ7 | 38 | B | 38 |
|  | 19 | 3UZ7 | 38 | D | 38 |
|  | 20 | 3UZG | 38 | B | 38 |
|  | 21 | 3UZG | 38 | D | 38 |
|  | 22 | 3UZI | 38 | B | 38 |
|  | 23 | 3UZI | 38 | D | 38 |
|  | 24 | 3UZL | 38 | B | 38 |
|  | 25 | 3UZL | 38 | D | 38 |
|  | 26 | 3UZM | 38 | B | 38 |
|  | 27 | 3UZM | 38 | D | 38 |
|  | 28 | 2Y0W | 37 | Y | 37 |
| **2-methylthio-N6-threonylcarbamoyladenine (ms^2^t^6^ A)** | 1 | 1FIR | 37 | A | 37 |
|  | 2 | 3T1H | 37 | X | 37 |
|  | 3 | 3T1Y | 37 | X | 37 |
| **N6-threonylcarbamoyladenine (t^6^A)** | 1 | 1XM0 | 37 | X | 37 |
|  | 2 | 1XMQ | 37 | X | 37 |
|  | 3 | 1YFG | 37 | A | 37 |
| Wybutosine (yW) | 1 | 1EHZ | 37 | A | 37 |
|  | 2 | 1TTT | 37 | D | 37 |
|  | 3 | 1TTT | 37 | E | 37 |
|  | 4 | 1TTT | 37 | F | 37 |
|  | 5 | 1EVV | 37 | A | 37 |
|  | 6 | 1I9V | 37 | A | 37 |
|  | 7 | 1OB2 | 37 | B | 37 |
|  | 8 | 1OB5 | 37 | B | 37 |
|  | 9 | 1OB5 | 37 | D | 37 |
|  | 10 | 1OB5 | 37 | F | 37 |
|  | 11 | 1TN1 | 37 | A | 37 |
|  | 12 | 1TN2 | 37 | A | 37 |
|  | 13 | 1TRA | 37 | A | 37 |
|  | 14 | 4TNA | 37 | A | 37 |
|  | 15 | 4TRA | 37 | A | 37 |
|  | 16 | 6TNA | 37 | A | 37 |
| **N7-4,5-cis-dihydroxy-1-cyclopentenyl-3-aminomethylguanine (QUO)** | 1 | 1C0A | 634 | B | 634 |
|  | 2 | 1EFW | 34 | C | 34 |
|  | 3 | 1EFW | 34 | D | 34 |
| **2-thiouracil (s^2^U)** | 1 | 2C4Y | 11 | R | 11 |
|  | 2 | 2C4Z | 11 | R | 11 |
|  | 3 | 2C4Y | 10 | S | 10 |
|  | 4 | 2C4Z | 10 | R | 10 |
|  | 5 | 2C4Z | 10 | S | 10 |
|  | 6 | 2C4Z | 10 | S | 10 |
|  |  |  |  |  |  |
| **5methoxycarbonylmethyl-2-thiouracil (mcm^5^s^2^U)** | 1 | 1FIR | 34 | A | 34 |
|  | 2 | 3T1H | 34 | X | 34 |
|  | 3 | 3T1Y | 34 | X | 34 |
| **3-methyluracil (m^3^U)** | 1 | 1S72 | 2619 | 0 | 2619 |
|  | 2 | 1VQ4 | 2619 | 0 | 2619 |
|  | 3 | 1VQ5 | 2619 | 0 | 2619 |
|  | 4 | 1VQ6 | 2619 | 0 | 2619 |
|  | 5 | 1VQ7 | 2619 | 0 | 2619 |
|  | 6 | 1VQ8 | 2619 | 0 | 2619 |
|  | 7 | 1VQ9 | 2619 | 0 | 2619 |
|  | 8 | 1VQK | 2619 | 0 | 2619 |
|  | 9 | 1VQL | 2619 | 0 | 2619 |
|  | 10 | 1VQM | 2619 | 0 | 2619 |
|  | 11 | 1VQN | 2619 | 0 | 2619 |
|  | 12 | 1VQO | 2619 | 0 | 2619 |
|  | 13 | 1VQP | 2619 | 0 | 2619 |
|  | 14 | 1YHQ | 2619 | 0 | 2619 |
|  | 15 | 1YI2 | 2619 | 0 | 2619 |
|  | 16 | 1YIJ | 2619 | 0 | 2619 |
|  | 17 | 1YIT | 2619 | 0 | 2619 |
|  | 18 | 1YJ9 | 2619 | 0 | 2619 |
|  | 19 | 1YJN | 2619 | 0 | 2619 |
|  | 20 | 1YJW | 2619 | 0 | 2619 |
|  | 21 | 2OTJ | 2619 | 0 | 2619 |
|  | 22 | 2OTL | 2619 | 0 | 2619 |
|  | 23 | 2QA4 | 2619 | 0 | 2619 |
|  | 24 | 2QEX | 2619 | 0 | 2619 |
|  | 25 | 3CC2 | 2619 | 0 | 2619 |
|  | 26 | 3CC4 | 2619 | 0 | 2619 |
|  | 27 | 3CC7 | 2619 | 0 | 2619 |
|  | 28 | 3CCE | 2619 | 0 | 2619 |
|  | 29 | 3CCJ | 2619 | 0 | 2619 |
|  | 30 | 3CCL | 2619 | 0 | 2619 |
|  | 31 | 3CCM | 2619 | 0 | 2619 |
|  | 32 | 3CCQ | 2619 | 0 | 2619 |
|  | 33 | 3CCR | 2619 | 0 | 2619 |
|  | 34 | 3CCS | 2619 | 0 | 2619 |
|  | 35 | 3CCU | 2619 | 0 | 2619 |
|  | 36 | 3CCV | 2619 | 0 | 2619 |
|  | 37 | 3CD6 | 2619 | 0 | 2619 |
|  | 38 | 3CMA | 2619 | 0 | 2619 |
|  | 39 | 3CME | 2619 | 0 | 2619 |
|  | 40 | 3G4S | 2619 | 0 | 2619 |
|  | 41 | 3G6E | 2619 | 0 | 2619 |
|  | 42 | 3G71 | 2619 | 0 | 2619 |
|  | 43 | 3I55 | 2619 | 0 | 2619 |
|  | 44 | 3I56 | 2619 | 0 | 2619 |
|  | 45 | 3LWQ | 10 | E | 10 |
|  | 46 | 4DR2 | 1498 | A | 1498 |
|  | 47 | 4DR3 | 1498 | A | 1498 |
|  | 48 | 4DR5 | 1498 | A | 1498 |
|  | 49 | 4DR6 | 1498 | A | 1498 |
|  | 50 | 4DUY | 1498 | A | 1498 |
|  | 51 | 4DV6 | 1498 | A | 1498 |
|  | 52 | 4DV7 | 1498 | A | 1498 |
|  | 53 | 4HUB | 2619 | 0 | 2619 |

**Table S3.** H-bond lengths of the optimized base pairs in the gas phase at the B3LYP level and RMSD values for the heavy atoms superimposition on the corresponding X-ray structure. MP stands for Modified base pair, and UP stands for unmodified base pair.

|  | **Base pair** | **H-bond length** | **X-ray/MP/UP** | | **H-bond length** | **X-ray/MP/UP** | | **H-bond length** | **X-ray/MP/UP** | | **RMSD (Å) Mod *vs.* X-ray**  **Mod** *vs.* **Unmod.** |
| --- | --- | --- | --- | --- | --- | --- | --- | --- | --- | --- | --- |
|  | **Adenine** |  |  |  |  |  |  |  |  |  |  |
| 1 | m^1^A:A tHW | N6(A)-N7(m^1^A) | 2.76/3.11/3.00 | | N6(m^1^A)-N1(A) | 2.78/2.75/3.00 | |  |  |  | 0.23/0.38 |
| 2 | m^1^A:U tHW(w) | N3(U)-N7(m^1^A) | 3.05/3.28/3.04 | | N6(m^1^A)-O(w) | 2.74/2.69/2.93 | | O(w)-O2(U) | 2.67/2.71/2.77 | | 0.26/0.22 |
| 3 | m^1^A:U tHW | N6(m^1^A)-O2(U) | 3.17/2.74/3.00 | | N3(U)-N7(m^1^A) | 2.88/2.91/2.84 | |  |  |  | 0.24/0.09 |
| 4 | m^1^A:m^5^U tWH | N3(m^5^U)-N7(mA) | 2.92/2.91/2.84 | | N6(m^1^A)-O2(m^5^U) | 3.25/2.72/3.00 | |  |  |  | 0.21/0.10 |
| 5 | m^6^_6_A:GtS(W)S(r) | O2’(G)-N1(m^6^_6_A) | 2.89/2.86/2.82 | | C2(m^6^_6_A)-N3(G) | 3.20/3.31/3.29 | | N2(G)-N3(m^6^_6_A) | 2.79/3.16/3.22 | | 0.45/0.16 |
|  | **Guanine** |  |  |  |  |  |  |  |  |  |  |
| 6 | m^1^G:C tHH1 | N4(C)-N7(m^1^G) | 2.83/3.12/3.14 | |  |  |  |  |  |  | 0.00/0.02 |
| 7 | m^2^G:U cWW | N3(U)-O6(m^2^G) | 3.17/2.84/2.84 | | N1(m^2^G)-O2(U) | 2.82/2.84/2.83 | | N2(m^2^G)-O2(U) | 3.18/3.56/3.59 | | 0.27/0.05 |
| 8 | m^2^G:C cWW | N4(C)-O6(m^2^G) | 2.84/2.79/2.79 | | N1(m^2^G)-N3(C) | 2.92/2.96/2.95 | | N2(m^2^G)-O2(C) | 2.93/2.93/2.92 | | 0.09/0.01 |
| 9 | m^2^G:C cWW1 | N1(m^2^G)-O2(C) | 3.19-/- | | N4(C)-O6(m^2^G) | -/2.80/2.79 | | N1(m^2^G)-N3(C) | -/3.40/2.95* | | 0.75/0.49 |
| 10 | m^2^_2_G:A cWW | N6(A)-O6(m^2^_2_G) | 2.75/2.84/2.85 | | N1(m^2^_2_G)-N1(A) | 3.24/3.04/2.97 | |  |  |  | 0.51/0.55 |
| 11 | m^7^G:C cWW | N4(C)-O6(m^7^G) | 3.08/2.94/2.79 | | N1(m^7^G)- N3(C) | 2.70/2.89/2.95 | | N2(m^7^G)O2(C) | 2.50/2.76/2.92 | | 0.21/0.08 |
| 12 | m^7^G:G tWH | N1(m^7^G)-N7(G) | 2.87/2.83/2.89 | | N2(m^7^G)-O6(G) | 3.00/2.83/3.15 | |  |  |  | 0.22/0.17 |
|  | **Cytosine** |  |  |  |  |  |  |  |  |  |  |
| 13 | m^5^C:G cWW | N4(m^5^C)-O6(G) | 3.36/2.79/2.79 | | N1(G)-N3(m^5^C) | 3.05/2.95/2.95 | | N2(G)-O2(m^5^C) | 2.69/2.91/2.92 | | 0.34/0.01 |
| 14 | m^5^C:G tWW | N1(G)-O2(m^5^C) | 2.85/2.84/2.85 | | N2(G)-O2(m^5^C) | -/3.00/3.01 | | N2(G)-N3(m^5^C) | 2.79-/- | | 1.28/0.01 |
|  | **Uracil** |  |  |  |  |  |  |  |  |  |  |
| 14 | m^5^U: m^1^A tWH | N3(m^5^U)-N7(m^1^A) | 2.92/2.91/2.84 | | N6(m^1^A)-O2(m^5^U) | 3.25/2.72/3.00 | |  |  |  | 0.21/0.10 |
| 15 | m^5^U:A tWH1 | N3(m^5^U)-N7(A) | 2.77/2.84/2.84 | | N6(A)-O2(m^5^U) | 3.18/2.99/3.00 | |  |  |  | 0.20/0.00 |
| 16 | m^5^U:G cWW | N3(m^5^U)-O6(G) | 2.56/2.85/2.84 | | N1(G)-O2(m^5^U) | 2.82/2.82/2.83 | |  |  |  | 0.28/0.01 |
| 17 | m^5^U:G tWH | N3(m^5^U)-N7(G) | 2.51/3.00/3.00 | |  |  |  |  |  |  | 0.54/0.01 |
| 18 | s^4^U:A tWH | N3(s^4^U)-N7(A) | 2.60/2.91/2.84 | | N6(A)-O2(s^4^U) | 2.79/2.94/3.00 | |  |  |  | 0.18/0.12 |
| 19 | H2U:U tWW | N3(H2U)-O2(U) | 2.66/2.90/2.89 | | N3(U)-O2(H2U) | 2.66/2.87/2.89 | |  |  |  | 0.30/0.20 |
| 20 | H2U:G cHS1 | N2(G)-O4(H2U) | 2.49/2.96/2.92 | |  |  |  |  |  |  | 1.08/1.27 |
| 21 | H2U:G tWS | N3(H2U)-N3(G) | 3.21/3.10/3.10 | | N2(G)-O2(H2U) | 2.63/2.87/2.89 | |  |  |  | 0.33/0.13 |
| 22 | Ψ:A cWW | N6(A)-O2(Ψ) | 2.90/2.95/2.92 | | N3(Ψ)-N1(A) | 2.85/2.88/2.88 | |  |  |  | 0.12/0.05 |
| 23 | Ψ:A cH:W | N6(A)-O2(Ψ) | 3.262.89/- | | N1(Ψ)-N1(A) | 2.71/2.91/- | | N1(A)-O4(U) | - /-/3.46 | | 0.30/0.36 |
| 24 | Ψ:U tWW | N3(U)-O2(Ψ) | 2.80/2.87/2.85 | | N3(Ψ)-O4(U) | 2.80/2.86/2.85 | |  |  |  | 0.12/0.06 |
| 25 | Ψ:G tBsW | N1(G)-O2(Ψ) | 2.78/2.94/2.92 | | N2(G)-O2(Ψ) | 2.94/3.09/3.17 | |  |  |  | 0.32/0.09 |
| 26 | Ψ:G cWW | N3(Ψ)-O6(G) | 2.83/2.83/2.84 | | N1(G)-O2(Ψ) | 2.91/2.81/2.83 | |  |  |  | 0.18/0.05 |
| 27 | Ψ:C cS(r)W | N4(U)-O4(Ψ) | 2.77/2.94/2.95 | | O2’(Ψ)-N3(C) | 3.03/2.79/2.77 | |  |  |  | 0.32/0.13 |
|  | **Non-natural** |  |  |  |  |  |  |  |  |  |  |
| 1’ | 5BrC:G cWW | N4(5BrC)-O6(G) | 2.97/2.79/2.79 | | N1(G)-N3(5BrC) | 2.88/2.96/2.95 | | N2(G)-O2(5BrC) | 2.78/2.92/2.92 | | 0.21/0.01 |
| 2’ | 5BrU:A cWW | N6(A)-O4(5BrU) | 2.95/2.95/2.92 | | N3(5BrU)-N1(A) | 2.79/2.86/2.88 | |  |  |  | 0.29/0.02 |
| 3’ | 5BrU:G cWW | N3(5BrU)-O6(G) | 2.73/2.81/2.84 | | N1 (G)-O2(5BrU) | 2.62/2.84/2.83 | |  |  |  | 0.22/0.07 |
| 4’ | 5BrU:5BrU cWW | N3(5BrU)-O4(5BrU) | 2.79/2.87/2.87 | | N3(5BrU)-O2(5BrU) | 3.36/2.86/2.87 | |  |  |  | 0.35/0.01 |
| 5’ | 5BrU:A cWH | N6(A)-O4(5BrU) | 2.76/2.97/3.00 | | N3 (5BrU)-N7(A) | 2.96/2.83/2.84 | |  |  |  | 0.44/0.02 |
| 6’ | 5BrU:A cS(r)H | N6(A)-O2(5BrU) | 2.86/3.00/2.98 | | O2’ (5BrU)-N7(A) | 2.54/2.75/2.75 | |  |  |  | 1.05/0.04 |
| 7’ | 5BrU:A cW(r)S | N6(A)-O2(5BrU) | 2.95/2.95/2.94 | | O2’(5BrU)-N1(A) | 2.73/2.75/2.75 | |  |  |  | 0.80/0.02 |
| 8’ | 5IU:A cWW | N6(A)-O4(5IU) | 2.94/2.94/2.92 | | N3(5IU)-N1(A) | 2.80/2.86/2.88 | |  |  |  | 0.20/0.02 |
| 9’ | 5IU:G cWW | N1(G)-O2(5IU) | 2.73/2.84/2.83 | | N3(5IU)-O6(G) | 2.75/2.81/2.84 | |  |  |  | 0.22/0.07 |

* plus N2(G)-O2(C) 2.92 Å

Coordinates of the B3LYP optimized geometries of modified base pairs and corresponding optimized unmodified pairs:

S1: m^1^A:A tHW

E = -1053.35875607 A.U.

C 6.587618 0.794842 0.493644

N 1.470751 0.530302 0.088331

C 2.289200 1.520715 0.501715

N 3.604818 1.517607 0.579216

C 4.124613 0.348356 0.182164

C 3.409373 -0.762240 -0.259981

C 2.010335 -0.646573 -0.295822

N 1.213453 -1.646228 -0.708997

N 4.253133 -1.801708 -0.573474

C 5.444808 -1.322030 -0.328041

N 5.440968 -0.024694 0.134111

C -3.146395 3.433314 -0.375398

C -5.129270 -2.756691 0.450389

N -3.349478 1.988492 -0.202009

C -4.638632 1.512440 -0.081150

N -4.972257 0.275733 0.086920

C -3.920616 -0.563842 0.125336

C -2.578950 -0.214973 0.005679

C -2.250945 1.148765 -0.152125

N -1.026274 1.621558 -0.246491

N -1.787197 -1.332931 0.066350

C -2.634153 -2.320852 0.227235

N -3.944874 -1.918648 0.271561

H 6.675359 1.643876 -0.182068

H 1.792465 2.434029 0.811889

H 1.642583 -2.533494 -0.907926

H 6.369926 -1.860417 -0.460093

H -2.644229 3.634791 -1.321038

H -4.113405 3.924345 -0.386641

H -2.562059 3.834930 0.451745

H -6.007615 -2.138147 0.293818

H -5.407745 2.269975 -0.135322

H -0.139532 1.034865 -0.123911

H -2.361821 -3.360400 0.314641

H 0.220296 -1.604748 -0.519381

H -5.151585 -3.166339 1.458210

H -5.121916 -3.565955 -0.275843

H 7.486435 0.187783 0.426480

H 6.477945 1.164742 1.510944

H -0.878306 2.606403 -0.386001

S1: Unmodified: AA tHW

E = -1013.64169996 A.U.

C -6.590306 -0.576258 -0.013118

N -1.462198 -0.816460 -0.013964

C -2.402665 -1.772033 -0.039782

N -3.722756 -1.638328 -0.041318

C -4.081149 -0.349770 -0.013398

C -3.228060 0.748454 0.014429

C -1.844475 0.476332 0.012909

N -0.921908 1.445648 0.037974

N -3.941420 1.928752 0.035926

C -5.190125 1.542966 0.021944

N -5.350458 0.175806 -0.010168

C 5.406841 2.385878 -0.038104

N 3.580770 -2.399757 0.024401

C 4.824724 -1.918590 0.017660

N 5.223244 -0.647910 0.002861

C 4.181545 0.184447 -0.003512

C 2.831318 -0.160322 0.003372

C 2.541382 -1.543297 0.017427

N 1.301058 -2.050010 0.025125

N 2.046896 0.979286 -0.003714

C 2.904367 1.969036 -0.015353

N 4.211999 1.561525 -0.014750

H -6.571745 -1.318690 -0.808607

H -2.028705 -2.789758 -0.062271

H -1.237368 2.398742 0.053318

H -6.044962 2.200878 0.030505

H 6.262768 1.727639 0.084075

H 5.612275 -2.663653 0.024679

H 0.451382 -1.486916 0.011407

H 2.642960 3.015388 -0.022628

H 0.077083 1.236713 0.025519

H 5.389413 3.108369 0.776811

H 5.497848 2.914273 -0.987115

H -7.418784 0.108300 -0.179259

H -6.734082 -1.090240 0.937084

H 1.224932 -3.051966 0.033026

S2: m^1^A:U tHW(water)

E = -1077.32794627 A.U.

C -5.437111 -1.727590 -0.586890

N -4.570645 -0.572524 -0.330670

C -3.234375 -0.822238 -0.087725

O -2.790952 -1.966268 -0.085521

N -2.458390 0.284724 0.142848

C -2.879969 1.625262 0.163674

O -2.078783 2.520031 0.377240

C -4.292317 1.789605 -0.088717

C -5.060303 0.709767 -0.323158

C 4.739571 -2.506013 -0.393987

C 2.552456 3.657055 -0.011087

N 4.031463 -1.225473 -0.263421

C 4.744996 -0.054999 -0.421365

N 4.259108 1.138462 -0.327846

C 2.939508 1.164455 -0.062697

C 2.105830 0.060922 0.110264

C 2.680170 -1.222017 0.019928

N 2.019547 -2.352606 0.185485

N 0.815902 0.465582 0.344930

C 0.880333 1.778006 0.320049

N 2.142531 2.258423 0.077910

O -0.490501 -2.911980 0.979805

H -5.079522 -2.279773 -1.452668

H -4.703417 2.785002 -0.088231

H -6.118824 0.794741 -0.520219

H 5.783192 -2.308458 -0.613284

H 4.680304 -3.068032 0.537446

H 4.317510 -3.090584 -1.210698

H 3.054013 3.835452 -0.959151

H 5.794876 -0.190658 -0.639491

H 2.478208 -3.241837 0.095295

H 0.023673 2.424045 0.452415

H 3.229726 3.902194 0.804212

H 1.664840 4.279111 0.054026

H -6.443845 -1.366852 -0.773324

H -5.440573 -2.394279 0.272456

H -1.467020 0.113179 0.300362

H -1.316552 -2.596429 0.552223

H 1.020104 -2.384541 0.473605

H -0.704158 -3.035939 1.908458

S2: Unmodified; A:U tHW (water)

E = -1037.60274455 A.U.

C 5.046190 1.412954 -0.726225

N 4.155401 0.298664 -0.407722

C 2.820594 0.602276 -0.169180

O 2.428474 1.760804 -0.229404

N 2.010111 -0.461008 0.125305

C 2.403748 -1.803839 0.234847

O 1.599528 -2.675660 0.513870

C 3.814279 -2.026348 -0.015264

C 4.612980 -0.990858 -0.323190

C -3.274922 -3.151421 0.073280

N -3.921001 1.893505 -0.466882

C -4.765628 0.868777 -0.586054

N -4.518244 -0.428308 -0.420026

C -3.235742 -0.640996 -0.122167

C -2.228200 0.315733 0.015427

C -2.633825 1.657911 -0.150535

N -1.813859 2.719938 -0.033728

N -1.020139 -0.304544 0.303333

C -1.310467 -1.583416 0.346857

N -2.628291 -1.854016 0.100944

O 0.673888 3.054041 1.477748

H 4.685610 1.944475 -1.604035

H 4.191129 -3.033235 0.047820

H 5.667546 -1.117439 -0.521420

H -3.778042 -3.298964 -0.880872

H -5.785535 1.131295 -0.844136

H -2.271837 3.615029 -0.070209

H -0.587702 -2.361412 0.536423

H -4.015358 -3.226848 0.868796

H -2.520200 -3.922181 0.208266

H 6.038420 1.016008 -0.920999

H 5.090235 2.114922 0.104433

H 1.007114 -0.248842 0.254135

H 1.313812 2.609127 0.892110

H -0.932691 2.673306 0.469447

H 0.940501 3.977043 1.487201

S3: m^1^A:U tHW

E = -1000.84939337 A.U.

C -4.525377 -2.299256 0.000205

N -4.008875 -0.928449 0.000076

C -2.642626 -0.752782 -0.000033

O -1.866287 -1.715101 -0.000005

N -2.212684 0.536313 -0.000191

C -2.992128 1.705673 -0.000109

O -2.448185 2.793624 -0.000154

C -4.415274 1.434969 0.000007

C -4.851129 0.162269 0.000098

C 3.404460 -3.248514 0.000012

C 3.414735 3.313402 0.000338

N 3.164380 -1.798423 -0.000012

C 4.250796 -0.944876 -0.000027

N 4.196411 0.346644 -0.000017

C 2.941162 0.832509 -0.000004

C 1.771211 0.080547 -0.000026

C 1.869445 -1.324248 -0.000040

N 0.834759 -2.143940 -0.000076

N 0.678264 0.902060 -0.000073

C 1.174621 2.116075 -0.000072

N 2.545821 2.138204 -0.000052

H -4.181791 -2.832357 -0.883536

H -5.103068 2.264038 0.000037

H -5.903649 -0.079717 0.000198

H 4.474267 -3.426715 0.000188

H 2.976289 -3.702204 -0.893142

H 2.976011 -3.702207 0.893029

H 4.046127 3.308204 -0.884990

H 5.212017 -1.439026 -0.000051

H 0.579385 3.016328 -0.000123

H -4.181654 -2.832243 0.883962

H -5.610192 -2.258395 0.000285

H 2.791365 4.202448 -0.003001

H 4.041283 3.311436 0.889140

H -1.196525 0.668252 -0.000215

H -0.148627 -1.806254 -0.000061

H 0.958612 -3.141236 -0.000136

S3-unmodified A:U tHW

E = -961.133821684 A.U.

C -4.654043 -1.954164 0.000659

N -3.941809 -0.679390 0.000714

C -2.550394 -0.725392 -0.000112

O -1.955441 -1.796157 -0.000350

N -1.922614 0.486687 -0.000458

C -2.519492 1.750930 -0.000370

O -1.836323 2.761270 -0.000514

C -3.970714 1.709139 0.000045

C -4.603867 0.522453 0.000553

C 3.802353 2.746461 0.000472

N 3.243198 -2.353424 0.000076

C 4.328551 -1.574974 0.000513

N 4.398611 -0.244659 0.000639

C 3.182055 0.302650 0.000211

C 1.964410 -0.372166 -0.000307

C 2.026081 -1.781119 -0.000357

N 0.939953 -2.570341 -0.000921

N 0.920611 0.531810 -0.000593

C 1.493351 1.709885 -0.000273

N 2.860637 1.642972 0.000167

H -4.392278 -2.535612 -0.881354

H -4.514855 2.638729 0.000109

H -5.682085 0.449146 0.000973

H 4.436725 2.702367 -0.883475

H 5.276769 -2.100461 0.000887

H 0.956555 2.645745 -0.000359

H -4.390825 -2.536337 0.881732

H -5.721958 -1.754041 0.001612

H 3.247950 3.681584 0.001071

H 4.437101 2.701439 0.884091

H -0.881842 0.459572 -0.000734

H -0.005686 -2.207325 -0.000768

H 1.088691 -3.563521 -0.000456

S4: m^1^A:m^5^U tHW

E = -1040.18357206 A.U.

C -3.980862 -2.847427 0.001785

N -3.601599 -1.432998 0.000788

C -2.265948 -1.124608 0.000198

O -1.390871 -2.001129 0.000492

N -1.972109 0.203066 -0.000725

C -2.867117 1.279340 -0.000946

O -2.440182 2.420641 -0.001682

C -4.271012 0.887059 -0.000236

C -5.313529 1.960485 -0.000432

C -4.555395 -0.430459 0.000562

C 3.988982 -3.005468 0.000041

C 3.360882 3.525009 0.005167

N 3.608784 -1.585718 -0.000336

C 4.606777 -0.630462 -0.001060

N 4.426660 0.649639 -0.001012

C 3.129831 1.010211 -0.000446

C 2.038512 0.148306 -0.000215

C 2.273146 -1.240782 -0.000208

N 1.324229 -2.157453 0.000049

N 0.870956 0.859955 -0.000833

C 1.247139 2.116286 -0.001307

N 2.609763 2.271427 -0.001656

H -3.586933 -3.345372 -0.881632

H -6.316876 1.538078 0.000177

H -5.205647 2.603012 -0.875252

H -5.204987 2.603959 0.873609

H -5.575558 -0.786389 0.001094

H 3.606674 -3.498997 -0.892766

H 5.071064 -3.078477 -0.000131

H 3.606993 -3.498424 0.893297

H 4.047174 3.548040 -0.837710

H 5.611606 -1.028641 -0.001718

H 0.567716 2.954740 -0.002328

H -3.586678 -3.344214 0.885745

H -5.064331 -2.913628 0.001984

H 3.924631 3.617449 0.930758

H 2.657830 4.348541 -0.076028

H -0.975887 0.441343 -0.001113

H 0.309557 -1.920000 0.000189

H 1.546847 -3.137483 0.000012

S4 Unmodified: A:m^5^U tHW

E = -1000.46683185 A.U.

C 4.148324 -2.574791 -0.000603

N 3.570228 -1.234051 -0.000698

C 2.187813 -1.137331 -0.000532

O 1.477785 -2.137280 -0.000696

N 1.694326 0.136618 -0.000254

C 2.422721 1.323841 0.000220

O 1.852607 2.404545 0.000551

C 3.873064 1.151208 0.000255

C 4.736606 2.373605 0.000639

C 4.359154 -0.105142 -0.000161

C -3.759128 2.992791 -0.002262

N -3.742267 -2.137583 0.000192

C -4.739003 -1.248672 0.000145

N -4.668345 0.081600 0.000032

C -3.400579 0.497254 0.000150

C -2.261126 -0.302650 0.000277

C -2.471249 -1.697408 0.000409

N -1.474597 -2.596473 0.000800

N -1.127615 0.485788 0.000677

C -1.572455 1.717790 0.000784

N -2.939156 1.796136 0.000895

H 3.829142 -3.126778 0.881620

H 4.531271 2.993699 -0.873358

H 5.793959 2.110577 0.001026

H 4.530599 2.993651 0.874502

H 5.422980 -0.298540 -0.000188

H -4.416864 3.000931 0.865346

H -5.737487 -1.670987 0.000061

H -0.495202 -2.335797 0.000137

H -0.939538 2.591564 0.001267

H 3.826472 -3.127762 -0.881184

H 5.231071 -2.485351 -0.002262

H -4.372433 3.030184 -0.901414

H -3.109637 3.864029 0.028300

H 0.657251 0.223586 -0.000285

H -1.727598 -3.568349 0.000160

S5: m^6^_6_A:G tS(W)S(r)

E = -1588.65840732 A.U.

C 3.298819 -2.880187 0.833245

O 3.287666 -1.610168 1.510606

C 2.572938 -2.621642 -0.495712

O 2.048465 -3.763668 -1.081242

C 1.535710 -1.542286 -0.087840

O 0.437309 -2.182591 0.530682

C 2.334242 -0.729506 0.952714

N 1.912328 4.106820 -0.464318

C 0.956143 3.256161 0.016450

N -0.295691 3.769770 0.207052

N 1.211105 2.008964 0.325633

C 2.506676 1.660736 0.132544

C 3.551838 2.435048 -0.357909

C 3.293364 3.801841 -0.707379

O 4.020220 4.671814 -1.138913

N 4.716771 1.703075 -0.400368

C 4.384998 0.526032 0.049975

N 3.044267 0.426720 0.386317

C -4.868811 3.446390 0.448864

N -2.050566 -0.809563 0.086326

C -1.716207 0.466562 0.299993

N -2.517394 1.523172 0.371398

C -3.800760 1.177888 0.213947

C -4.305742 -0.105234 -0.012219

C -3.350030 -1.156785 -0.088474

N -3.624950 -2.452809 -0.327748

N -5.684446 -0.052394 -0.121154

C -5.984081 1.207338 0.035903

C -4.977026 -2.991289 -0.410688

N -4.889637 2.010514 0.244174

C -2.538914 -3.424107 -0.391164

H 2.723176 -3.607017 1.410597

H 3.271245 -2.213673 -1.228249

H 1.220402 -0.918408 -0.924532

H -0.405565 -1.719171 0.336384

H 1.668705 -0.354172 1.729847

H -1.042615 3.097679 0.356321

H 5.053451 -0.304331 0.187388

H -4.295722 3.687844 1.342064

H -0.660625 0.668685 0.431125

H -6.981727 1.616205 0.014983

H -5.155421 -3.686350 0.414387

H -5.709811 -2.195291 -0.367021

H -5.098165 -3.537811 -1.348152

H -1.754564 -3.086390 -1.063277

H -2.090665 -3.597899 0.588692

H -2.946547 -4.360813 -0.763356

H -4.419279 3.952387 -0.405271

H -5.889768 3.798724 0.573286

H -0.554532 4.572326 -0.340693

H 1.679579 5.071231 -0.649916

C 4.750545 -3.362635 0.701765

H 5.321534 -3.029535 1.561793

H 4.782795 -4.446454 0.660525

H 5.207924 -2.966968 -0.199940

C 1.091931 -4.796888 -1.088160

H 0.817117 -5.102879 -0.080470

H 0.200420 -4.473019 -1.618486

H 1.543061 -5.632519 -1.613425

S5- unmodified: m^6^_6_A:G tS(W)S(r)

E = -1509.97419828 A.U.

C 3.611161 -2.525631 0.628687

O 3.347338 -1.318709 1.369503

C 2.791432 -2.393416 -0.669222

O 2.522766 -3.646230 -1.295727

C 1.563063 -1.551543 -0.178118

O 0.622211 -2.423803 0.438763

C 2.218702 -0.631809 0.874651

N 0.888929 4.123670 -0.299457

C 0.117185 3.107262 0.188850

N -1.192968 3.397875 0.452895

N 0.593884 1.912679 0.437973

C 1.918342 1.793508 0.168909

C 2.789174 2.752954 -0.336067

C 2.287231 4.067217 -0.614327

O 2.834075 5.061606 -1.041845

N 4.057063 2.235641 -0.469106

C 3.956179 1.003446 -0.059341

N 2.673705 0.661406 0.340224

C -5.819391 2.059711 0.266609

N -2.000526 -1.378741 0.283844

C -2.015524 -0.073923 0.587753

N -3.047027 0.762916 0.552988

C -4.175772 0.149071 0.174797

C -4.306669 -1.197060 -0.161272

C -3.140333 -1.979502 -0.101307

N -3.132406 -3.287742 -0.410325

N -5.607372 -1.497932 -0.501639

C -6.238054 -0.361981 -0.371102

N -5.432933 0.679836 0.037287

H 3.265706 -3.396060 1.189528

H 3.348880 -1.807045 -1.400723

H 1.095537 -0.975747 -0.977441

H -0.287896 -2.037047 0.389358

H 1.516242 -0.421524 1.680533

H -1.816209 2.609990 0.588169

H 4.763912 0.297490 0.007221

H -5.449300 2.392868 1.234207

H -1.062045 0.350284 0.871983

H -7.290414 -0.209807 -0.551998

H -5.414587 2.709527 -0.508998

H -6.904312 2.128204 0.256665

H -1.602507 4.173599 -0.038576

H 0.486715 5.038853 -0.438870

H -2.275423 -3.809578 -0.374332

H -3.977727 -3.733510 -0.717217

C 5.128943 -2.637455 0.420467

H 5.643622 -2.169617 1.252860

H 5.421052 -3.680975 0.366086

H 5.423172 -2.143840 -0.500603

C 1.414012 -4.350289 -0.728077

H 1.363667 -4.161436 0.316754

H 0.529208 -3.787378 -0.890147

H 1.411051 -5.400778 -1.044139

S6: m^1^G:C tHH

E = -1055.82002617 A.U.

C -2.799355 -3.233563 0.034040

C -4.393185 3.099752 0.013800

N -2.947150 -1.779659 -0.003615

C -4.198676 -1.212727 -0.014235

N -5.274996 -2.057708 0.033345

N -4.435131 0.077180 -0.018155

C -3.316217 0.824897 0.001376

C -2.000004 0.392633 0.001836

C -1.723184 -1.007644 -0.005772

O -0.646123 -1.574066 -0.008793

N -1.139443 1.470706 0.010814

C -1.921194 2.515492 0.016289

N -3.260794 2.192352 0.010501

C 6.136774 -1.750658 0.013758

N 4.993427 -0.850800 0.006016

C 5.269676 0.553406 -0.007227

O 6.440066 0.901473 -0.011591

N 4.203665 1.398089 -0.013667

C 2.968161 0.931621 -0.009339

N 1.962660 1.830154 -0.017528

C 2.660274 -0.468048 0.002457

C 3.718947 -1.309260 0.009913

H -3.341194 -3.649894 0.882400

H -1.740490 -3.443853 0.135378

H -3.156745 -3.689450 -0.891783

H -5.008087 2.938111 0.897814

H -6.154684 -1.597317 -0.130611

H -1.595053 3.542994 0.024545

H 6.753053 -1.587307 -0.869090

H 2.220525 2.800894 -0.020521

H 1.645292 -0.833770 0.005043

H 3.597777 -2.384195 0.019280

H 0.984990 1.568414 -0.006267

H -4.022751 4.121729 0.017648

H -5.007317 2.944569 -0.871904

H 5.779087 -2.778350 0.028769

H 6.758938 -1.563557 0.887606

H -5.192652 -2.985874 -0.340592

S6 unmodified: G:C tHH

E = -1016.49920188 A.U.

C -4.689261 2.704463 0.007191

N -2.983591 -2.044482 0.000360

C -4.269907 -1.579905 -0.011758

N -5.268428 -2.515337 0.036146

N -4.574050 -0.308923 -0.020436

C -3.486013 0.494530 -0.001014

C -2.142879 0.139155 0.004025

C -1.795166 -1.250469 0.007822

O -0.711760 -1.800095 0.021867

N -1.348495 1.264032 0.013720

C -2.189232 2.262583 0.013976

N -3.507035 1.861955 0.004640

C 6.048657 -1.689004 0.008741

N 4.874575 -0.829533 0.005161

C 5.101422 0.583231 -0.015934

O 6.258639 0.972457 -0.030108

N 4.006171 1.390036 -0.018533

C 2.788153 0.880350 -0.002928

N 1.751699 1.743838 -0.006780

C 2.529449 -0.528875 0.017241

C 3.617084 -1.332421 0.020492

H -5.278804 2.531753 0.906240

H -6.187243 -2.142851 -0.138114

H -1.923196 3.307170 0.019885

H 6.654577 -1.505377 -0.877237

H 1.975163 2.722960 -0.019649

H 1.528579 -0.931412 0.028935

H 3.533396 -2.410834 0.035327

H 0.784337 1.448284 0.005765

H -4.376669 3.745115 -0.021815

H -5.307827 2.491174 -0.862823

H 5.727019 -2.728456 0.026681

H 6.668115 -1.479294 0.879403

H -5.095105 -3.423078 -0.361381

H -2.807137 -3.036975 0.057652

S7: m^2^G:U cWW

E = -1075.72045253 A.U.

C -2.283295 3.792694 -0.054800

C -5.808659 -0.132327 -0.011342

N -0.796333 0.439946 0.036515

C -1.781176 1.389173 0.035144

N -1.362512 2.681043 0.078496

N -3.063100 1.104985 0.014069

C -3.297231 -0.225987 0.005272

C -2.381967 -1.276285 0.005262

C -0.988712 -0.955090 0.018579

O -0.011496 -1.699977 0.016046

N -3.034914 -2.489486 -0.014253

C -4.298999 -2.179082 -0.024839

N -4.530816 -0.815110 -0.015636

C 4.352229 2.400530 -0.010429

N 4.160274 0.953236 -0.007527

C 2.856891 0.479920 -0.000646

O 1.913097 1.273439 0.003595

N 2.708974 -0.874193 0.001711

C 3.743364 -1.833685 -0.003566

O 3.494183 -3.018941 -0.000927

C 5.075554 -1.249310 -0.012335

C 5.226153 0.083702 -0.013922

H -3.076207 3.714979 0.686570

H -1.733306 4.715937 0.114127

H -2.748742 3.830515 -1.043080

H -5.963611 0.395946 0.929353

H -5.118372 -2.880193 -0.041169

H 3.912399 2.843259 -0.902418

H 5.923360 -1.913821 -0.016831

H 6.198720 0.554216 -0.019984

H 0.184038 0.737592 0.049916

H 1.735469 -1.230290 0.007442

H -5.854216 0.589557 -0.825217

H -6.599242 -0.867657 -0.141185

H 5.418678 2.606989 0.007636

H 3.880914 2.848490 0.862170

H -0.382061 2.849856 -0.064936

S7 unmodified G:U cWW

E = -1036.40069105 A.U.

C -5.923056 0.385992 0.032088

N -0.888292 0.701425 -0.043305

C -1.822568 1.693340 -0.036275

N -1.347447 2.973432 -0.105190

N -3.115296 1.478852 -0.007763

C -3.419958 0.162341 0.000921

C -2.559287 -0.933344 -0.003794

C -1.151983 -0.685101 -0.022719

O -0.214503 -1.478092 -0.022037

N -3.273836 -2.111311 0.016598

C -4.520121 -1.735969 0.032161

N -4.681847 -0.362051 0.024929

C 4.355289 2.381162 0.039763

N 4.088971 0.945611 0.021966

C 2.763289 0.539389 0.014879

O 1.860569 1.379141 0.023528

N 2.547447 -0.805502 -0.002694

C 3.531279 -1.816580 -0.011912

O 3.221487 -2.987421 -0.027197

C 4.891227 -1.300833 -0.000868

C 5.109128 0.022870 0.015020

H -5.962088 1.052665 0.892489

H -5.374446 -2.393998 0.049729

H 3.897575 2.863191 -0.821742

H 5.704379 -2.007303 -0.006830

H 6.104335 0.443406 0.022843

H 0.106584 0.949743 -0.058382

H 1.557629 -1.110929 -0.009532

H -6.017509 0.983681 -0.873919

H -6.753067 -0.314488 0.085435

H 5.430715 2.532011 0.011100

H 3.949379 2.835095 0.942214

H -0.394711 3.145188 0.168259

H -2.016535 3.689914 0.116606

S8: m^2^G:C cWW

E = -1055.84619963 A.U.

C -0.848882 3.515544 -0.000268

C -5.538090 1.151241 0.002847

N -0.661765 -0.148736 -0.000310

C -1.229773 1.103096 -0.000247

N -0.380030 2.146489 -0.000094

N -2.535636 1.299608 -0.000059

C -3.234942 0.148982 -0.000197

C -2.766371 -1.163602 -0.000274

C -1.354052 -1.368745 -0.000506

O -0.726719 -2.431959 -0.000774

N -3.817978 -2.056971 -0.000739

C -4.881525 -1.306811 -0.000793

N -4.599831 0.048327 -0.001003

C 5.211484 1.774458 -0.000191

N 4.424160 0.545996 0.000104

C 3.015426 0.666969 -0.000222

O 2.530985 1.799688 -0.000822

N 2.276803 -0.465127 0.000167

C 2.843659 -1.670850 0.000598

N 2.045100 -2.738571 0.001035

C 4.270198 -1.815084 0.000692

C 5.004000 -0.679944 0.000459

H 0.021351 4.167801 0.001437

H -1.454100 3.732737 -0.882388

H -1.456815 3.731701 0.880184

H -5.423169 1.752012 0.904732

H -5.900561 -1.660365 -0.001487

H 4.978674 2.370021 -0.880723

H 1.017517 -2.630011 0.000402

H 4.744679 -2.783416 0.000938

H 6.084806 -0.693849 0.000544

H 6.267318 1.514869 0.000140

H 4.978298 2.370662 0.879807

H -5.375717 1.793858 -0.861668

H -6.549132 0.751974 -0.033587

H 0.623922 1.977972 -0.001385

H 0.363772 -0.226670 -0.000214

H 2.442468 -3.659753 0.000934

S8 unmodified: G:C cWW

E = -1016.52629096 A.U.

C 5.198949 -1.871772 0.010027

N 4.388179 -0.658545 0.005011

C 2.982064 -0.807265 -0.001143

O 2.520790 -1.949466 -0.002317

N 2.221281 0.310626 -0.005236

C 2.765148 1.526956 -0.001595

N 1.946908 2.579968 -0.006521

C 4.188393 1.698895 0.006637

C 4.944158 0.578217 0.009638

C -5.550682 -1.464573 0.019213

N -0.707387 -0.050759 -0.008229

C -1.245444 -1.310426 -0.009813

N -0.374358 -2.337560 -0.018070

N -2.544702 -1.540342 -0.004777

C -3.272107 -0.407843 -0.000925

C -2.834463 0.916233 -0.000045

C -1.428808 1.155367 -0.004006

O -0.823429 2.230723 -0.003929

N -3.907799 1.784052 0.003293

C -4.952661 1.008714 0.005113

N -4.638619 -0.339765 0.000343

H 4.979733 -2.467245 0.894157

H 2.327520 3.508175 -0.000055

H 4.643706 2.676356 0.010399

H 6.024470 0.613166 0.015596

H -5.286753 -2.173622 -0.763534

H 0.634706 -2.202067 -0.011331

H -0.760578 -3.262607 -0.009749

H -5.979920 1.337606 0.006180

H -5.514949 -1.981136 0.978793

H -6.561519 -1.101425 -0.152336

H 6.249583 -1.591988 0.005578

H 4.974754 -2.476576 -0.866284

H 0.921731 2.454372 -0.007166

H 0.316823 0.048195 -0.009591

S9 m^2^G:C cWW (1)

E = -1055.83094442 A.U.

C 0.313849 -2.676538 -1.196810

C -5.836895 -0.952284 0.536437

N -0.910382 -0.201380 -0.346490

C -1.627987 -1.370101 -0.321542

N -0.979631 -2.538093 -0.543538

N -2.925726 -1.417746 -0.088557

C -3.471878 -0.208495 0.125131

C -2.857490 1.039687 0.089087

C -1.458986 1.096394 -0.176565

O -0.736999 2.085010 -0.265726

N -3.774522 2.041850 0.332621

C -4.899945 1.414053 0.511902

N -4.789194 0.038523 0.398351

C 5.495166 -1.402218 0.937297

N 4.611010 -0.337839 0.478790

C 3.232423 -0.654687 0.322511

O 2.882055 -1.801065 0.577587

N 2.392761 0.327051 -0.099892

C 2.848701 1.551620 -0.346496

N 1.972905 2.486065 -0.737866

C 4.237622 1.884653 -0.216467

C 5.068841 0.905071 0.201914

H 1.118763 -2.262074 -0.591918

H 0.321532 -2.212956 -2.188522

H 0.512813 -3.739089 -1.314828

H -5.931950 -1.538454 -0.376903

H -5.849884 1.876258 0.729451

H 5.168117 -1.770967 1.907578

H 2.279368 3.437626 -0.824680

H 4.606686 2.875388 -0.429043

H 6.129159 1.066489 0.338908

H 0.967459 2.304504 -0.643551

H 0.107737 -0.223393 -0.410857

H -1.616000 -3.315390 -0.589719

H -5.618994 -1.630096 1.361149

H -6.777409 -0.442835 0.732848

H 6.507182 -1.010989 1.013181

H 5.473879 -2.237520 0.239692

S9: unmodified: G:C cWW

E = -1016.52629096 A.U.

C 5.198949 -1.871772 0.010027

N 4.388179 -0.658545 0.005011

C 2.982064 -0.807265 -0.001143

O 2.520790 -1.949466 -0.002317

N 2.221281 0.310626 -0.005236

C 2.765148 1.526956 -0.001595

N 1.946908 2.579968 -0.006521

C 4.188393 1.698895 0.006637

C 4.944158 0.578217 0.009638

C -5.550682 -1.464573 0.019213

N -0.707387 -0.050759 -0.008229

C -1.245444 -1.310426 -0.009813

N -0.374358 -2.337560 -0.018070

N -2.544702 -1.540342 -0.004777

C -3.272107 -0.407843 -0.000925

C -2.834463 0.916233 -0.000045

C -1.428808 1.155367 -0.004006

O -0.823429 2.230723 -0.003929

N -3.907799 1.784052 0.003293

C -4.952661 1.008714 0.005113

N -4.638619 -0.339765 0.000343

H 4.979733 -2.467245 0.894157

H 2.327520 3.508175 -0.000055

H 4.643706 2.676356 0.010399

H 6.024470 0.613166 0.015596

H -5.286753 -2.173622 -0.763534

H 0.634706 -2.202067 -0.011331

H -0.760578 -3.262607 -0.009749

H -5.979920 1.337606 0.006180

H -5.514949 -1.981136 0.978793

H -6.561519 -1.101425 -0.152336

H 6.249583 -1.591988 0.005578

H 4.974754 -2.476576 -0.866284

H 0.921731 2.454372 -0.007166

H 0.316823 0.048195 -0.009591

S10: m^2^_2_G:A cWW

E = -1167.54817944 A.U.

C -0.524725 2.542689 1.387816

C -2.368906 3.529238 0.127214

C -6.303563 0.374680 -0.714867

N -1.369287 0.008458 0.318944

C -2.157630 1.126226 0.213456

N -1.545302 2.350376 0.360014

N -3.444335 1.071290 -0.045515

C -3.907847 -0.187244 -0.193104

C -3.220382 -1.386319 -0.052164

C -1.820595 -1.329174 0.232570

O -1.028864 -2.254259 0.380960

N -4.064859 -2.457618 -0.243823

C -5.223376 -1.916421 -0.493175

N -5.200570 -0.534391 -0.476475

C 6.614012 1.059579 -0.897471

N 1.652797 0.102963 0.013049

C 2.280681 1.178329 -0.487146

N 3.576972 1.359035 -0.699202

C 4.283833 0.279775 -0.341271

C 3.779860 -0.907266 0.179947

C 2.381521 -0.986395 0.347814

N 1.768039 -2.073571 0.827182

N 4.793064 -1.811943 0.417436

C 5.875120 -1.178429 0.050344

N 5.642032 0.094258 -0.422246

H 0.111986 1.671713 1.490014

H 0.105628 3.386329 1.109796

H -0.980554 2.761128 2.361541

H -2.961021 3.397704 -0.772386

H -1.708360 4.386452 0.003415

H -3.053755 3.727826 0.959077

H -6.382224 1.092222 0.100121

H -0.345235 0.103998 0.314402

H -6.136836 -2.453222 -0.694840

H 6.319568 1.434129 -1.876089

H 1.634093 2.006853 -0.753426

H 2.325986 -2.895142 0.980872

H 6.875914 -1.578667 0.093617

H 6.688733 1.903100 -0.211589

H 7.584145 0.574507 -0.975271

H -6.162780 0.922093 -1.646860

H -7.224880 -0.199781 -0.775849

H 0.752621 -2.164780 0.740456

S10 unmodified: G:A cWW

E = -1088.91513101 A.U.

C 6.419637 1.126765 0.104814

N 1.444085 0.249570 0.021482

C 2.123310 1.420241 0.181165

N 1.360692 2.555620 0.291313

N 3.429429 1.517350 0.196671

C 4.038939 0.323018 0.028561

C 3.460001 -0.934101 -0.118881

C 2.032910 -1.027298 -0.116446

O 1.318708 -2.020665 -0.213938

N 4.431326 -1.903749 -0.241897

C 5.554948 -1.249224 -0.172671

N 5.389184 0.113595 -0.005958

C -6.570027 1.230412 -0.190031

N -1.518019 0.320835 -0.150699

C -2.224993 1.443052 -0.356671

N -3.539269 1.609462 -0.348315

C -4.173995 0.456919 -0.098137

C -3.585533 -0.784033 0.121804

C -2.176228 -0.838902 0.081753

N -1.491440 -1.970243 0.267683

N -4.541896 -1.753605 0.334023

C -5.673475 -1.106293 0.245048

N -5.526759 0.238360 -0.014422

H 6.363898 1.826594 -0.728399

H 0.413334 0.277004 -0.044620

H 6.540114 -1.683871 -0.233508

H -6.413029 2.065425 0.490532

H -1.635388 2.329824 -0.565427

H -2.011344 -2.821559 0.387693

H -6.652300 -1.544965 0.359236

H -6.570762 1.610601 -1.210975

H -7.532257 0.770586 0.022095

H 6.306903 1.684108 1.033632

H 7.391595 0.639290 0.096624

H -0.481109 -2.012833 0.108042

H 0.441396 2.456474 0.688991

H 1.874994 3.364954 0.596560

S11: m^7^G:C cWW

E = -1056.26003335 A.U.

C 5.417103 -1.778216 0.000720

N 4.582955 -0.574793 0.000315

C 3.189783 -0.742122 0.000435

O 2.740001 -1.892360 0.000965

N 2.401254 0.361067 -0.000033

C 2.932192 1.585370 -0.000531

N 2.100095 2.632455 -0.001018

C 4.346079 1.778774 -0.000622

C 5.120332 0.669281 -0.000199

C -3.890138 2.738667 0.000803

C -5.168148 -2.060293 -0.000273

N -0.428670 -0.226577 -0.000053

C -0.872927 -1.531123 -0.000429

N 0.046463 -2.484927 -0.000812

N -2.169910 -1.856774 -0.000484

C -2.972752 -0.804267 -0.000195

C -2.622644 0.534568 0.000143

C -1.239646 0.904976 0.000210

O -0.783675 2.044016 0.000447

N -3.792241 1.276857 0.000374

C -4.809908 0.426010 0.000190

N -4.360250 -0.842402 -0.000208

H 5.204762 -2.378460 0.882325

H 2.463785 3.567527 -0.001050

H 4.785928 2.762939 -0.001004

H 6.199400 0.721130 -0.000237

H -3.397597 3.133335 -0.883479

H -3.397192 3.132825 0.885088

H -4.939892 3.017313 0.001125

H -4.936878 -2.648292 -0.885029

H 1.058698 -2.280832 -0.000239

H -0.279370 -3.435799 -0.000834

H -5.847527 0.708590 0.000228

H -4.939498 -2.646830 0.886147

H -6.219353 -1.787790 -0.002062

H 6.461195 -1.478456 0.001230

H 5.205650 -2.378499 -0.881086

H 1.093181 2.491834 -0.000500

H 0.600263 -0.037889 0.000008

S11 unmodified: G:C cWW

E = -1016.52629096 A.U.

C 5.198949 -1.871772 0.010027

N 4.388179 -0.658545 0.005011

C 2.982064 -0.807265 -0.001143

O 2.520790 -1.949466 -0.002317

N 2.221281 0.310626 -0.005236

C 2.765148 1.526956 -0.001595

N 1.946908 2.579968 -0.006521

C 4.188393 1.698895 0.006637

C 4.944158 0.578217 0.009638

C -5.550682 -1.464573 0.019213

N -0.707387 -0.050759 -0.008229

C -1.245444 -1.310426 -0.009813

N -0.374358 -2.337560 -0.018070

N -2.544702 -1.540342 -0.004777

C -3.272107 -0.407843 -0.000925

C -2.834463 0.916233 -0.000045

C -1.428808 1.155367 -0.004006

O -0.823429 2.230723 -0.003929

N -3.907799 1.784052 0.003293

C -4.952661 1.008714 0.005113

N -4.638619 -0.339765 0.000343

H 4.979733 -2.467245 0.894157

H 2.327520 3.508175 -0.000055

H 4.643706 2.676356 0.010399

H 6.024470 0.613166 0.015596

H -5.286753 -2.173622 -0.763534

H 0.634706 -2.202067 -0.011331

H -0.760578 -3.262607 -0.009749

H -5.979920 1.337606 0.006180

H -5.514949 -1.981136 0.978793

H -6.561519 -1.101425 -0.152336

H 6.249583 -1.591988 0.005578

H 4.974754 -2.476576 -0.866284

H 0.921731 2.454372 -0.007166

H 0.316823 0.048195 -0.009591

S12: m^7^G:G tWH

E = -1203.92792362 A.U.

C -3.963827 3.304506 0.007770

N -4.317995 -1.747425 0.001079

C -5.316627 -0.801836 0.006556

N -6.594571 -1.254201 0.061964

N -5.082710 0.487594 0.000021

C -3.771279 0.791693 0.000031

C -2.692165 -0.081003 -0.009916

C -2.932748 -1.480768 -0.007936

O -2.130514 -2.411102 -0.008586

N -1.508993 0.621954 -0.013307

C -1.864085 1.881169 -0.006440

N -3.227542 2.048703 0.001951

C 4.733762 2.737532 0.001336

C 5.912638 -2.086093 0.011623

N 1.213736 -0.155896 -0.007920

C 1.630041 -1.462968 -0.004724

N 0.701262 -2.414847 -0.006851

N 2.919066 -1.819323 0.000166

C 3.745541 -0.782922 0.001712

C 3.424950 0.561750 -0.001091

C 2.047773 0.969589 -0.006097

O 1.612613 2.108508 -0.008512

N 4.609214 1.278006 0.002467

C 5.610068 0.406883 0.007185

N 5.132014 -0.851574 0.006869

H -4.569117 3.390979 -0.892502

H -6.820744 -2.190369 -0.223742

H -1.179037 2.713277 -0.006341

H 4.249745 3.139820 -0.884191

H 5.788365 2.997544 0.004443

H 4.244053 3.141674 0.882880

H 5.670677 -2.671694 -0.871934

H 1.029410 -3.364881 -0.003323

H 6.653309 0.667065 0.010538

H 0.191331 0.066271 -0.011418

H -0.309457 -2.251402 -0.008348

H 6.969720 -1.837084 0.011356

H 5.669762 -2.665861 0.898807

H -3.253038 4.125314 0.044637

H -4.615959 3.351943 0.877331

H -7.311299 -0.558940 -0.059031

H -4.553147 -2.729042 0.042490

S12 unmodified: G:G tWH

E = -1164.18491476 A.U.

C -3.705796 3.264095 0.007107

N -4.146197 -1.781568 0.071743

C -5.125039 -0.821466 0.109036

N -6.415003 -1.263715 0.243637

N -4.880734 0.461483 0.071521

C -3.560279 0.748491 0.004762

C -2.496803 -0.140142 -0.052306

C -2.746842 -1.542018 -0.009255

O -1.965514 -2.480973 -0.022224

N -1.308665 0.541145 -0.122778

C -1.634809 1.805032 -0.107808

N -2.997943 1.996366 -0.032202

C 6.336338 -1.477706 0.146651

N 1.524767 -0.008896 -0.142509

C 2.048182 -1.267307 -0.139643

N 1.174363 -2.299738 -0.261846

N 3.339034 -1.515656 -0.056106

C 4.076799 -0.387819 0.016031

C 3.656731 0.940237 0.018104

C 2.250213 1.206193 -0.063981

O 1.646466 2.270337 -0.071653

N 4.738697 1.791319 0.109365

C 5.772901 1.001939 0.160465

N 5.442107 -0.340068 0.107552

H -4.389465 3.343290 -0.836522

H -6.656250 -2.157953 -0.149784

H -0.922885 2.615492 -0.143052

H 6.191390 -2.106980 -0.730651

H 1.557791 -3.215775 -0.116676

H 6.802028 1.316388 0.234812

H 0.505691 0.109699 -0.183065

H 0.177396 -2.184161 -0.137251

H 7.362529 -1.117261 0.162190

H 6.156455 -2.081397 1.036186

H -2.977664 4.069048 -0.043693

H -4.275919 3.352660 0.930461

H -7.106424 -0.544092 0.109912

H -4.394863 -2.755748 0.164549

S13: m^5^C:G cWW

E = -1055.89068696 A.U.

C 5.921849 -1.190581 0.003368

N 0.969779 -0.110123 -0.001998

C 1.590886 -1.336035 0.002491

N 0.794575 -2.413654 0.008108

N 2.908219 -1.476949 0.004339

C 3.563387 -0.303212 0.002060

C 3.031811 0.984609 0.000241

C 1.617290 1.128728 -0.004624

O 0.954141 2.177678 -0.011519

N 4.044166 1.927728 -0.002618

C 5.144866 1.222823 -0.001671

N 4.921476 -0.137153 0.000715

C -4.857824 2.515733 0.007853

C -4.749219 -2.441421 -0.004277

N -4.045266 -1.160132 -0.003480

C -2.649595 -1.169659 -0.002913

O -2.063039 -2.265493 -0.004306

N -1.997297 0.013880 -0.002103

C -2.665063 1.172376 0.001261

N -1.942722 2.296336 0.004883

C -4.101283 1.220854 0.002916

C -4.725434 0.018007 0.000726

H 5.824361 -1.805470 0.897360

H -0.224413 -2.340135 0.000237

H 6.151692 1.621160 -0.003534

H -5.931261 2.332812 0.008954

H -4.621813 3.117530 0.889866

H -4.624396 3.122809 -0.871128

H -4.481201 -3.017097 -0.888274

H -2.378413 3.205359 0.004179

H -5.807972 -0.061695 0.001551

H -0.922347 2.248275 -0.001015

H -0.063232 -0.081725 -0.003022

H 1.232819 -3.325208 0.002719

H -4.479925 -3.019718 0.877997

H -5.818747 -2.249432 -0.003113

H 5.807387 -1.824532 -0.874913

H 6.908105 -0.733281 -0.010775

S13 unmodified: C:G cWW

E = -1016.52629096 A.U.

C 5.198949 -1.871772 0.010027

N 4.388179 -0.658545 0.005011

C 2.982064 -0.807265 -0.001143

O 2.520790 -1.949466 -0.002317

N 2.221281 0.310626 -0.005236

C 2.765148 1.526956 -0.001595

N 1.946908 2.579968 -0.006521

C 4.188393 1.698895 0.006637

C 4.944158 0.578217 0.009638

C -5.550682 -1.464573 0.019213

N -0.707387 -0.050759 -0.008229

C -1.245444 -1.310426 -0.009813

N -0.374358 -2.337560 -0.018070

N -2.544702 -1.540342 -0.004777

C -3.272107 -0.407843 -0.000925

C -2.834463 0.916233 -0.000045

C -1.428808 1.155367 -0.004006

O -0.823429 2.230723 -0.003929

N -3.907799 1.784052 0.003293

C -4.952661 1.008714 0.005113

N -4.638619 -0.339765 0.000343

H 4.979733 -2.467245 0.894157

H 2.327520 3.508175 -0.000055

H 4.643706 2.676356 0.010399

H 6.024470 0.613166 0.015596

H -5.286753 -2.173622 -0.763534

H 0.634706 -2.202067 -0.011331

H -0.760578 -3.262607 -0.009749

H -5.979920 1.337606 0.006180

H -5.514949 -1.981136 0.978793

H -6.561519 -1.101425 -0.152336

H 6.249583 -1.591988 0.005578

H 4.974754 -2.476576 -0.866284

H 0.921731 2.454372 -0.007166

H 0.316823 0.048195 -0.009591

S14: m^5^C:G tWW

E = -1055.88577528 A.U.

C 5.608957 -2.041788 0.353995

N 1.269531 0.567051 0.078116

C 1.479468 -0.732943 0.460055

N 0.392212 -1.429798 0.856600

N 2.673960 -1.298232 0.484608

C 3.658720 -0.464494 0.098137

C 3.554868 0.858954 -0.316480

C 2.264129 1.466672 -0.342045

O 1.962014 2.616178 -0.667869

N 4.800850 1.364472 -0.640155

C 5.622335 0.369964 -0.424258

N 4.991193 -0.768756 0.025131

C -6.031010 -1.503773 -0.478971

C -3.781397 2.779930 0.600342

N -3.702701 1.355716 0.278087

C -2.442565 0.759175 0.199492

O -1.436722 1.460591 0.400211

N -2.371263 -0.559626 -0.089750

C -3.473974 -1.274538 -0.311266

N -3.319716 -2.574617 -0.601384

C -4.787254 -0.700664 -0.238708

C -4.825930 0.620940 0.062248

H 5.420550 -2.297884 1.395758

H -0.533341 -1.141448 0.540108

H 6.695009 0.391777 -0.569764

H -6.916384 -0.878556 -0.375106

H -6.121509 -2.329784 0.231470

H -6.044940 -1.935080 -1.483591

H -3.331504 2.969982 1.573093

H -2.390150 -2.956645 -0.722633

H -5.765485 1.158195 0.142815

H 5.211484 -2.832632 -0.280879

H 6.681058 -1.959107 0.193853

H -4.826986 3.074674 0.616521

H -3.246888 3.363372 -0.146802

H 0.311690 0.938752 0.141403

H 0.536174 -2.422854 0.997998

H -4.097654 -3.174983 -0.826991

S14 unmodified: C:G tWW

E = -1016.51037011 A.U.

C 5.650049 1.451169 0.511132

N 0.953379 -0.019885 -0.565673

C 1.515788 1.221385 -0.652344

N 0.712451 2.207696 -1.130184

N 2.764444 1.474787 -0.330426

C 3.421276 0.371315 0.089501

C 2.951167 -0.932772 0.228962

C 1.583652 -1.202155 -0.106905

O 0.942755 -2.241734 -0.046841

N 3.948231 -1.759827 0.701750

C 4.982166 -0.980508 0.839146

N 4.730734 0.332641 0.485037

H -6.006845 -0.807899 1.397869

C -2.334111 -2.116766 -1.007474

N -3.195011 -1.103799 -0.386423

C -2.822634 0.256555 -0.498380

O -1.785913 0.526849 -1.110588

N -3.619277 1.191643 0.067647

C -4.712376 0.838980 0.718146

N -5.467166 1.832844 1.240842

C -5.114667 -0.520277 0.864996

C -4.312931 -1.449015 0.287877

H 5.803837 1.848125 -0.492034

H -0.286024 2.051472 -1.151578

H 5.956253 -1.283039 1.189844

H -2.183433 -1.869227 -2.055747

H -5.111353 2.771181 1.189717

H -4.528609 -2.506572 0.339600

H 5.261812 2.250187 1.141641

H 6.603241 1.112279 0.910559

H -2.827112 -3.082411 -0.927932

H -1.360918 -2.163049 -0.518737

H -0.029678 -0.103908 -0.817568

H 1.053730 3.145380 -1.022184

H -6.238408 1.636409 1.850039

S15: m^5^U:A tWH

E = -1000.46683185 A.U.

C 4.148324 -2.574791 -0.000603

N 3.570228 -1.234051 -0.000698

C 2.187813 -1.137331 -0.000532

O 1.477785 -2.137280 -0.000696

N 1.694326 0.136618 -0.000254

C 2.422721 1.323841 0.000220

O 1.852607 2.404545 0.000551

C 3.873064 1.151208 0.000255

C 4.736606 2.373605 0.000639

C 4.359154 -0.105142 -0.000161

C -3.759128 2.992791 -0.002262

N -3.742267 -2.137583 0.000192

C -4.739003 -1.248672 0.000145

N -4.668345 0.081600 0.000032

C -3.400579 0.497254 0.000150

C -2.261126 -0.302650 0.000277

C -2.471249 -1.697408 0.000409

N -1.474597 -2.596473 0.000800

N -1.127615 0.485788 0.000677

C -1.572455 1.717790 0.000784

N -2.939156 1.796136 0.000895

H 3.829142 -3.126778 0.881620

H 4.531271 2.993699 -0.873358

H 5.793959 2.110577 0.001026

H 4.530599 2.993651 0.874502

H 5.422980 -0.298540 -0.000188

H -4.416864 3.000931 0.865346

H -5.737487 -1.670987 0.000061

H -0.495202 -2.335797 0.000137

H -0.939538 2.591564 0.001267

H 3.826472 -3.127762 -0.881184

H 5.231071 -2.485351 -0.002262

H -4.372433 3.030184 -0.901414

H -3.109637 3.864029 0.028300

H 0.657251 0.223586 -0.000285

H -1.727598 -3.568349 0.000160

S15 unmodified: U:A tWH

E = -961.133821628 A.U.

C -4.654319 -1.954113 0.001729

N -3.942036 -0.679376 0.000744

C -2.550539 -0.725473 -0.000826

O -1.955696 -1.796285 -0.001544

N -1.922718 0.486554 -0.001517

C -2.519531 1.750859 -0.000343

O -1.836256 2.761144 -0.000836

C -3.970702 1.709161 0.001427

C -4.603949 0.522490 0.001902

C 3.802053 2.746628 0.000681

N 3.243663 -2.353283 0.000831

C 4.328894 -1.574652 0.002177

N 4.398719 -0.244329 0.002151

C 3.182067 0.302771 0.000571

C 1.964529 -0.372235 -0.000953

C 2.026464 -1.781177 -0.000778

N 0.940446 -2.570573 -0.002083

N 0.920593 0.531580 -0.002225

C 1.493150 1.709752 -0.001514

N 2.860435 1.643045 0.000243

H -4.391879 -2.536546 -0.879409

H -4.514827 2.638759 0.002329

H -5.682182 0.449355 0.003188

H 4.440071 2.698402 0.881737

H 5.277194 -2.099993 0.003473

H 1.089258 -3.563731 -0.001951

H 0.956178 2.645514 -0.001983

H 3.247545 3.681674 0.006790

H 4.433114 2.705886 -0.885821

H -4.391871 -2.535368 0.883671

H -5.722222 -1.753919 0.001610

H -0.881954 0.459379 -0.002481

H -0.005207 -2.207660 -0.003547

S16: m^5^U:G tWH

E = -1075.72397216 A.U.

C 4.951194 -2.515692 -0.019347

N 4.210167 -1.259084 -0.008704

C 2.817191 -1.353931 -0.015280

O 2.252989 -2.427316 -0.029483

N 2.162443 -0.138766 -0.004426

C 2.742860 1.125124 0.007144

O 2.055098 2.139264 0.012270

C 4.201281 1.139173 0.012403

C 4.901379 2.462385 0.026350

C 4.846008 -0.044086 0.004325

C -2.880176 3.251400 -0.020485

N -3.964452 -1.671576 0.018737

C -4.803534 -0.594985 0.003753

N -6.151674 -0.854765 0.065013

N -4.384320 0.641128 -0.017969

C -3.032835 0.740099 -0.006595

C -2.083535 -0.274161 -0.000261

C -2.528304 -1.638682 0.017603

O -1.913517 -2.681760 0.037414

N -0.814686 0.264372 -0.001076

C -0.995875 1.559305 -0.008327

N -2.325399 1.911155 -0.012179

H 4.690469 -3.117486 0.849485

H 5.983665 2.334247 0.028997

H 4.624278 3.060357 -0.843495

H 4.618224 3.045175 0.904511

H 5.926432 -0.096505 0.007636

H -3.507455 3.409422 0.855564

H -6.483595 -1.702218 -0.365222

H -0.196054 2.283091 -0.010157

H 6.014718 -2.292407 -0.004176

H 4.710427 -3.092039 -0.911081

H -2.061265 3.965902 -0.009479

H -3.483518 3.409538 -0.913373

H 1.132937 -0.181695 -0.005513

H -4.343012 -2.604527 0.092154

H -6.721294 -0.045298 -0.120926

S16 unmodified: U:G tWH

E = -1036.39154159 A.U.

C -5.375755 -1.975466 0.004447

N -4.548324 -0.773551 0.000682

C -3.159395 -0.967947 0.008702

O -2.681751 -2.081407 0.018812

N -2.413931 0.192407 0.004211

C -2.896298 1.501967 -0.004826

O -2.134205 2.458957 -0.006198

C -4.342658 1.600322 -0.012021

H -4.790228 2.579787 -0.019712

C -5.089825 0.480997 -0.009138

C 2.870470 3.179462 0.003467

N 3.568993 -1.814411 -0.010000

C 4.489550 -0.806322 -0.004028

N 5.812969 -1.170777 -0.067496

N 4.168278 0.458896 0.011290

C 2.828650 0.663352 0.003110

C 1.803504 -0.274107 0.004983

C 2.139915 -1.669016 -0.005151

O 1.444973 -2.660763 -0.016053

N 0.580385 0.361195 0.006473

C 0.861306 1.638201 0.006261

N 2.214172 1.885835 0.004527

H -5.160208 -2.586781 -0.869922

H -6.170393 0.515610 -0.014355

H 3.497816 3.288150 -0.880056

H 6.079334 -2.040407 0.363932

H 0.119527 2.421433 0.006676

H -6.420894 -1.677480 -0.005046

H -5.172380 -2.573301 0.891023

H 2.108521 3.954504 0.000409

H 3.494228 3.291718 0.889177

H -1.390596 0.068921 0.007334

H 3.873498 -2.774569 -0.077081

H 6.445058 -0.407694 0.112489

S17: m^5^U:G cWW

E = -1075.73421432 A.U.

C -6.248381 0.112227 0.050942

N -1.242803 0.731612 -0.052122

C -2.235002 1.665060 -0.040825

N -1.836786 2.971100 -0.109302

N -3.512487 1.373264 -0.007835

C -3.736716 0.040549 0.000546

C -2.811225 -1.001139 -0.008106

C -1.421360 -0.667835 -0.031267

O -0.436630 -1.401672 -0.033024

N -3.453302 -2.220199 0.013157

C -4.719977 -1.920992 0.033399

N -4.964534 -0.559374 0.026814

C 3.862716 2.764295 0.043325

N 3.690727 1.314753 0.024111

C 2.400503 0.825603 0.013155

O 1.438407 1.599221 0.019003

N 2.278818 -0.532302 -0.005205

C 3.328045 -1.466541 -0.009943

O 3.101845 -2.658537 -0.026097

C 4.668460 -0.878244 0.006351

C 5.842365 -1.805625 0.003043

C 4.776081 0.461135 0.022313

H -6.363278 0.693442 0.965494

H -0.893526 3.196798 0.158012

H -5.532840 -2.629592 0.052057

H 3.364884 3.216816 -0.811938

H 5.812415 -2.469105 0.868589

H 5.823326 -2.448890 -0.877926

H 6.782362 -1.254926 0.015310

H 5.737640 0.954833 0.034181

H 4.925290 2.986727 0.003573

H 3.437574 3.189808 0.951066

H -6.341844 0.784493 -0.800658

H -7.035635 -0.636403 0.003218

H -2.545117 3.646950 0.117806

H 1.313878 -0.907702 -0.015795

H -0.264598 1.041736 -0.066986

S17 unmodified U:G cWW

E = -1036.40069105 A.U.

C -5.923056 0.385992 0.032088

N -0.888292 0.701425 -0.043305

C -1.822568 1.693340 -0.036275

N -1.347447 2.973432 -0.105190

N -3.115296 1.478852 -0.007763

C -3.419958 0.162341 0.000921

C -2.559287 -0.933344 -0.003794

C -1.151983 -0.685101 -0.022719

O -0.214503 -1.478092 -0.022037

N -3.273836 -2.111311 0.016598

C -4.520121 -1.735969 0.032161

N -4.681847 -0.362051 0.024929

C 4.355289 2.381162 0.039763

N 4.088971 0.945611 0.021966

C 2.763289 0.539389 0.014879

O 1.860569 1.379141 0.023528

N 2.547447 -0.805502 -0.002694

C 3.531279 -1.816580 -0.011912

O 3.221487 -2.987421 -0.027197

C 4.891227 -1.300833 -0.000868

C 5.109128 0.022870 0.015020

H -5.962088 1.052665 0.892489

H -5.374446 -2.393998 0.049729

H 3.897575 2.863191 -0.821742

H 5.704379 -2.007303 -0.006830

H 6.104335 0.443406 0.022843

H 0.106584 0.949743 -0.058382

H 1.557629 -1.110929 -0.009532

H -6.017509 0.983681 -0.873919

H -6.753067 -0.314488 0.085435

H 5.430715 2.532011 0.011100

H 3.949379 2.835095 0.942214

H -0.394711 3.145188 0.168259

H -2.016535 3.689914 0.116606

S18: s^4^U:A tWH

E = -1284.09273593 A.U.

C 4.341423 -2.468240 0.001519

N 3.777238 -1.120969 0.000683

C 2.392004 -1.011047 0.000460

O 1.683423 -2.007020 0.000924

N 1.894164 0.269168 -0.000317

C 2.629693 1.436101 -0.000628

S 1.912222 2.939183 -0.001223

C 4.050934 1.244262 -0.000368

C 4.566400 -0.001367 0.000279

C -3.798591 2.750824 0.001562

N -3.463751 -2.363559 -0.000761

C -4.512898 -1.538184 -0.000025

N -4.521002 -0.206689 0.000565

C -3.280586 0.284307 0.000387

C -2.091656 -0.441758 -0.000320

C -2.221962 -1.846643 -0.001001

N -1.176367 -2.688092 -0.002141

N -1.007652 0.419638 -0.000281

C -1.536451 1.619260 0.000408

N -2.904039 1.608911 0.000859

H 4.013644 -3.014937 0.883560

H 4.690584 2.109973 -0.000658

H 5.631141 -0.184180 0.000510

H -4.435072 2.732944 -0.881779

H -5.484062 -2.020060 0.000122

H -1.373709 -3.672623 -0.001377

H -0.961865 2.532143 0.000642

H -3.205219 3.661671 0.001279

H -4.433957 2.732788 0.885708

H 4.013538 -3.016050 -0.879789

H 5.424786 -2.388779 0.001394

H 0.854317 0.328275 -0.000507

H -0.217074 -2.365867 -0.000817

S18 unmodified U:A tWH

E = -961.133821628 A.U.

C -4.654319 -1.954113 0.001729

N -3.942036 -0.679376 0.000744

C -2.550539 -0.725473 -0.000826

O -1.955696 -1.796285 -0.001544

N -1.922718 0.486554 -0.001517

C -2.519531 1.750859 -0.000343

O -1.836256 2.761144 -0.000836

C -3.970702 1.709161 0.001427

C -4.603949 0.522490 0.001902

C 3.802053 2.746628 0.000681

N 3.243663 -2.353283 0.000831

C 4.328894 -1.574652 0.002177

N 4.398719 -0.244329 0.002151

C 3.182067 0.302771 0.000571

C 1.964529 -0.372235 -0.000953

C 2.026464 -1.781177 -0.000778

N 0.940446 -2.570573 -0.002083

N 0.920593 0.531580 -0.002225

C 1.493150 1.709752 -0.001514

N 2.860435 1.643045 0.000243

H -4.391879 -2.536546 -0.879409

H -4.514827 2.638759 0.002329

H -5.682182 0.449355 0.003188

H 4.440071 2.698402 0.881737

H 5.277194 -2.099993 0.003473

H 1.089258 -3.563731 -0.001951

H 0.956178 2.645514 -0.001983

H 3.247545 3.681674 0.006790

H 4.433114 2.705886 -0.885821

H -4.391871 -2.535368 0.883671

H -5.722222 -1.753919 0.001610

H -0.881954 0.459379 -0.002481

H -0.005207 -2.207660 -0.003547

S19: H2U:U tWW

E = -909.828871621 A.U.

C 3.414419 2.542971 0.224415

N 3.183274 1.118039 0.048598

C 1.903235 0.651031 0.057955

O 0.921303 1.386714 0.087044

N 1.744825 -0.732706 0.031115

C 2.724013 -1.678874 -0.202915

O 2.477021 -2.862508 -0.245794

C 4.103355 -1.101057 -0.438305

C 4.300752 0.215195 0.297009

C -3.513175 -2.523713 0.241000

N -3.322024 -1.082078 0.101939

C -2.014392 -0.611663 0.110057

O -1.073224 -1.387015 0.231309

N -1.871046 0.741132 -0.024356

C -2.895206 1.693132 -0.170625

O -2.636897 2.874014 -0.283367

C -4.226752 1.110436 -0.168480

C -4.379841 -0.217871 -0.035349

H 2.504659 3.079867 -0.020766

H 4.209413 -0.948423 -1.515871

H 4.838942 -1.843021 -0.136485

H 5.211041 0.705972 -0.045935

H 4.415850 0.037901 1.373892

H -3.011028 -3.052776 -0.566544

H -5.073252 1.767923 -0.274950

H -5.354200 -0.685109 -0.029122

H -4.578450 -2.735330 0.210120

H -3.097926 -2.872958 1.184350

H 4.220158 2.867257 -0.434791

H 3.692427 2.775824 1.257028

H 0.774624 -1.054920 0.104285

H -0.901532 1.079915 -0.008378

S19 unmodified U:U tWW

E = -908.616062842 A.U.

C 3.429657 2.549241 0.000290

N 3.251899 1.098842 0.000125

C 1.947867 0.619810 0.000447

O 0.998230 1.394035 0.000884

N 1.817986 -0.741377 0.000231

C 2.852396 -1.694152 -0.000325

O 2.603577 -2.882371 -0.000492

C 4.179090 -1.101418 -0.000651

C 4.318928 0.235094 -0.000429

C -3.429678 -2.549233 0.000364

N -3.251913 -1.098835 -0.000047

C -1.947879 -0.619819 0.000282

O -0.998252 -1.394057 0.000747

N -1.817975 0.741369 0.000043

C -2.852371 1.694147 -0.000357

O -2.603547 2.882367 -0.000537

C -4.179075 1.101433 -0.000507

C -4.318935 -0.235076 -0.000342

H 2.966103 2.988654 -0.880703

H 5.033026 -1.757897 -0.001079

H 5.289666 0.709757 -0.000668

H -2.964205 -2.988918 -0.879457

H -5.032996 1.757931 -0.000789

H -5.289676 -0.709731 -0.000465

H -4.494218 -2.766478 -0.001963

H -2.968249 -2.988135 0.882743

H 4.494198 2.766495 0.000195

H 2.966300 2.988402 0.881504

H 0.852567 -1.087568 0.000476

H -0.852554 1.087545 0.000300

S20 H2U:G cHS

E = -1037.60115923 A.U.

C 0.358804 2.466899 -0.546209

N 3.236840 -1.610569 0.170251

C 1.885639 -1.532958 -0.031547

N 1.187279 -2.702293 -0.021656

N 1.256554 -0.390994 -0.186092

C 2.084117 0.680498 -0.135470

C 3.460196 0.718079 0.064402

C 4.162797 -0.518658 0.248301

O 5.336481 -0.752751 0.444324

N 3.920288 2.016000 0.037632

C 2.856626 2.735667 -0.174967

N 1.700883 1.984824 -0.291347

C -4.539151 1.660251 1.248892

N -3.725334 0.591798 0.688805

C -3.830788 0.330439 -0.646373

O -4.440890 1.006594 -1.450039

N -3.147675 -0.823550 -1.085968

C -2.228680 -1.573363 -0.397547

O -1.622492 -2.482906 -0.935805

C -2.019010 -1.146307 1.032318

C -3.234329 -0.432192 1.605666

H -0.287328 2.303287 0.316496

H 0.235041 -2.666834 -0.375274

H 2.830631 3.810255 -0.262337

H -4.817628 2.342604 0.452990

H -1.764213 -2.025997 1.619792

H -1.140183 -0.495201 1.026911

H -4.030502 -1.152276 1.833192

H -2.969009 0.056848 2.542066

H -3.968912 2.195415 2.008286

H -5.451060 1.266810 1.708470

H 0.403439 3.533150 -0.754835

H -0.071586 1.954936 -1.405256

H 3.670147 -2.510459 0.316233

H 1.686388 -3.554728 -0.207460

H -3.249196 -1.011951 -2.073282

S20 unmodified U:G cHS

E = -1036.38981517 A.U.

C -1.287908 2.733579 -0.692755

N -3.180604 -1.851284 0.203563

C -1.900531 -1.499531 -0.135752

N -1.011813 -2.505560 -0.313295

N -1.536748 -0.248560 -0.319652

C -2.558762 0.626354 -0.167982

C -3.884147 0.380540 0.175993

C -4.296342 -0.974907 0.397548

O -5.373712 -1.445836 0.696430

N -4.601667 1.555671 0.224587

C -3.738220 2.479829 -0.080694

N -2.471998 1.982709 -0.335367

C 5.169736 2.048057 0.572315

N 4.263495 0.934252 0.296783

C 4.851011 -0.307244 0.062550

O 6.050712 -0.476930 0.080782

N 3.945189 -1.320962 -0.189688

C 2.552948 -1.237909 -0.231970

O 1.891350 -2.241960 -0.462140

C 2.040033 0.089827 0.014661

C 2.903023 1.093634 0.264809

H -0.761441 2.232169 -1.502689

H -0.026211 -2.281263 -0.424436

H -3.943068 3.536618 -0.146825

H 5.775048 1.832709 1.450374

H 0.971208 0.241372 -0.010010

H 2.560214 2.100113 0.457990

H 4.577123 2.941631 0.746087

H 5.838169 2.209125 -0.271169

H -1.584451 3.726775 -1.022716

H -0.611650 2.832824 0.158344

H -3.419676 -2.826725 0.303660

H -1.219710 -3.414471 0.058889

H 4.351626 -2.230308 -0.358934

S21 H2U:G tWS

E = -1037.60930745 A.U.

C -1.088265 2.541080 -1.177934

N -2.643709 -2.024518 0.352313

C -1.399269 -1.643977 -0.067575

N -0.462863 -2.615376 -0.180390

N -1.108067 -0.399101 -0.387324

C -2.179797 0.435513 -0.308925

C -3.476583 0.148202 0.108337

C -3.800881 -1.192651 0.495859

O -4.838168 -1.687415 0.883760

N -4.271571 1.269087 0.035581

C -3.479977 2.198981 -0.415393

N -2.191207 1.759156 -0.648776

C 5.001938 -1.570484 -0.579487

N 4.063876 -0.562582 -0.110070

C 2.728895 -0.783115 -0.250482

O 2.258912 -1.842743 -0.652517

N 1.882096 0.273864 0.092183

C 2.235007 1.427825 0.761412

O 1.422495 2.280889 1.046981

C 3.698660 1.516430 1.130732

C 4.575906 0.781425 0.130086

H -0.316962 2.683997 -0.423689

H -0.594260 -3.483077 0.306668

H -3.756387 3.223972 -0.604629

H 4.481805 -2.516846 -0.677202

H 0.883243 0.117296 -0.096749

H 3.810199 1.074631 2.124743

H 4.636012 1.341156 -0.811495

H 5.591102 0.690083 0.514210

H -0.657091 2.039047 -2.043667

H -1.473114 3.509105 -1.490735

H 5.421854 -1.297807 -1.552181

H 5.817963 -1.673523 0.136195

H 0.494370 -2.343191 -0.397040

H -2.825469 -2.997296 0.550240

H 3.967130 2.567527 1.205136

S21 unmodified: U:G tWS

E = -1036.39635924 A.U.

C -1.097923 2.562633 -1.168437

N -2.538221 -2.053830 0.327682

C -1.311696 -1.645302 -0.115626

N -0.366234 -2.603253 -0.278529

N -1.045068 -0.390123 -0.413027

C -2.127665 0.427213 -0.293217

C -3.408076 0.111348 0.153111

C -3.702671 -1.241618 0.521745

O -4.721561 -1.760038 0.926446

N -4.222709 1.219489 0.119709

C -3.458346 2.169205 -0.337590

N -2.169539 1.755132 -0.611872

C 5.034623 -1.560879 -0.420987

N 4.114307 -0.486462 -0.052388

C 2.760246 -0.710967 -0.262961

O 2.365744 -1.770679 -0.732689

N 1.926668 0.317926 0.088813

C 2.299921 1.544407 0.662333

O 1.462140 2.381419 0.942488

C 3.729474 1.683704 0.861613

C 4.557271 0.688225 0.501131

H -0.299284 2.702095 -0.442683

H -0.465342 -3.476408 0.206777

H -3.756525 3.192122 -0.503383

H 4.920643 -1.809985 -1.473653

H 0.923546 0.162529 -0.085217

H 4.099067 2.597418 1.295713

H 5.627860 0.757866 0.629198

H -0.696041 2.083704 -2.060994

H -1.508209 3.530865 -1.446076

H 6.050153 -1.224348 -0.232900

H 4.831560 -2.454939 0.165600

H 0.578783 -2.312547 -0.515567

H -2.702168 -3.033956 0.503806

S22: Ψ:A cWW

E = -961.143893287 A.U.

C -5.302290 -2.036833 -0.000449

N -4.065726 1.534270 -0.002397

C -2.688609 1.440140 -0.001032

O -1.978852 2.438993 -0.001270

N -2.214989 0.158754 0.000606

C -2.975159 -1.014408 0.000922

O -2.430405 -2.104273 0.002442

C -4.429374 -0.821860 -0.000666

C -4.898485 0.438237 -0.002231

C 5.494564 -1.744472 -0.014871

N 0.652494 -0.071633 0.001545

C 1.164055 -1.313392 0.004603

N 2.441652 -1.669863 0.004705

C 3.249160 -0.603504 0.002662

C 2.865266 0.734557 -0.000057

C 1.480256 0.992002 -0.000115

N 0.968541 2.230515 -0.001816

N 3.965899 1.565818 0.001181

C 4.981725 0.743616 0.003876

N 4.622746 -0.585696 0.007759

H -5.099620 -2.659206 -0.873282

H -5.955787 0.664052 -0.003443

H 5.147019 -2.477937 0.709543

H 0.428767 -2.109690 0.006768

H 1.596883 3.013053 -0.002578

H 6.019680 1.037447 0.007508

H -5.101776 -2.657377 0.874190

H -6.357308 -1.764654 -0.002017

H 5.501218 -2.208237 -1.001369

H 6.504690 -1.434020 0.242225

H -0.036816 2.377039 -0.001540

H -1.177392 0.052803 0.001261

H -4.436481 2.469314 -0.003577

S22 unmodified U:A cWW

E =: -961.133399436 A.U.

C -5.155743 -2.008248 -0.002228

C -2.718976 1.493870 -0.000376

O -1.959360 2.458631 -0.000288

N -2.223790 0.196720 0.001387

C -2.952277 -0.968965 0.000885

O -2.451314 -2.076766 0.002156

C -4.896767 0.452577 -0.002576

C 5.487578 -1.737233 -0.010803

N 0.643214 -0.070222 0.003061

C 1.156965 -1.310631 0.005190

N 2.434828 -1.666519 0.004720

C 3.241012 -0.598770 0.002400

C 2.855607 0.738640 0.000131

C 1.469926 0.995527 0.000914

N 0.956289 2.231816 -0.000448

N 3.955386 1.571076 -0.000814

C 4.972102 0.750199 0.000428

N 4.614537 -0.579611 0.004315

H -4.938588 -2.609603 -0.882951

H -5.977467 0.474017 -0.003952

H 5.223285 -2.414880 0.798939

H 0.423568 -2.108628 0.007154

H 1.583863 3.015085 -0.002183

H 6.009845 1.044822 0.001491

H -4.940938 -2.609564 0.879085

H -6.203861 -1.721576 -0.003662

H 5.399119 -2.274931 -0.954408

H 6.515184 -1.405658 0.118344

H -0.051385 2.376759 0.000147

H -1.186759 0.082702 0.002463

H -4.623350 2.556017 -0.003309

C -4.163558 1.582222 -0.002243

N -4.340796 -0.796776 -0.001141

S23 Ψ:A cHW

E = -961.146445622 A.U.

C 5.826076 -1.586510 0.007035

N 0.864461 -0.271116 -0.008651

C 1.474123 -1.467941 -0.007820

N 2.770601 -1.736196 -0.003567

C 3.501656 -0.614325 0.000146

C 3.019005 0.690061 -0.000256

C 1.618235 0.851349 -0.004884

N 1.032698 2.052872 -0.005849

N 4.053774 1.600314 0.004471

C 5.128219 0.856365 0.007595

N 4.869226 -0.496173 0.005318

C -5.068450 -2.348644 0.002603

N -2.043549 -0.067943 -0.007776

C -2.553189 1.202638 -0.003037

O -1.858369 2.212838 -0.004763

N -3.928162 1.255112 0.004076

C -4.827724 0.176263 0.006260

O -6.026796 0.380686 0.012442

C -4.189921 -1.136915 0.000623

C -2.842433 -1.182924 -0.006047

H 5.688522 -2.211010 0.888425

H 0.810711 -2.325782 -0.011031

H 1.618322 2.868659 -0.001507

H 6.141402 1.226413 0.011724

H -4.476302 -3.263058 -0.002492

H -2.305407 -2.121693 -0.010471

H -5.726176 -2.354201 -0.868019

H -5.717007 -2.358027 0.880052

H 6.831192 -1.171990 0.014231

H 5.698783 -2.205199 -0.880015

H 0.018645 2.152335 -0.006623

H -1.011455 -0.157820 -0.012855

H -4.336830 2.178590 0.007688

S23 unmodified U:A cHW

E = -961.124793530 A.U.

C 6.240497 1.291437 0.005080

N 1.160248 0.540148 0.011591

C 1.899083 1.656637 0.008974

N 3.222328 1.782271 0.003130

C 3.821663 0.586874 -0.001639

C 3.196167 -0.655916 -0.000027

C 1.785672 -0.653534 0.006942

N 1.069128 -1.785971 0.010011

N 4.124614 -1.676742 -0.008886

C 5.275321 -1.057549 -0.015264

N 5.169080 0.315428 -0.013972

C -5.645393 2.005731 -0.008532

C -2.653599 -1.049324 0.004170

O -1.879224 -1.997252 0.006809

N -4.031300 -1.282370 0.001410

C -5.051722 -0.350331 -0.002879

O -6.226998 -0.650840 -0.005623

C -3.276151 1.276899 -0.000519

H 6.057273 2.055869 -0.747519

H 1.336419 2.583982 0.012012

H 1.558343 -2.662557 0.002175

H 6.240965 -1.538410 -0.025127

H -5.159971 2.977492 -0.009271

H -3.053161 2.334301 -0.001363

H -6.278756 1.912918 0.871572

H -6.274113 1.909265 -0.891563

H 7.180762 0.790154 -0.212931

H 6.309527 1.775235 0.979542

H 0.053861 -1.779337 0.010146

H -1.250728 0.617550 0.006059

H -4.328529 -2.247773 0.001808

C -2.298804 0.351005 0.003393

N -4.613888 0.970731 -0.003745

S24 Ψ:U tWW

E = -908.628720129 A.U.

C -5.564069 -0.633954 -0.004109

N -4.253191 0.009409 -0.002025

C -3.138039 -0.837046 -0.000684

O -3.257111 -2.043644 -0.001299

N -1.922544 -0.185677 0.001291

C -1.705663 1.187675 0.002541

O -0.568291 1.648191 0.004646

C -2.913405 1.982745 0.001168

C -4.111999 1.368853 -0.000992

C 5.708328 0.563634 -0.004369

N 2.825033 -1.878135 0.001304

C 1.688928 -1.097799 0.002648

O 0.572147 -1.602353 0.004511

N 1.931216 0.247601 0.001752

C 3.187753 0.873542 -0.000637

O 3.276358 2.084879 -0.001471

C 4.338508 -0.037440 -0.001969

C 4.100712 -1.360108 -0.000928

H -5.675241 -1.263106 -0.885134

H -2.825048 3.055872 0.001926

H -5.037740 1.926801 -0.002064

H 5.849662 1.201189 -0.878336

H 4.894540 -2.093993 -0.001796

H -5.678354 -1.262659 0.876853

H -6.329327 0.137453 -0.005653

H 5.852598 1.201525 0.868881

H 6.477738 -0.207788 -0.005501

H -1.086083 -0.782452 0.002493

H 1.096631 0.848998 0.002899

H 2.665887 -2.871420 0.002072

S24 unmodified U:U tWW

E = -908.618315551 A.U.

C -5.556751 -0.617358 -0.005801

N -4.242266 0.018271 -0.002627

C -3.131734 -0.835713 -0.001057

O -3.260338 -2.040988 -0.002304

N -1.911867 -0.192405 0.001931

C -1.688310 1.178714 0.003666

O -0.547934 1.634374 0.006412

C -2.890220 1.982267 0.001955

C -4.092956 1.376314 -0.001041

C 5.556580 0.617823 -0.005810

C 1.688483 -1.178999 0.003786

O 0.548192 -1.634839 0.006562

N 1.911796 0.192198 0.002124

C 3.131517 0.835710 -0.001019

O 3.259933 2.041019 -0.002229

C 4.093169 -1.376122 -0.001144

H -5.671251 -1.245134 -0.887401

H -2.794541 3.054755 0.003114

H -5.015122 1.940226 -0.002410

H 5.670277 1.246975 -0.886515

H 5.015436 -1.939872 -0.002578

H -5.674893 -1.246430 0.874397

H -6.317586 0.158479 -0.006800

H 5.675274 1.245566 0.875279

H 6.317552 -0.157874 -0.008569

H -1.076270 -0.794640 0.003476

H 1.076030 0.794265 0.003659

H 2.795115 -3.054820 0.003111

C 2.890551 -1.982313 0.001973

N 4.242219 -0.018058 -0.002764

S25 Ψ:G tWW/Bs

E = -1036.40763558 A.U.

C -5.904312 -1.260088 -0.243093

N -1.030200 -0.103153 0.320137

C -1.664367 -1.301532 0.479373

N -0.888883 -2.344814 0.889862

N -2.951365 -1.471620 0.293516

C -3.577857 -0.327412 -0.062638

C -3.041171 0.942509 -0.256759

C -1.631606 1.125320 -0.066957

O -0.938023 2.121629 -0.187500

N -4.026446 1.834369 -0.622459

C -5.117805 1.124364 -0.646088

N -4.916306 -0.203157 -0.315409

C 2.239253 1.959532 1.170071

N 5.393265 0.603207 -0.420779

C 5.150287 -0.730647 -0.685464

O 5.956324 -1.474604 -1.196386

N 3.877883 -1.121484 -0.298805

C 2.885450 -0.342338 0.286022

O 1.802035 -0.852498 0.559907

C 3.241602 1.048932 0.530431

C 4.477580 1.444351 0.163049

H -5.939065 -1.684458 0.759641

H -1.309178 -3.253630 0.812153

H -6.101775 1.490973 -0.892274

H 1.338219 2.066699 0.562426

H 4.817933 2.459513 0.310253

H 1.925353 1.567600 2.139234

H 2.665095 2.950039 1.325134

H -5.667146 -2.056917 -0.947266

H -6.879126 -0.845435 -0.489114

H 3.649056 -2.088842 -0.477806

H 0.110287 -2.274923 0.780118

H 6.305128 0.936519 -0.686809

H -0.025437 -0.074731 0.466011

S25 unmodified U:G tWW/BS

E = -1036.39724646 A.U.

C -5.880061 -1.204557 -0.370239

N -1.004290 -0.148316 0.385028

C -1.662704 -1.339071 0.493286

N -0.915551 -2.404119 0.901355

N -2.945011 -1.481402 0.263388

C -3.543204 -0.319055 -0.081478

C -2.977883 0.945877 -0.224651

C -1.573558 1.097604 0.013012

O -0.858477 2.085715 -0.059638

N -3.935327 1.865379 -0.594379

C -5.038613 1.177157 -0.670318

N -4.871863 -0.161984 -0.368982

C 2.246079 1.863264 1.144396

C 5.129836 -0.708502 -0.747871

O 5.874922 -1.515640 -1.261433

N 3.861704 -1.112420 -0.277816

C 2.897052 -0.346098 0.323214

O 1.823433 -0.814159 0.690499

C 4.438439 1.464612 0.057874

H -5.436483 -2.103251 0.049372

H -1.342335 -3.305902 0.785537

H -6.006723 1.571998 -0.934813

H 1.337673 1.958490 0.550376

H 4.590492 2.519896 0.230099

H 1.988562 1.461740 2.122445

H 2.701024 2.841615 1.268134

H -6.221130 -1.418212 -1.383774

H -6.733762 -0.908929 0.238571

H 3.632128 -2.087725 -0.404576

H 0.087335 -2.345721 0.826951

H 6.313532 1.117341 -0.863003

H -0.003253 -0.141901 0.557453

C 5.374839 0.702661 -0.536150

N 3.226423 0.984272 0.490745

S26 Ψ:G cWW

E = -1036.41448715 A.U.

C -4.478921 2.456995 0.056217

N -4.770850 -1.303771 0.008104

C -3.464830 -1.783607 -0.012464

O -3.213012 -2.965385 -0.031082

N -2.514698 -0.776878 -0.009109

C -2.761234 0.582925 0.011792

O -1.830904 1.395487 0.010849

C -4.159291 0.994603 0.033450

C -5.094723 0.024448 0.030006

C 5.924888 0.401049 0.061431

N 0.890418 0.700010 -0.055763

C 1.821013 1.695214 -0.046119

N 1.340901 2.971546 -0.118311

N 3.115094 1.484950 -0.010343

C 3.423204 0.169797 0.001491

C 2.565771 -0.928860 -0.007307

C 1.158559 -0.683419 -0.033283

O 0.222472 -1.480420 -0.035828

N 3.284014 -2.104821 0.015921

C 4.529009 -1.725585 0.037712

N 4.686617 -0.351130 0.029263

H -4.047398 2.938719 0.935064

H -6.153355 0.244508 0.044008

H 5.944340 1.128519 -0.748235

H 2.001840 3.695429 0.102313

H 5.385283 -2.381038 0.057558

H 6.033142 0.932018 1.007207

H 6.757317 -0.288546 -0.057283

H -4.060161 2.962717 -0.815183

H -5.555922 2.620788 0.066742

H -0.107019 0.947531 -0.069165

H -1.523491 -1.084668 -0.022659

H -5.483912 -2.013927 0.005650

H 0.379748 3.139781 0.126585

S26 unmodified G:U cWW

E = -1036.40069344 A.U.

C -4.355483 2.381109 0.037891

C -3.531188 -1.816641 -0.009362

O -3.221340 -2.987481 -0.023596

N -2.547429 -0.805493 -0.001367

C -2.763374 0.539433 0.014887

O -1.860731 1.379264 0.022288

C -5.109177 0.022729 0.016129

C 5.922948 0.385841 0.045533

N 0.888351 0.701567 -0.044361

C 1.822751 1.693442 -0.037205

N 1.347719 2.973570 -0.106304

N 3.115402 1.478823 -0.008021

C 3.420050 0.162278 -0.000365

C 2.559284 -0.933302 -0.005735

C 1.151978 -0.684910 -0.024644

O 0.214462 -1.477883 -0.024152

N 3.273750 -2.111339 0.012043

C 4.520125 -1.736151 0.027366

N 4.681969 -0.362257 0.019789

H -3.945232 2.836994 0.937339

H -6.104427 0.443176 0.023754

H 5.943323 1.109634 -0.767421

H 2.016632 3.690019 0.116102

H 5.374335 -2.394403 0.041731

H 6.035158 0.920414 0.988757

H 6.752564 -0.307300 -0.071952

H -3.902051 2.861409 -0.826894

H -5.431056 2.531763 0.014139

H -0.106488 0.949955 -0.059121

H -1.557609 -1.110807 -0.009065

H -5.704274 -2.007503 -0.003676

H 0.394810 3.145291 0.166612

C -4.891170 -1.300967 0.001438

N -4.089087 0.945557 0.022189

S 27 Ψ:C cS(r):W

E = -1270.57692416 A.U.

C 2.391141 4.327209 0.052612

C 1.601548 3.147385 -0.461865

O 2.502947 2.093533 -0.847318

C 0.683898 2.462928 0.556951

O -0.502112 3.150605 0.849419

C 0.454165 1.087461 -0.105570

O -0.618250 1.193781 -1.013308

C 1.785581 0.853998 -0.870418

N 4.698378 -0.967991 0.701587

C 4.372092 -2.311030 0.686910

O 5.096452 -3.183698 1.112172

N 3.125968 -2.546085 0.131915

C 2.222531 -1.613061 -0.380904

O 1.154673 -2.000758 -0.838874

C 2.668190 -0.230913 -0.313198

C 3.881842 0.021539 0.216597

C -6.099091 0.439865 0.856100

N -4.980706 -0.384475 0.411226

C -3.735499 0.272565 0.199786

O -3.668952 1.473207 0.395783

N -2.685771 -0.495702 -0.215797

C -2.815759 -1.797566 -0.421775

N -1.741465 -2.489121 -0.833918

C -4.063747 -2.469204 -0.220644

C -5.106402 -1.712638 0.195219

H 3.044160 4.732744 -0.720569

H 1.702776 5.110883 0.370192

H 0.996362 3.446096 -1.326185

H 1.239253 2.338090 1.491989

H -1.176519 2.820674 0.234723

H 0.259978 0.306660 0.630419

H 1.514895 0.597984 -1.899108

H 4.264911 1.028892 0.268877

H -5.854622 0.924384 1.799259

H -0.828919 -2.057191 -0.912672

H -4.176581 -3.528135 -0.388986

H -6.085475 -2.134924 0.374502

H -1.797702 -3.482936 -0.953256

H 2.841354 -3.514275 0.095353

H 5.601313 -0.748020 1.088333

H 3.004196 4.038493 0.908680

H -1.345211 0.592687 -0.733395

H -6.975266 -0.192105 0.980306

H -6.304720 1.219016 0.124653

S27unmodifeid U:C cS(r)W

E = -1270.57063244 A.U.

C 2.670335 4.111190 -0.003867

C 1.729733 3.039393 -0.498030

O 2.497875 1.925412 -1.024775

C 0.831155 2.388854 0.560568

O -0.288500 3.146595 0.925701

C 0.474375 1.052092 -0.119455

O -0.602910 1.269748 -0.995021

C 1.751228 0.738487 -0.930036

C 4.225924 -2.348627 0.810462

O 4.842645 -3.294441 1.256992

N 3.004636 -2.557285 0.135078

C 2.172496 -1.619044 -0.422380

O 1.124346 -1.920097 -0.979473

C 3.792515 -0.015534 0.325319

C -6.012081 0.548547 0.967821

N -4.929130 -0.286249 0.458202

C -3.673731 0.345385 0.237996

O -3.566925 1.534645 0.480609

N -2.656453 -0.432188 -0.239405

C -2.825849 -1.722902 -0.487909

N -1.781091 -2.428357 -0.949463

C -4.086466 -2.367483 -0.280094

C -5.096752 -1.600906 0.193442

H 3.305280 4.480193 -0.809219

H 2.087054 4.946100 0.385423

H 1.093793 3.431927 -1.297091

H 1.423014 2.207168 1.463353

H -1.010455 2.879240 0.334608

H 0.246466 0.264194 0.598510

H 1.473094 0.363528 -1.913456

H 4.035807 1.034160 0.326543

H -5.728132 0.987747 1.921978

H -0.855253 -2.023906 -1.020113

H -4.232314 -3.415837 -0.484982

H -6.081702 -2.004275 0.383353

H -1.869689 -3.415533 -1.101146

H 2.697562 -3.515965 0.055499

H 5.514344 -0.683218 1.360903

H 3.308266 3.734745 0.798008

H -1.339242 0.658512 -0.755221

H -6.901173 -0.064860 1.092276

H -6.216705 1.361441 0.273874

C 4.590941 -0.950743 0.875038

N 2.609864 -0.311992 -0.304214

S1’ 5BrC:G cWW

E = -3590.14699751 A.U.

C 3.575945 3.446825 -0.027116

N 3.071513 2.076031 -0.017804

C 1.670887 1.887558 0.005196

O 0.951523 2.885641 0.017122

N 1.201410 0.619893 0.011544

C 2.001542 -0.446010 0.008635

N 1.453737 -1.654714 0.018027

C 3.429081 -0.257907 -0.005681

C 3.904619 1.007865 -0.018995

Br 4.632043 -1.732320 -0.009465

C -6.777300 0.506446 -0.046484

N -1.740086 0.265374 0.032632

C -2.556983 1.365045 0.023388

N -1.953123 2.568813 0.038701

N -3.872938 1.282566 0.003247

C -4.315767 0.011386 -0.004400

C -3.580622 -1.174024 0.005820

C -2.159081 -1.076164 0.026697

O -1.314532 -1.976527 0.041529

N -4.420996 -2.268524 -0.006929

C -5.617890 -1.759015 -0.024378

N -5.627975 -0.374247 -0.024405

H 3.173817 3.983842 -0.883227

H 0.425407 -1.769678 0.027296

H 4.963340 1.219200 -0.031657

H -6.766455 1.170114 0.817306

H -2.547757 3.375608 0.011331

H -6.539846 -2.318568 -0.038248

H -7.682034 -0.096468 -0.021284

H -6.778872 1.116452 -0.949262

H 3.263299 3.968161 0.875456

H 4.661133 3.422525 -0.082598

H 2.045873 -2.466034 0.013355

H -0.721106 0.395730 0.051045

H -0.941612 2.679037 0.031375

S1’ C:G cWW

E = -1016.52629096 A.U.

C 5.198949 -1.871772 0.010027

N 4.388179 -0.658545 0.005011

C 2.982064 -0.807265 -0.001143

O 2.520790 -1.949466 -0.002317

N 2.221281 0.310626 -0.005236

C 2.765148 1.526956 -0.001595

N 1.946908 2.579968 -0.006521

C 4.188393 1.698895 0.006637

C 4.944158 0.578217 0.009638

C -5.550682 -1.464573 0.019213

N -0.707387 -0.050759 -0.008229

C -1.245444 -1.310426 -0.009813

N -0.374358 -2.337560 -0.018070

N -2.544702 -1.540342 -0.004777

C -3.272107 -0.407843 -0.000925

C -2.834463 0.916233 -0.000045

C -1.428808 1.155367 -0.004006

O -0.823429 2.230723 -0.003929

N -3.907799 1.784052 0.003293

C -4.952661 1.008714 0.005113

N -4.638619 -0.339765 0.000343

H 4.979733 -2.467245 0.894157

H 2.327520 3.508175 -0.000055

H 4.643706 2.676356 0.010399

H 6.024470 0.613166 0.015596

H -5.286753 -2.173622 -0.763534

H 0.634706 -2.202067 -0.011331

H -0.760578 -3.262607 -0.009749

H -5.979920 1.337606 0.006180

H -5.514949 -1.981136 0.978793

H -6.561519 -1.101425 -0.152336

H 6.249583 -1.591988 0.005578

H 4.974754 -2.476576 -0.866284

H 0.921731 2.454372 -0.007166

H 0.316823 0.048195 -0.009591

S2’ 5BrU:A cWW

E = -3534.75212754 A.U.

C 3.450366 3.525490 -0.003586

N 2.945056 2.154126 -0.000118

C 1.556802 1.990106 0.006633

O 0.804441 2.944260 0.009984

N 1.130898 0.682739 0.009092

C 1.909005 -0.465076 0.005573

O 1.399697 -1.576449 0.008484

C 3.340759 -0.195172 -0.001770

C 3.786306 1.077161 -0.004198

Br 4.543812 -1.652871 -0.007467

C -6.788440 0.887150 -0.032500

N -1.699022 0.297024 0.008257

C -2.466717 1.399544 0.006964

N -3.790497 1.472237 0.002148

C -4.349929 0.256932 0.000535

C -3.686580 -0.966665 0.002615

C -2.278578 -0.920971 0.007067

N -1.513655 -2.020094 0.010204

N -4.581794 -2.015475 0.004185

C -5.751217 -1.431968 0.002186

N -5.687254 -0.056343 0.003443

H 3.091799 4.056024 -0.883356

H 4.841417 1.305895 -0.009388

H -6.552187 1.738646 0.601326

H -1.920818 2.335656 0.009639

H -1.959681 -2.919254 0.008492

H -6.701073 -1.943238 0.005376

H -6.963935 1.247228 -1.046642

H -7.689025 0.401318 0.336264

H 4.536312 3.496193 -0.009305

H 3.101215 4.057119 0.879298

H -0.499969 -1.948421 0.011111

H 0.092625 0.542162 0.013191

S2’ U:A cWW

E = -961.133397377 A.U.

C 5.487720 -1.737522 -0.014760

N 0.643271 -0.069741 0.001001

C 1.156826 -1.310215 0.004349

N 2.434630 -1.666256 0.004769

C 3.241157 -0.598823 0.002799

C 2.855873 0.738643 -0.000190

C 1.470258 0.995765 -0.000594

N 0.956769 2.232133 -0.002626

N 3.955730 1.570903 0.001407

C 4.972485 0.749982 0.004562

N 4.614775 -0.579775 0.008539

C -5.155801 -2.008364 0.000023

N -4.340943 -0.796857 -0.000259

C -2.952301 -0.969002 0.000464

O -2.451379 -2.076812 0.001510

N -2.223964 0.196707 -0.000086

C -2.719232 1.493938 -0.001158

O -1.959589 2.458643 -0.001498

C -4.163785 1.582170 -0.001812

C -4.896917 0.452444 -0.001361

H -4.623713 2.555907 -0.002632

H 5.132962 -2.476796 0.700153

H 0.423349 -2.108130 0.006466

H 1.584312 3.015417 -0.003057

H 6.010130 1.044904 0.008660

H -4.939259 -2.610250 -0.880484

H -5.977621 0.473926 -0.001813

H -0.050875 2.376951 -0.002209

H -1.186975 0.082867 0.000332

H -6.203940 -1.721768 -0.000802

H -4.940355 -2.609146 0.881550

H 6.494996 -1.428797 0.255389

H 5.505154 -2.193621 -1.004783

S3’ 5BrU:G cWW

E = -3610.01943502 A.U.

C 2.663666 3.371974 -0.046949

N 2.640720 1.910714 -0.024847

C 1.404272 1.287136 -0.008274

O 0.371277 1.958064 -0.011565

N 1.423190 -0.075921 0.012185

C 2.552871 -0.915016 0.015543

O 2.443570 -2.116840 0.033946

C 3.811187 -0.164626 -0.005558

C 3.804301 1.178578 -0.024229

Br 5.432070 -1.132874 -0.004418

C -7.109583 -0.451355 -0.055035

N -2.224923 0.808312 0.052806

C -3.329783 1.606685 0.039417

N -3.105887 2.953497 0.108624

N -4.558132 1.151675 0.004542

C -4.609604 -0.198515 -0.003017

C -3.557403 -1.112140 0.007684

C -2.223401 -0.602401 0.033019

O -1.152992 -1.205973 0.038112

N -4.036456 -2.403811 -0.012791

C -5.331034 -2.270333 -0.034611

N -5.749421 -0.951585 -0.029880

H 2.176543 3.746589 -0.945289

H 4.716952 1.754363 -0.039273

H -7.291993 0.197602 0.800231

H -2.203567 3.303856 -0.165066

H -6.045764 -3.077764 -0.053384

H 3.698695 3.700580 -0.031296

H 2.141337 3.771163 0.819943

H -7.793586 -1.295678 -0.015005

H -7.295197 0.115820 -0.966651

H -1.294688 1.237564 0.072709

H 0.496206 -0.545244 0.025529

H -3.898822 3.529658 -0.114191

3’ –unmodified U:G cWW

E = -1036.40069105 A.U.

C -5.923056 0.385992 0.032088

N -0.888292 0.701425 -0.043305

C -1.822568 1.693340 -0.036275

N -1.347447 2.973432 -0.105190

N -3.115296 1.478852 -0.007763

C -3.419958 0.162341 0.000921

C -2.559287 -0.933344 -0.003794

C -1.151983 -0.685101 -0.022719

O -0.214503 -1.478092 -0.022037

N -3.273836 -2.111311 0.016598

C -4.520121 -1.735969 0.032161

N -4.681847 -0.362051 0.024929

C 4.355289 2.381162 0.039763

N 4.088971 0.945611 0.021966

C 2.763289 0.539389 0.014879

O 1.860569 1.379141 0.023528

N 2.547447 -0.805502 -0.002694

C 3.531279 -1.816580 -0.011912

O 3.221487 -2.987421 -0.027197

C 4.891227 -1.300833 -0.000868

C 5.109128 0.022870 0.015020

H -5.962088 1.052665 0.892489

H -5.374446 -2.393998 0.049729

H 3.897575 2.863191 -0.821742

H 5.704379 -2.007303 -0.006830

H 6.104335 0.443406 0.022843

H 0.106584 0.949743 -0.058382

H 1.557629 -1.110929 -0.009532

H -6.017509 0.983681 -0.873919

H -6.753067 -0.314488 0.085435

H 5.430715 2.532011 0.011100

H 3.949379 2.835095 0.942214

H -0.394711 3.145188 0.168259

H -2.016535 3.689914 0.116606

4’ 5BrU:5BrU cWW

E = -6055.85388208 A.U.

C 2.842200 3.503351 0.000586

N 2.800746 2.041173 0.000137

C 1.552215 1.438757 0.000259

O 0.530551 2.116432 0.000481

N 1.556983 0.071486 0.000107

C 2.669619 -0.786423 -0.000064

O 2.536109 -1.987365 -0.000191

C 3.937439 -0.054998 -0.000049

C 3.949787 1.289831 0.000068

Br 5.544905 -1.046538 -0.000265

C -5.669898 2.303380 -0.000555

N -4.495966 1.432085 -0.000682

C -3.246526 2.058456 -0.000204

O -3.136121 3.265124 -0.000025

N -2.173881 1.190157 -0.000021

C -2.198909 -0.198180 0.000146

O -1.166385 -0.851935 0.000348

C -3.544175 -0.753214 0.000021

C -4.611571 0.070632 -0.000365

Br -3.754024 -2.629903 0.000237

H 2.337835 3.895557 -0.880015

H 4.872278 1.850157 0.000071

H -5.666896 2.938031 0.883329

H -5.620354 -0.313946 -0.000514

H 3.881555 3.818553 -0.001520

H 2.341575 3.895111 0.883556

H -6.562295 1.684054 -0.005420

H -5.661675 2.944482 -0.879654

H 0.628194 -0.369163 0.000151

H -1.240522 1.620706 0.000237

4’ unmodified U:U cWW

E = -908.616973313 A.U.

C -3.123901 -2.786215 0.000196

N -3.187455 -1.326661 -0.000380

C -1.981076 -0.637878 0.000197

O -0.916994 -1.247810 0.000715

N -2.075587 0.725022 0.000164

C -3.254305 1.493253 -0.000096

O -3.208157 2.705794 0.000098

C -4.465345 0.689232 -0.000589

C -4.383324 -0.651582 -0.000667

H -5.415612 1.196407 -0.000930

C 5.294640 -0.943190 -0.000438

N 4.058797 -0.165319 -0.000537

C 2.860717 -0.888765 0.000023

O 2.850733 -2.101471 0.000353

N 1.721339 -0.112378 0.000116

C 1.650096 1.276598 0.000335

O 0.567590 1.853378 0.000792

C 2.935444 1.939286 -0.000056

C 4.062139 1.201848 -0.000426

H 2.961311 3.015740 -0.000055

H -2.592424 -3.143767 -0.879509

H -5.261944 -1.280675 -0.001050

H 5.343029 -1.577059 0.882948

H 5.041743 1.658651 -0.000700

H -4.138302 -3.175304 -0.002390

H -2.597060 -3.143480 0.882850

H 6.136648 -0.256452 -0.005233

H 5.338355 -1.583792 -0.879112

H -1.177975 1.226741 0.000577

H 0.826684 -0.616313 0.000525

5’ 5BrU:A cS(r) H

E = -3955.87292189 A.U.

C -6.013037 0.957945 2.302818

N -4.840660 -2.863594 -0.896923

C -5.903138 -2.644770 -0.119606

N -6.133965 -1.617501 0.695589

C -5.136095 -0.735397 0.655119

C -3.975003 -0.812655 -0.111896

C -3.841487 -1.965661 -0.914666

N -2.778349 -2.207442 -1.699822

N -3.185273 0.305109 0.102887

C -3.855767 1.014182 0.980429

N -5.038546 0.445311 1.356711

C 2.755326 4.139165 -0.553420

C 1.590616 3.258831 -0.936998

O 2.098087 2.005734 -1.443591

C 0.657687 2.844179 0.207918

O -0.198813 3.884766 0.604650

C 0.028602 1.519020 -0.338067

O -1.138035 1.773816 -1.079185

C 1.142689 0.989128 -1.281884

N 1.858984 -0.212118 -0.767425

C 1.217524 -1.428686 -0.919677

O 0.119636 -1.533664 -1.441765

N 1.921290 -2.508581 -0.443090

C 3.181568 -2.533849 0.184022

O 3.660325 -3.578994 0.559481

C 3.760330 -1.200958 0.286526

C 3.097981 -0.126538 -0.185086

Br 5.466535 -1.028202 1.081086

H -6.986863 1.045167 1.824755

H -6.671760 -3.408093 -0.154486

H -2.743738 -3.104760 -2.151719

H -3.534460 1.959498 1.384619

H 2.376999 5.083093 -0.160314

H 3.387590 4.352731 -1.414727

H 0.989413 3.744680 -1.712700

H 1.256224 2.616051 1.092746

H -0.182552 0.803387 0.456609

H -1.862519 1.180208 -0.756160

H 0.697269 0.698513 -2.231965

H 3.508044 0.868697 -0.150271

H 3.365661 3.664886 0.216933

H -5.689323 1.936953 2.647135

H -6.104140 0.286234 3.154989

H -1.913570 -1.702498 -1.589603

H 1.466385 -3.404784 -0.545913

C -1.438925 4.388093 0.113577

H -1.441104 4.459537 -0.962392

H -2.257086 3.761781 0.426273

H -1.556394 5.372069 0.541325

5’ unmodified U:A cS(r) H

E = -1382.25484070 A.U.

C -5.158061 1.911342 1.730396

N -4.333371 -2.343769 -0.995924

C -5.427163 -1.862844 -0.401739

N -5.561031 -0.738996 0.300181

C -4.414166 -0.062373 0.351034

C -3.198223 -0.422456 -0.226722

C -3.184788 -1.649728 -0.923406

N -2.094563 -2.152788 -1.525055

N -2.242426 0.546960 0.028597

C -2.873330 1.446758 0.745611

N -4.183867 1.141450 0.978658

C 4.353302 3.215213 -0.293679

C 3.081801 2.512534 -0.704849

O 3.395097 1.145069 -1.040871

C 2.000352 2.389797 0.376926

O 1.321998 3.601959 0.600562

C 1.187791 1.143770 -0.110639

O 0.155764 1.519517 -0.988128

C 2.259593 0.329956 -0.885867

N 2.699327 -0.914696 -0.201281

C 1.862505 -2.013179 -0.316105

O 0.810617 -1.973777 -0.935662

N 2.311917 -3.147186 0.313352

C 3.497494 -3.318402 1.058688

O 3.748877 -4.392119 1.565507

C 4.298456 -2.114287 1.112076

C 3.881354 -0.993851 0.493073

H -6.014646 2.147056 1.101569

H -6.317994 -2.472256 -0.500652

H -2.175622 -3.078564 -1.908366

H -2.431586 2.352612 1.126448

H 4.126281 4.249385 -0.033639

H 5.080782 3.215482 -1.104927

H 2.641864 3.010156 -1.575854

H 2.474752 2.152614 1.331913

H 0.783232 0.564206 0.719208

H -0.688566 1.085561 -0.707045

H 1.847205 0.028832 -1.847401

H 4.457461 -0.083383 0.485874

H 4.802444 2.731835 0.575677

H -4.693094 2.833632 2.069694

H -5.506157 1.343249 2.591564

H -1.169373 -1.805124 -1.326921

H 1.710670 -3.955347 0.238733

H 5.233042 -2.143982 1.646847

C 0.225510 4.251739 -0.040099

H 0.314512 4.216278 -1.113495

H -0.708357 3.802798 0.251917

H 0.247858 5.279004 0.289693

6’ 5BrU:A cWH

E = -3534.75318181 A.U.

C -5.028415 2.426563 -0.000145

N -3.959258 -2.587989 0.000268

C -5.116138 -1.922103 0.000285

N -5.318256 -0.605171 0.000194

C -4.161896 0.059460 0.000041

C -2.882402 -0.489547 -0.000024

C -2.803676 -1.898871 0.000132

N -1.647314 -2.578103 0.000153

N -1.935559 0.517193 -0.000184

C -2.625969 1.631042 -0.000237

N -3.978245 1.425689 -0.000101

C 3.231426 3.557244 0.000434

N 2.712000 2.191134 0.000230

C 1.324302 2.038406 -0.000067

O 0.577143 2.999296 -0.000006

N 0.887310 0.736382 -0.000455

C 1.652275 -0.420426 -0.000324

O 1.130072 -1.524259 -0.000436

C 3.087018 -0.162982 -0.000056

C 3.543775 1.105185 0.000220

Br 4.276579 -1.631223 0.000005

H -5.655349 2.315981 -0.883499

H -6.007415 -2.539282 0.000388

H -1.699034 -3.581207 0.000151

H -2.189476 2.617402 -0.000354

H 2.883630 4.092150 -0.881035

H 4.600798 1.324734 0.000483

H -0.739941 -2.126539 -0.000139

H -0.150550 0.611907 -0.000413

H -4.573503 3.413960 -0.001005

H -5.654436 2.317116 0.884004

H 2.883780 4.091865 0.882139

H 4.316959 3.516667 0.000334

6’ un-modified U:A cWH

E = -961.134562414 A.U.

C -3.247771 3.154073 -0.000012

N -3.697314 -1.953889 0.000029

C -4.608077 -0.977945 0.000071

N -4.415565 0.340584 0.000057

C -3.114983 0.636964 0.000003

C -2.052818 -0.262928 -0.000041

C -2.389954 -1.633658 -0.000032

N -1.483035 -2.621051 -0.000097

N -0.852032 0.420511 -0.000051

C -1.184585 1.687449 -0.000023

N -2.538378 1.888954 0.000012

C 5.024508 1.717219 0.000020

N 4.110741 0.578427 -0.000021

C 2.742479 0.864029 -0.000012

O 2.332781 2.010907 0.000001

N 1.921026 -0.235684 -0.000014

C 2.303077 -1.570657 0.000014

O 1.462945 -2.464205 0.000000

C 3.736034 -1.778952 0.000045

C 4.560471 -0.714251 0.000029

H 4.114062 -2.787346 0.000077

H -3.878390 3.233968 -0.884136

H -5.641165 -1.306805 0.000123

H -1.827540 -3.564563 0.000021

H -0.478137 2.502590 -0.000020

H 4.860223 2.334414 -0.881192

H 5.635587 -0.825388 0.000043

H -0.481411 -2.456552 -0.000058

H 0.899639 -0.032485 -0.000033

H -2.522539 3.964047 0.000815

H -3.879613 3.233221 0.883294

H 4.860202 2.334366 0.881264

H 6.044895 1.343881 0.000020

7’ 5BrU:A cW(r)S

E = -3955.87311908 A.U.

C -1.829466 3.110560 0.783188

O -2.163792 1.896930 1.484972

C -0.989472 2.656938 -0.417558

O -0.251598 3.701620 -0.999708

C -0.224781 1.425924 0.176600

O 0.931527 1.836492 0.859538

C -1.245605 0.874459 1.199883

N -2.018030 -0.307831 0.703079

C -1.391474 -1.538427 0.765856

O -0.255062 -1.683276 1.189880

N -2.151361 -2.592225 0.319557

C -3.462363 -2.579778 -0.193845

O -3.990592 -3.605774 -0.555262

C -4.023882 -1.235907 -0.205313

C -3.302761 -0.187542 0.237668

Br -5.790231 -1.012787 -0.838233

C 8.039456 -0.339455 -1.225814

N 3.140033 0.324397 0.157774

C 4.015509 1.136855 -0.462185

N 5.274947 0.896167 -0.789559

C 5.652485 -0.332802 -0.419717

C 4.865909 -1.281346 0.225249

C 3.538362 -0.913352 0.517693

N 2.691728 -1.742744 1.141525

N 5.574196 -2.442398 0.448270

C 6.755881 -2.190880 -0.050250

N 6.875180 -0.931010 -0.593163

H -1.231107 3.765548 1.420362

H -1.649246 2.310705 -1.215045

H 0.013892 0.681049 -0.583360

H 1.690036 1.224314 0.661557

H -0.728090 0.547362 2.098978

H -3.699646 0.813089 0.272362

H 7.825463 -0.096490 -2.265569

H 3.629482 2.113705 -0.723858

H 3.001411 -2.679031 1.332984

H 7.587659 -2.877598 -0.055829

H 1.706539 -1.535651 1.216151

H 8.861008 -1.050403 -1.184738

H 8.327530 0.573847 -0.707969

H -1.706025 -3.498220 0.359608

C -3.146745 3.814384 0.421444

H -3.889220 3.598111 1.173186

H -2.991552 4.880564 0.375852

H -3.501428 3.469517 -0.537532

C 1.009977 4.289447 -0.683997

H 1.107559 4.477478 0.372541

H 1.814943 3.653270 -1.010493

H 1.051983 5.223486 -1.222199

7’ unmodified U:A cW(r)S

E = -1382.25585410 A.U.

C 3.183396 2.530471 -0.607134

O 3.408080 1.238802 -1.201925

C 2.222264 2.267260 0.558839

O 1.590640 3.429443 1.029282

C 1.342931 1.106248 -0.012128

O 0.294359 1.616863 -0.795497

C 2.345491 0.360314 -0.924808

N 2.920342 -0.872286 -0.308455

C 2.144251 -2.017240 -0.359931

O 1.035500 -2.039869 -0.875115

N 2.716095 -3.128755 0.205716

C 3.974187 -3.236797 0.834753

O 4.333421 -4.298231 1.300617

C 4.705902 -1.988186 0.828672

C 4.166729 -0.889647 0.267360

H 5.688296 -1.967650 1.270027

C -7.162690 0.447941 0.838250

N -2.133167 0.424729 -0.193680

C -2.940890 1.374468 0.311644

N -4.240407 1.309406 0.554072

C -4.741906 0.113616 0.224468

C -4.037284 -0.961978 -0.305634

C -2.656578 -0.776580 -0.514835

N -1.878865 -1.740277 -1.024024

N -4.867487 -2.041922 -0.515525

C -6.039841 -1.619204 -0.121776

N -6.038033 -0.322171 0.340826

H 2.708700 3.203752 -1.324899

H 2.785046 1.895899 1.417155

H 0.962329 0.450317 0.771663

H -0.544475 1.111467 -0.626969

H 1.847132 0.047188 -1.839317

H 4.689113 0.051364 0.214756

H -6.929432 0.858806 1.818735

H -2.454550 2.312402 0.547762

H -2.289711 -2.642328 -1.187501

H -6.948513 -2.200174 -0.138560

H -0.871964 -1.659955 -1.034660

H -8.028302 -0.205093 0.918544

H -7.394427 1.270819 0.162995

H 2.158368 -3.970302 0.174999

C 4.549615 3.099979 -0.200660

H 5.288036 2.830363 -0.940066

H 4.491757 4.175486 -0.134948

H 4.846431 2.704109 0.759114

C 0.431398 4.145234 0.607751

H 0.411845 4.279041 -0.462185

H -0.464052 3.633603 0.920630

H 0.481187 5.108369 1.092336

8’ IU:A cWW

E = -971.944910232 A.U.

C -7.298141 0.572812 -0.019912

N -2.179854 0.340914 0.001529

C -3.022893 1.386797 0.002719

N -4.348527 1.366876 0.002202

C -4.821392 0.115182 0.001886

C -4.073816 -1.058982 0.001474

C -2.672417 -0.914688 0.001666

N -1.831841 -1.957011 0.001872

N -4.893388 -2.168000 0.004478

C -6.100688 -1.667771 0.005887

N -6.133444 -0.291123 0.007538

C 2.737538 3.923203 0.001650

N 2.327045 2.520619 0.001106

C 0.953092 2.262828 0.001459

O 0.137297 3.163369 0.002154

N 0.617662 0.929829 0.000924

C 1.474071 -0.160869 0.000084

O 1.038397 -1.304070 -0.000436

C 2.885355 0.200900 -0.000290

C 3.239400 1.502837 0.000224

I 4.318615 -1.324455 -0.001688

H -7.159172 1.393399 0.680503

H -2.543908 2.358915 0.003909

H -2.213141 -2.885456 0.002060

H -7.012627 -2.243968 0.010519

H 2.348028 4.429396 -0.879333

H 4.274436 1.810502 -0.000037

H -0.825761 -1.812747 0.001436

H -0.408483 0.720384 0.001117

H 3.822988 3.967990 0.001243

H 2.348625 4.428506 0.883393

H -8.175032 -0.002584 0.267859

H -7.452335 0.989167 -1.015583

8’ unmodified U:A cWW

E = -961.133397377 A.U.

C 5.487720 -1.737522 -0.014760

N 0.643271 -0.069741 0.001001

C 1.156826 -1.310215 0.004349

N 2.434630 -1.666256 0.004769

C 3.241157 -0.598823 0.002799

C 2.855873 0.738643 -0.000190

C 1.470258 0.995765 -0.000594

N 0.956769 2.232133 -0.002626

N 3.955730 1.570903 0.001407

C 4.972485 0.749982 0.004562

N 4.614775 -0.579775 0.008539

C -5.155801 -2.008364 0.000023

N -4.340943 -0.796857 -0.000259

C -2.952301 -0.969002 0.000464

O -2.451379 -2.076812 0.001510

N -2.223964 0.196707 -0.000086

C -2.719232 1.493938 -0.001158

O -1.959589 2.458643 -0.001498

C -4.163785 1.582170 -0.001812

C -4.896917 0.452444 -0.001361

H -4.623713 2.555907 -0.002632

H 5.132962 -2.476796 0.700153

H 0.423349 -2.108130 0.006466

H 1.584312 3.015417 -0.003057

H 6.010130 1.044904 0.008660

H -4.939259 -2.610250 -0.880484

H -5.977621 0.473926 -0.001813

H -0.050875 2.376951 -0.002209

H -1.186975 0.082867 0.000332

H -6.203940 -1.721768 -0.000802

H -4.940355 -2.609146 0.881550

H 6.494996 -1.428797 0.255389

H 5.505154 -2.193621 -1.004783

9’ IU:G cWW

E = -1047.21214804 A.U.

C -7.612014 -0.664510 -0.053469

N -2.787374 0.809814 0.049792

C -3.926457 1.558613 0.037677

N -3.761719 2.913964 0.106159

N -5.133621 1.049980 0.004609

C -5.125518 -0.301213 -0.002075

C -4.034050 -1.167483 0.007725

C -2.723680 -0.599510 0.031007

O -1.627758 -1.155241 0.034990

N -4.455633 -2.479074 -0.011120

C -5.754854 -2.402910 -0.031212

N -6.231065 -1.103942 -0.026339

C 1.977624 3.595475 -0.042790

N 2.021525 2.134732 -0.023741

C 0.814323 1.455913 -0.009597

O -0.248154 2.079209 -0.013174

N 0.895171 0.095529 0.008770

C 2.063312 -0.689325 0.012915

O 2.005032 -1.895598 0.029481

C 3.288913 0.112991 -0.004988

C 3.217036 1.455181 -0.021988

I 5.126398 -0.886277 -0.002088

H -4.579127 3.454970 -0.116264

H -6.433193 -3.241187 -0.048177

H 1.473626 3.949162 -0.940305

H 4.098646 2.077681 -0.034930

H -8.258072 -1.535457 0.027070

H -7.811766 0.007350 0.779719

H -7.833584 -0.140289 -0.982811

H 1.437662 3.968596 0.824949

H 2.996516 3.971241 -0.026595

H -0.008888 -0.416087 0.020337

H -1.877111 1.280264 0.068915

H -2.875532 3.303155 -0.167762

9’ unmodified U:G cWW

E = -1036.40069105 A.U.

C -5.923056 0.385992 0.032088

N -0.888292 0.701425 -0.043305

C -1.822568 1.693340 -0.036275

N -1.347447 2.973432 -0.105190

N -3.115296 1.478852 -0.007763

C -3.419958 0.162341 0.000921

C -2.559287 -0.933344 -0.003794

C -1.151983 -0.685101 -0.022719

O -0.214503 -1.478092 -0.022037

N -3.273836 -2.111311 0.016598

C -4.520121 -1.735969 0.032161

N -4.681847 -0.362051 0.024929

C 4.355289 2.381162 0.039763

N 4.088971 0.945611 0.021966

C 2.763289 0.539389 0.014879

O 1.860569 1.379141 0.023528

N 2.547447 -0.805502 -0.002694

C 3.531279 -1.816580 -0.011912

O 3.221487 -2.987421 -0.027197

C 4.891227 -1.300833 -0.000868

C 5.109128 0.022870 0.015020

H -5.962088 1.052665 0.892489

H -5.374446 -2.393998 0.049729

H 3.897575 2.863191 -0.821742

H 5.704379 -2.007303 -0.006830

H 6.104335 0.443406 0.022843

H 0.106584 0.949743 -0.058382

H 1.557629 -1.110929 -0.009532

H -6.017509 0.983681 -0.873919

H -6.753067 -0.314488 0.085435

H 5.430715 2.532011 0.011100

H 3.949379 2.835095 0.942214

H -0.394711 3.145188 0.168259

H -2.016535 3.689914 0.116606
